# Loss of *β*‐Actin Leads to Accelerated Mineralization and Dysregulation of Osteoblast‐Differentiation Genes during Osteogenic Reprogramming

**DOI:** 10.1002/advs.202002261

**Published:** 2020-10-27

**Authors:** Tamara Gjorgjieva, Xin Xie, Patrick Commins, Renu Pasricha, Syed Raza Mahmood, Kristin C. Gunsalus, Panče Naumov, Piergiorgio Percipalle

**Affiliations:** ^1^ Program in Biology Division of Science and Mathematics New York University Abu Dhabi (NYUAD) P.O. Box 129188 Abu Dhabi United Arab Emirates; ^2^ Center for Genomics and Systems Biology New York University Abu Dhabi (NYUAD) P.O. Box 129188 Abu Dhabi United Arab Emirates; ^3^ Program in Chemistry Division of Science and Mathematics New York University Abu Dhabi (NYUAD) P.O. Box 129188 Abu Dhabi United Arab Emirates; ^4^ Core Technology Platforms New York University Abu Dhabi (NYUAD) P.O. Box 129188 Abu Dhabi United Arab Emirates; ^5^ Department of Biology New York University New York NY 10003 USA; ^6^ Department of Molecular Biosciences The Wenner‐Gren Institute Stockholm University Stockholm SE‐106 91 Sweden

**Keywords:** *β*‐actin, mineralization, mitochondria, osteogenesis, transcriptional reprogramming

## Abstract

Actin plays fundamental roles in both the cytoplasm and the cell nucleus. In the nucleus, *β*‐actin regulates neuronal reprogramming by consolidating a heterochromatin landscape required for transcription of neuronal gene programs, yet it remains unknown whether it has a role in other differentiation models. To explore the potential roles of *β*‐actin in osteogenesis, *β*‐actin wild‐type (WT) and *β*‐actin knockout (KO) mouse embryonic fibroblasts (MEFs) are reprogrammed to osteoblast‐like cells using small molecules in vitro. It is discovered that loss of *β*‐actin leads to an accelerated mineralization phenotype (hypermineralization), accompanied with enhanced formation of extracellular hydroxyapatite microcrystals, which originate in the mitochondria in the form of microgranules. This phenotype is a consequence of rapid upregulation of mitochondrial genes including those involved in oxidative phosphorylation (OXPHOS) in reprogrammed KO cells. It is further found that osteogenic gene programs are differentially regulated between WT and KO cells, with clusters of genes exhibiting different temporal expression patterns. A novel function for *β*‐actin in osteogenic reprogramming through a mitochondria‐based mechanism that controls cell‐mediated mineralization is proposed.

## Graphical abstract

The present study highlights a new function for *β*‐actin in transcriptional reprograming during osteogenic differentiation, leading to cell‐mediated mineralization. *β*‐actin performs this task by regulating expression of osteogenic factors and mitochondrial genes during osteogenesis. These observations support the novel involvement of cytoskeletal proteins such as actin in gene expression regulation during development and differentiation.

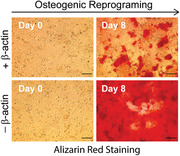

*β*‐actin plays essential roles in many cytoplasmic processes, including cell division, vesicle trafficking, and motility,^[^ ^1^, ^2^ ^]^ and it has recently emerged as a critical player in the nucleus. *β*‐actin is involved in chromatin and transcription regulation,^[^ ^3^ ^]^ regulating both nuclear‐encoded mitochondrial and developmental genes,^[^ ^4^, ^5^, ^6^ ^]^ and plays a role in the dynamic changes in transcriptional patterns that occur during differentiation.^[^ ^5^, ^6^ ^]^ We recently reported that direct reprogramming of *β*‐actin knockout (KO) mouse embryonic fibroblasts (MEFs) to neurons fails to induce the expression of a large portion of neuronal genes in comparison to the wild‐type (WT) condition, primarily due to increased heterochromatin levels at transcription start sites of neuronal genes in *β*‐actin KO.^[^ ^7^ ^]^ It remains unknown whether *β*‐actin plays a role in other cell differentiation processes; so in this study, we explored the roles of *β*‐actin in osteogenic reprogramming.

The osteoblast is the major cell type contributing to bone formation. Osteoblast differentiation (OBD) is a highly orchestrated process: osteoblasts differentiated from mesenchymal stem cells rely on the activation of specific signaling proteins and transcription factors (TFs), which induce the expression of downstream factors required for osteogenesis, including Runx2, osteocalcin, osteopontin, and alkaline phosphatase (ALPL).^[^ ^8^ ^]^ Bone morphogenetic protein (BMP) and Wingless and Int‐1 (Wnt)/*β*‐catenin signaling are the major drivers of OBD.^[^ ^9^ ^]^ Runx2 is considered the master regulator of the osteoblast phenotype in vivo;^[^ ^10^ ^]^ however, studies of in vitro differentiation of human osteoblasts reported no major changes in Runx2 expression, despite increased expression levels of downstream genes such as ALPL.^[^ ^11^ ^]^ Upon differentiation, mature osteoblasts synthesize and deposit type I collagen (osteoid), as well as the calcium‐binding proteins, osteocalcin and osteopontin, thereby creating the organic bone matrix.^[^ ^12^ ^]^ Osteoblasts also secrete calcium and phosphate, which crystallize into hydroxyapatite (HA) Ca_5_(PO_4_)_3_(OH) and help mineralize the extracellular matrix (ECM).^[^ ^13^ ^]^ Some osteoblasts then differentiate into osteocytes and become embedded in the mineralized matrix.^[^ ^12^, ^13^ ^]^ Recent studies have demonstrated that mitochondria play an important role in the mineralization process: calcium phosphate granules originate in the mitochondrial matrix and are subsequently transported via vesicles from mitochondria to the ECM to facilitate mineralization in osteoblast cells.^[^ ^14^, ^15^ ^]^

To explore the potential role of *β*‐actin in osteogenic reprogramming, we first analyzed published RNA‐seq data on WT (*β*‐actin ^+/+^), heterozygous (HET, *β*‐actin ^+/−^), and KO (*β*‐actin ^−/−^) MEFs. Following gene ontology (GO) analysis on differentially expressed (DE) genes, we found that gene programs related to osteogenesis and calcium homeostasis are dysregulated between the three conditions (Figure S1A–D, Supporting Information), and that a majority of DE genes involved in calcium ion binding are upregulated in the KO condition (Figure S1D, Supporting Information). These findings suggested that *β*‐actin may play a role in osteogenic reprogramming, so we next sought to establish a protocol to reprogram MEFs to osteoblast‐like cells and study this further. We tested the effect of various chemical cocktails in basal OsteoMAX‐XF differentiation medium, using different combinations of dexamethasone (Dex)—a molecule that stimulates ALPL activity and mRNA levels of osteopontin and osteocalcin;^[^ ^16^ ^]^ forskolin (F)—an adenylyl cyclase activator;^[^ ^17^ ^]^ and CHIR99021 (Chir) )—a GSK3 inhibitor that induces the Wnt/*β*‐catenin pathway^[^ ^18^ ^]^ (Figure S2, Supporting Information). The Dex/F combination showed effective induction of a functional osteoblastic phenotype: it mineralized the ECM after 14 days and induced gene expression of osteogenic biomarkers such as Bglap (osteocalcin), ALPL, Spp1 (osteopontin), Fgf23, Sost (sclerostin), and DMP1 (Figure S2C,D, Supporting Information), and was therefore used for all subsequent experiments.

We reprogramed WT, HET, and KO MEFs to osteoblast‐like cells using the Dex/F chemical cocktail, and examined the effect of varying levels of *β*‐actin on osteogenic reprogramming (**Figure** **1**A) by monitoring the morphology (microscopy), calcium‐based mineralization (Alizarin Red staining), and gene expression of key osteogenic biomarkers (quantitative polymerase chain reaction (qPCR)) over 14 days. Compared to the cuboidal morphology of reprogrammed WT cells, both HET and KO exhibited dendrite‐like cellular features at days 8 and 14 (Figure 1B, black arrows). Furthermore, qPCR analysis showed significant induction of genes related to osteoblast function, such as Bglap (osteocalcin), Spp1 (osteopontin), and Sost (sclerostin) in WT, HET, and KO cells at days 4 or 14 (Figure 1C). However, the osteoblast TF Runx2 was not upregulated, indicating that the direct reprogramming process may not entirely depend on Runx2 expression. We also observed differences in the expression levels of some osteogenic markers in HET and KO cells compared with WT cells (Figure 1C). Strikingly, we found that loss of *β*‐actin was associated with hypermineralization (accelerated mineralization): while WT cells took 14 days to fully mineralize the well, HET and KO cells took 8 and 4 days, respectively, (Figure 1D,E). These results indicate that the rate of calcium‐based mineralization in reprogrammed cells is *β*‐actin dosage‐dependent, implying that loss of *β*‐actin accelerates ECM mineralization during osteogenic reprogramming.

**Figure 1:**
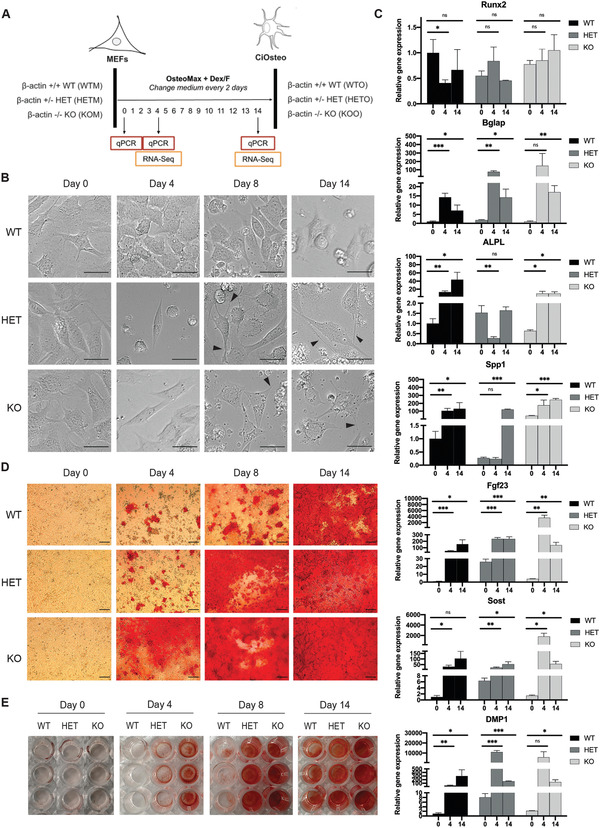
Loss of *β*‐actin leads to accelerated mineralization during osteogenic reprogramming in a dosage‐dependent manner. A) Experimental setup for direct reprogramming of MEFs (M) to osteoblast‐like (O) cells for *β*‐actin ^+/+^ (WT, WTM to WTO), ^+/−^ (HET, HETM to HETO), and ^−/−^ (KO, KOM to KOO). B) Cell morphology of WT, HET, and KO cells during osteogenic reprogramming at days 0, 4, 8, and 14. Scale bar: 30 µm. Arrows indicate dendrite‐like cellular features at days 8 and 14 in HET and KO cells. C) qPCR quantification of relative gene expression of key osteogenic marker genes in WT, HET, and KO cells at days 0, 4, and 14. The Nono housekeeping gene was used for expression normalization for each gene, and normalized expression of WTM was set to 1 for each gene. Data are the summary of three biological replicates and four technical replicates. Data presented as mean ± SD. *p*‐Values are calculated using Welch *t*‐test, *p < 0.05, **p < 0.01, ***p < 0.001. D) Alizarin Red staining for calcium mineralization during reprogramming. Scale bar: 100 µm. E) Images of Alizarin Red staining in whole wells of a 96‐well plate. Three biological replicates are shown in each condition.

The hypermineralization phenotype in KO cells at day 4 was also accompanied with the formation of extracellular crystal‐like structures in KO cells at day 4 (**Figure** **2**C, red arrows), which was not the case for WT cells at either day 4 (Figure 2A) or day 14 (Figure S3A, Supporting Information). To study the chemical nature of these structures, we fixed WT and KO cells at day 4 and isolated the insoluble inorganic component. We then used scanning electron microscopy (SEM) and elemental composition analysis by energy‐dispersive X‐ray (EDX) spectroscopy to image and characterize these chemical structures. In the KO sample, SEM revealed a polycrystalline structure consisting of many micro‐level columns, and the EDX mapping indicated the presence of C, Ca, P, and O on the microcrystal surface (Figure 2D,F). In contrast, the WT sample at day 4 contained amorphous and highly irregular structures, and EDX mapping also showed the presence of C, Ca, P, and O (Figure 2B,E). In the WT sample at day 14, we observed a nascent crystal‐like structure formation, although still irregular compared to the KO sample at day 4 (Figure S3B,C, Supporting Information). Considering that osteoblasts secrete HA during osteogenesis,^[^ ^12^ ^]^ as well as the fact that HA has the chemical formula Ca_5_(PO_4_)_3_(OH) and tends to crystallize in a regular hexagonal shape,^[^ ^19^ ^]^ our SEM–EDX data indicate that the hypermineralization phenotype in *β*‐actin KO is characterized by an increased formation of HA.

**Figure 2:**
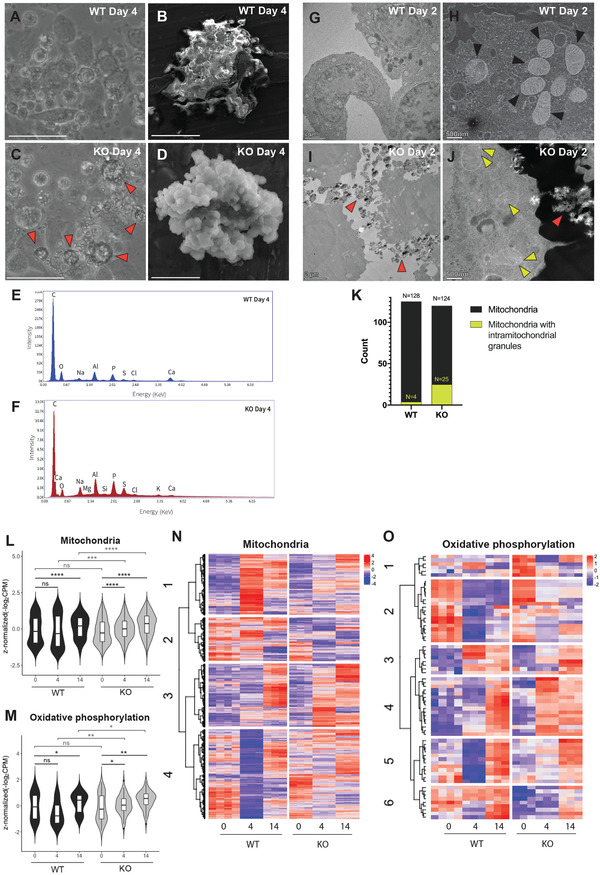
*β*‐actin ^−/−^ (KO) cells secrete HA‐like microcrystals and show enhanced intramitochondrial granules in the early phase of reprogramming. A,C) Live microscopy images of WT and KO cells on day 4. C) Red arrowheads highlight the extracellular crystalline‐like structures in KO cells, A) which are not observed in WT cells (scale bar: 50 µm). B,D) SEM images of the inorganic extracellular components in WT and KO cells on day 4. D) The KO sample shows distinct crystallites and the EDX captured at the structures indicates the presence of Ca (scale bar: 5 µm), B) whereas the WT shows an amorphous structure without any distinct crystallites (scale bar: 50 µm). E,F) EDX spectra on the sample surface in WT and KO cells, showing emission energy (*x*‐axis) against relative signal intensity (*y*‐axis). All elements of HA (Ca, P, and O) are identified. Na, Mg, Al, and Si are trace elements from the SEM plate on which the sample was deposited. G–J) TEM images of WT and KO cells at day 2. I) Red arrowheads highlight the extracellular microcrystals structure (scale bar: 2 µm), and J) yellow arrowheads highlight the intramitochondrial granules in KO (scale bar: 500 nm). G,H) No similar structures are observed in WT; H) black arrowheads highlight the mitochondria. K) Quantification of mitochondria with granules in the WT and KO condition at day 2. Counts are based on the TEM images of 24 WT and 22 KO cells, respectively. L,M) Violin plots of VST‐normalized, *z*‐score‐normalized gene expression data showing L) 1136 mitochondria and M) 68 OXPHOS genes between different timepoints. *p*‐Values calculated using matched‐pairs two‐tailed *t*‐test, *p < 0.05, **p < 0.01, ***p < 0.001, ****p < 0.0001, ^ns^ *p* > 0.05. Box plots display the median and interquartile range. N,O) Heatmap of *z*‐score‐normalized expression data for genes that are DE (*p*‐adjusted < 0.05) between WT4 and KO4 and part of the mitochondria (*n* = 1136) and OXPHOS GO terms (*n* = 68). Hierarchical clustering was performed using Ward's linkage using N) *k* = 4 clusters for mitochondria and O) *k* = 6 for OXPHOS.

Previous studies have identified mitochondria as the origin of cell‐mediated mineralization,^[^ ^14^, ^15^ ^]^ so we next used transmission electron microscopy (TEM) to examine mitochondrial structures in WT and KO cells. We prepared WT and KO samples at day 2 (WT2 and KO2) for TEM, since excessive mineralization in KO cells at day 4 did not allow us to capture clear cellular structures (data not shown). TEM data show that KO2 cells are embedded within extracellular HA‐like microcrystals (Figure 2I, red arrows), while WT2 cells are not (Figure 2G). In KO cells, we also detected intramitochondrial granules similar to the calcium phosphate granules observed in mitochondria of osteoblasts in bone nodules (Figure 2J, yellow arrows),^[^ ^14^, ^15^ ^]^ and these granules occurred at a much higher rate in KO2 compared to WT2 cells (Figure 2H,J,K; Figure S4, Supporting Information). It is, therefore, likely that hypermineralization of the ECM in KO cells is due to enhanced calcium phosphate granule formation in the mitochondria.

To investigate this further, we performed RNA‐seq to study changes in mitochondrial gene expression between WT and KO cells, since it is known that increased mitochondrial activity and biogenesis is required for ECM mineralization during OBD.^[^ ^20^ ^]^ We focused on genes that are DE between WT4 and KO4 (*p*‐adjusted < 0.05), as day 4 is when we observed the most drastic differences in mineralization. Of the 10 400 DE genes, we found 1136 nuclear‐encoded genes related to mitochondria (GO:0005739) and 68 genes involved in oxidative phosphorylation (OXPHOS, GO:0006119) (Table S1A,B, Supporting Information). While the overall expression level of mitochondrial genes showed no difference between WT and KO cells on day 0, we detected significant upregulation of mitochondrial genes between days 0 and 4 in the KO cells (*p* < 0.0001), but not in the WT condition (Figure 2L; Table S1C, Supporting Information). A similar trend was observed for OXPHOS genes (Figure 2M). Therefore, the increased level of mitochondrial gene expression in the first 4 days of KO cells may support accelerated mitochondria‐dependent mineralization. Meanwhile, both mitochondrial and OXPHOS genes were significantly upregulated in both the WT and KO conditions by day 14 in comparison with day 0 (Figure 2L,M), consistent with the notion of increased mitochondrial activity during osteogenic differentiation.^[^ ^20^ ^]^

We next plotted a heatmap of the 1136 mitochondrial genes and identified four clusters (*k* = 4, see Experimental Section, Figure S5A, and Table S1A, Supporting Information) with similar expression patterns (Figure 2N). Genes in the two biggest clusters, clusters 3 (*n* = 279) and 4 (*n* = 395), were not induced in WT at day 4, but most were eventually induced by day 14 (Figure 2N). GO analysis further revealed that these two clusters are enriched for genes involved in OXPHOS (*n* = 27 in cluster 3, and *n* = 36 in cluster 4), whereas clusters 1 and 2 are not (Table S1D, Supporting Information). Consistently, analysis of the 68 OXPHOS genes revealed different clusters of genes (*k* = 6, see Experimental Section, Figure S5B, and Table S1B, Supporting Information), with genes in clusters 2, 4, and 5 showing evidently lower expression in WT at day 4. Some of these genes (clusters 4 and 5) seem to get induced by day 14, while expression of genes in cluster 2 remains low. Overall, our data show an earlier and stronger induction of mitochondrial genes in KO cells (including OXPHOS genes that are functioning in the main activity in mitochondria for ATP production), which may account for the hypermineralization in KO cells during the initial phase of osteogenic reprogramming.

We last sought to understand the temporal changes in the expression of genes related to the differentiation of the osteoblast lineage. Principal component (PC) analysis of the RNA‐seq data shows that PC1 separated MEF samples (day 0) from the reprogrammed cells, and PC2 distinguished the WT from the KO condition (Figure S6A, Supporting Information). We analyzed DE genes between days 0–4 and days 4–14 in both WT and KO condition (Figure S6B and Table S2A, Supporting Information), and GO analysis showed that these DE genes were enriched for positive or negative regulation of OBD, Wnt, and BMP signaling pathways (Figure S6C–E and Table S2B,C, Supporting Information). Interestingly, we found that numerous genes involved in positive and negative regulation of OBD are differentially regulated between the WT and KO conditions (Figure S6C, Supporting Information), prompting us to further analyze the temporal expression of OBD‐related genes (*n* = 202, GO:0001649).

To focus on genes with significant changes, we selected OBD genes that were DE between WTM‐KOM, WT4‐KO4, or WT14‐KO14 (Figure S7A, Supporting Information), which resulted in 96 genes. Hierarchical clustering (*k* = 5, see Experimental Section, Figure S7A,B, Supporting Information) revealed distinctive temporal expression patterns between WT and KO cells (**Figure** **3**). For example, genes in cluster 1 are strongly expressed in KOM (day 0), but are barely expressed in WT conditions; interestingly, this cluster is enriched for genes involved in the positive regulation of OBD (Figure 3, red squares). In contrast, cluster 5 contains genes that are underrepresented in KO4 relative to WT4, and many of these genes are involved in the negative regulation of OBD (Figure 3, blue squares). These differences in the expression of OBD regulatory genes may favor accelerated osteogenic programming in KO cells. In another example, genes in clusters 2 and 4 are similarly regulated during the course of reprogramming in both the WT and KO conditions, while genes in cluster 3, which contain both positive and negative regulators of OBD, are preferentially expressed in WT. Altogether, these data suggest that OBD reprogramming could potentially take different regulatory trajectories in WT and KO cells, and loss of *β*‐actin differentially affects sets of OBD‐related genes prior to or during reprogramming in a manner consistent with the early onset of mineralization.

**Figure 3:**
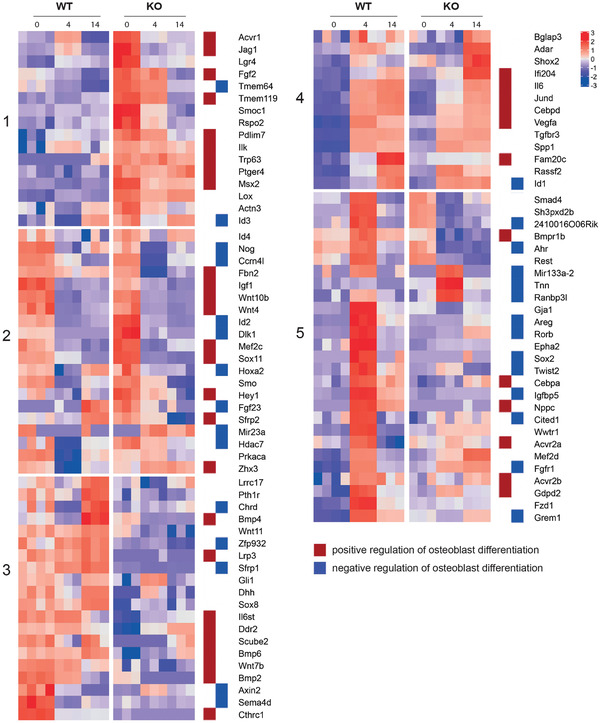
Loss of *β*‐actin leads to dysregulation of temporal expression of OBD genes. Gene expression data for 96 OBD genes that are significantly DE (*p*‐adjusted < 0.05, |fold change| > 2) in at least one between‐groups pairwise comparison (WTM vs KOM, WT4 vs KO4, WT14 vs KO14). Heatmap is based on *z*‐score‐normalized data and shows temporal gene expression patterns. Hierarchical clustering was performed using Ward's linkage using *k* = 5 clusters. Red and blue squares identify whether the gene is involved in positive regulation of OBD (red) or negative regulation of OBD (blue).

Similar analyses focusing on DE genes in Wnt (*n* = 183, GO:0016055) and Bmp (*n* = 74, GO:0030509) pathways also reveal clusters of genes (Figure S7C,D and Table S3B,C, Supporting Information) with distinctive temporal patterns (Figure S7E,F, Supporting Information), further indicating that loss of *β*‐actin dysregulates the major signaling cascades driving OBD.^[^ ^21^ ^]^ Additionally, GO enrichment analysis of DE genes between WT and KO conditions also revealed the significant overrepresentation of genes involved in Wnt and Bmp signaling pathways (Figure S8, Supporting Information). These data imply that loss of *β*‐actin seems to also affect these two signaling processes prior to and during the course of reprogramming.

In summary, using an in vitro protocol to reprogram MEFs into functional osteoblast‐like cells, we report a novel function of *β*‐actin in regulating the ECM mineralization during osteogenesis. This primarily stems from evidence that the expression of genes involved in OBD and mitochondrial function are dysregulated upon loss of *β*‐actin, an observation compatible with the emerging role of *β*‐actin in transcriptional regulation during differentiation.^[^ ^5^, ^7^ ^]^ Previous studies demonstrated that calcium ions and inorganic phosphate accumulate in mitochondria at an averaged ratio of 1.67 (the same ratio as in the HA), and that electron dense particles mainly composed of HA are formed in the mitochondrial matrix.^[^ ^14^, ^22^, ^23^ ^]^ These results suggest that mitochondria play a central role in cell‐mediated mineralization. In the absence of *β*‐actin, we observed enhanced formation of intramitochondrial electron dense granules at the early stage of reprogramming, suggesting that mitochondrial HA formation occurs at a faster pace in the KO condition compared to WT. This is likely to be due to a combination of dysregulated mitochondrial calcium uptake and increased mitochondrial activity and oxygen consumption as revealed by increased mitochondrial and OXPHOS gene expression in the early phase of reprogramming.^[^ ^20^, ^23^, ^24^ ^]^ We, therefore, propose that OBD is tightly linked with mineralization driven by mitochondrial activity, and that *β*‐actin plays a key role in osteogenic reprogramming by titrating the expression of mitochondrial genes involved in OBD.

Actin plays important and diverse roles in cellular biology. Beyond its wide array of cytoskeletal functions, actin can exert broad‐ranging influence over signaling pathways involving cellular differentiation programs through both cytoplasmic and nuclear mechanisms. The cortical actin cytoskeleton provides a scaffold for and can spatially confine signaling complexes,^[^ ^3^, ^5^, ^25^, ^26^, ^27^ ^]^ and changes in the relative proportion of actin isoforms alter cell surface features that affect both biophysical properties^[^ ^28^ ^]^ and TGF‐*β* growth factor signaling.^[^ ^29^ ^]^ Nuclear actin influences transcription and chromatin remodeling, with consequent effects on cellular identity,^[^ ^30^ ^]^ and can directly affect signaling pathways at the transcriptional level. For example, nuclear actin influences Wnt/*β*‐catenin signaling by enhancing the nuclear accumulation and binding of *β*‐catenin to its transcriptional targets.^[^ ^31^ ^]^ Therefore, actin can affect the spatiotemporal dynamics of signaling pathways via multiple routes.

Here, we show that changes in the levels of *β*‐actin have a significant impact on the regulation of mitochondrial and OBD genes during osteogenic reprogramming. While the precise mechanisms remain to be characterized, the changes of key signaling pathways involved in OBD (Bmp/TGF‐*β* and Wnt) may contribute to the differential OBD gene regulation and phenotypes in the absence of *β*‐actin. Our findings that *β*‐actin strongly influences the transcriptional landscape during osteogenic reprogramming add to accumulating evidence that *β*‐actin plays a key role in transcriptional reprogramming during differentiation and emphasize the contribution of cytoskeletal proteins to cell physiology and cell fate.

## Conflict of Interest

The authors declare no conflict of interest.

## Supporting information

Supporting Information
