# Supplementary material for: Loss of β‐Actin Leads to Accelerated Mineralization and Dysregulation of Osteoblast‐Differentiation Genes during Osteogenic Reprogramming
Source: Adv Sci (Weinh). 2020 Oct 27;7(23):2002261. doi: 10.1002/advs.202002261 (PMC7709978; doi:10.1002/advs.202002261)
Supplement: Supplementary file 1 — Supporting Information [file ADVS-7-2002261-s001.pdf]

© 2020 Wiley-VCH GmbH

ADVANCED SCIENCE
Open Access

Supporting Information

for *Adv. Sci.*, DOI: 10.1002/advs.202002261

Loss of β-actin Leads to Accelerated Mineralization and Dysregulation of Osteoblast-differentiation Genes during Osteogenic Reprogramming

[1,*]Tamara Gjorgjieva, [1,2*]Xin Xie, [3]Patrick Commins, [4]Renu Pasricha, [1,5] S. Raza Mahmood, [2,5,]Kristin C. Gunsalus, [3]Pance Naumov and [1,6]Piergiorgio Percipalle

Supplementary Information

Loss of β-actin leads to accelerated mineralization and dysregulation of osteoblast-differentiation genes during osteogenic reprogramming

[1,*]Tamara Gjorgjieva, [1,2*]Xin Xie, [3]Patrick Commins, [4]Renu Pasricha, [1,5] S. Raza Mahmood, [2,5,]Kristin C. Gunsalus, [3]Pance Naumov and [1,6]Piergiorgio Percipalle

[1]Science Division, Biology Program, New York University Abu Dhabi (NYUAD), P.O. Box 129188, Abu Dhabi, United Arab Emirates; [2]Center for Genomics and Systems Biology, New York University Abu Dhabi (NYUAD), P.O. Box 129188, Abu Dhabi, United Arab Emirates; [3]Science Division, Chemistry Program, New York University Abu Dhabi (NYUAD), P.O. Box 129188, Abu Dhabi, United Arab Emirates; [4]Core Technology Platforms, New York University Abu Dhabi (NYUAD), P.O. Box 129188, Abu Dhabi, United Arab Emirates; [5]Department of Biology, New York University, New York, NY, 10003, USA, [6]Department of Molecular Biosciences, The Wenner-Gren Institute, Stockholm University, SE-106 91 Stockholm, Sweden

* Equal contribution

[6] Correspondence: Piergiorgio Percipalle, address as above

Tel.: +97126284389

Email: pp69@nyu.edu

Running title: β-actin loss dysregulates osteogenic reprogramming

Keywords: β-actin, actin cytoskeleton, osteogenic reprogramming, mineralization, mitochondria

Contents

I.	Experimental procedures	3-6
II.	References	7
III.	Supplementary Figures	8-18
IV.	Supplementary Tables	19-81

I. Experimental procedures

Antibodies and chemicals. Anti-H3K9Me3 antibody (ab8898) is from Abcam. CHIR99021 (SML1046) and Forskolin (F3917) are from Sigma-Aldrich. Anti-Brg1 antibody is from Dr. Anki Östlund-Farrants Lab (Department of Molecular Biosciences, University of Stockholm, Sweden). RevertAid First Strand cDNA Synthesis Kit (K1622) is from Thermo Fisher Scientific. Dexamethasone (1126) is from Tocris Bioscience. Alizarin Red Staining Solution (TMS-008-C) is from EMD Millipore.

Cell culture for MEF. The mouse embryonic fibroblasts (MEFs) WTM, HETM and KOM (from the lab of Dr. Christophe Ampe, University of Gent, Belgium) were maintained and cultured with Dulbecco's modified Eagle medium (DMEM) with high glucose, 10% fetal bovine serum (FBS) and 100 units/mL penicillin and 100 µg/mL streptomycin, in a humidified incubator with 5% CO_2 at 37^0C. Cells were imaged at day 0, 4, 8 and 14 using the EVOS cell imaging system (Thermo Fisher Scientific).

Osteogenic reprogramming protocol with small chemical molecules. OsteoMax-XF differentiation medium (EMD Millipore) was used as the osteogenesis induction medium, with the following supplements: GlutaMAX (1X), penicillin-streptomycin (100 µg/ml), Non-essential Amino Acid (1X) and Trace element B (1X). In addition, small chemical molecules were dissolved and diluted in DMSO and used at the following final concentrations: Dexamethasone (Dex) - 5 mM, Forskolin (F) - 5mM; Chir - 50mM. MEFs were seeded to a Matrigel-coated plate (1:30 dilution in pre-cold PBS, coated overnight at 40^0C, at a density of 10,000 cells/well in a 96-well plate, 60,000 cells/well in a 24-well plate and 500,000 cells/well in a 6-well plate. The MEFs were cultured in DMEM medium until confluent. Once confluent, the DMEM was replaced with an osteo induction medium containing the small molecules; this is considered day 0. The osteo medium was then changed every two days for 14 days.

Alizarin Red staining. Cells in a 96-well-plate were fixed with 3.7% formaldehyde for 5 minutes, washed twice with Milli Q water and incubated with 80µl Alizarin Red stain solution (EMD Millipore) with light shaking at room temperature for 20 minutes. The stain was then removed, cells were washed 3 times with Milli Q water and the wells were imaged with Leica DMI6000 DIC/Fluorescence Inverted Microscope.

Quantitative real time qPCR. Total RNA was extracted using RNeasy Mini Kit (Qiagen) according to the manufacturer's instruction. 200 ng to 1 µg total RNA was reverse transcribed to cDNA using RevertAid First Strand cDNA synthesis Kit (Thermo Fisher Scientific). Diluted cDNA was subjected to quantitative real-time PCR analysis using Maxima SYBR Green qPCR Mix (Thermo Fisher Scientific) on Stratagene 3005 qPCR system (Agilent Technology). All expression levels of target genes were normalized to the expression of Nono reference gene. Data is a summary of 3 biological replicates and 4 technical replicates for each condition. The primers used for qPCR analysis are as follows:

Table S4: Primers for qPCR

**Gene**	**Forward**	**Reverse**
Runx2	TTCAACGATCTGAGATTTGTGGG	GGATGAGGAATGCGCCCTA
Bglap	TCTGCTCACTCTGCTGACCC	CGCCGGAGTCTGTTCACTAC
ALPL	CCAACTCTTTTGTGCCAGAGA	GGCTACATTGGTGTTGAGCTTTT

Spp1	AGCAAGAAACTCTTCCAAGCAA	GTGAGATTCGTCAGATTCATCCG
Fgf23	ATGCTAGGGACCTGCCTTAGA	AGCCAAGCAATGGGGAAGTG
Sost	AGCCTTCAGGAATGATGCCAC	CTTTGGCGTCATAGGGATGGT
DMP1	CACGGACAGCAGTGAATCTGG	GCCGGTCCCCGTACTCTTA

Scanning Electron Microscopy (SEM) and Energy-Dispersive X-ray (EDX) spectroscopy. Cells were washed with PBS, fixed with 3.7% formaldehyde for 5 minutes, and collected by being scraped from the well-plate surface. Upon collection, the cells were centrifuged and the supernatant was discarded. The pellet was then dissolved in ethanol, passed through a 200 nm filter, and eluted with ethanol. 5µl of the sample in ethanol was drop cast onto a metal plate and subjected to SEM analysis. Because of the inorganic nature of the hydroxyapatite crystals, we expected it to not dissolve in ethanol and remain intact for SEM imaging and EDX mapping. The SEM images were obtained using high-vacuum mode on a FEI Quanta 450 Field Emission scanning electron microscope with a primary electron energy of 5–10 kV and spot size of 2.5–3. The EDX experiments were performed by first focusing onto the surface of a single particle and by using an accelerating voltage of 15 keV. The emitted X-rays were monitored until a significant amount of counts were observed (~500 counts/s).

Transmission Electron Microscopy (TEM): sample preparation. 1,000,000 MEF cells were cultured in DMEM medium at a density of 500,000 cells/well in a 6-well plate . Once confluent (70-80%), the DMEM was replaced with an osteo induction medium (OsteoMax + Dex/F). The cells were washed twice with PBS, trypsinized and collected on day 2, and spinned at low speed to form a pellet. High pressure freezing was performed using the Leica ICE high-pressure freezer apparatus with a gold-plated specimen carrier (carrier A, 3mm diameter, 100 µm deep and carrier B with flat side down). Specimen carriers were lightly coated with 0.1 % soy lecithin in chloroform to ensure the opening of the carrier setsand air dried.

Carrier A was filled with pelleted cells and carrier B was placed on top with flat side down before freezing at a programmed pressure of 2100 bars. After freezing, the sample pod was released automatically into a liquid nitrogen bath; the sample carrier was then separated from the specimen pod using precooled fine-tipped tweezers under liquid nitrogen and transferred for Leica freeze substitution AFS2 set up in 2 mL solution of cold dry absolute acetone (v/v) containing 1% osmium tetroxide. The AFS unit was slowly warmed from -90°C to 0°C (2°C/h), with the temperature being held at both -90°C for a period of 15 hrs and thereafter at -60°C and -30°C for a period of 8 h each. Samples were cleared of osmium by rinsing with absolute acetone (3 times × 5 mins) and infiltrated with Epon resin in increasing conc (30%, 66% and 100 %) for 4h each and 100% overnight. Individual samples were embedded in 1 mL for 24-30 h at 60°C. The resin blocks were sectioned using Leica ultramicrotome (UC7) using a diamond knife to get 60nm thick sections and mounted on carbon coated 200 mesh copper grids.

TEM imaging. High resolution transmission electron microscopy (HRTEM) images were obtained using a Talos F200X Scanning/Transmission Electron Microscope with a lattice-fringe resolution of 0.14 nm at an accelerating voltage of 200 kV equipped with a CETA 16M camera. All the relevant areas were marked using bright field imaging mode at spot size 5 and later scanned using the STEM-HDAAF mode. The STEM mode helps in providing the elemental composition as it works on the principle of mass determination. Such measurements can be performed at low electron dose by collecting the high-angle dark-field signal using an annular detector. The annular dark-field signal can also be used to image the elements that have different masses, with the heavier mass element appearing brighter. The STEM

technique has been widely used for visualizing single atoms of high atomic number elements supported on a lower atomic number matrix [1]. STEM imaging of ultrasmall clusters of heavy atoms affords the necessary contrast and Signal to noise ratio for visualization at electron doses that are compatible with biological specimens. In one important application, ultrasmall clusters are used for site-specific labeling of supramolecular assemblies to identify precisely the location of specific subunits within these assemblies [2]. The samples were scanned at spot size 9 and with screen current of 60 pA. The data was analysed using Velox analytical software.

RNA-seq for MEF. The RNA-Seq data for wild-type (WT) β-actin +/+ MEF, heterozygous (HET) β-actin +/- MEF and knock-out (KO) β-actin -/- MEF is previously reported in Xie et. al., 2018, and deposited under the GEO accession number GSE95830[3]. While there were 4 samples for WT, HET and KO MEF cells submitted for RNA-seq analysis, one of the KO samples was of insufficient quality to pass RNA-seq quality control (QC) and was therefore removed from further analysis.

RNA-Seq for reprogrammed osteoblasts: sample prep and sequencing. Three biological replicates of each cell type (WT, HET and KO) were collected on day 4 and 14. Total RNA was extracted and purified using RNeasy Mini Kit (Qiagen, 74106) according to the manufacturer's instruction. RNA-sequencing libraries were constructed using TruSeq RNA Library Prep Kit v2 (Illumina, RS-122-2002) according to the manufacturer's instruction. Briefly, at least 100 ng total RNA was mixed with magnetic Oligo-dT beads to purify the mRNA. The purified mRNA on beads were fragmented and primed. The first strand and second strand cDNA synthesis was performed using SuperScript Double-Stranded cDNA synthesis kit (Invitrogen, 11917020). The synthesized dsDNA was then purified using AMPure XP Beads (Beckman Coulter, A63881). After purification, the DNA sample was end-repaired and adenylated at the 3' end, followed by adaptor ligation. The cDNA was further amplified with index primers using the following protocol: 98^0C for 30 Sec; 15 cycles of: 98^0C for 10 Sec, 60^0C for 30 Sec, and 72^0C for 30 Sec; 72^0C for 5 Min. The PCR product was purified using AMPure XP Beads and the library quality and size was analyzed using 2100 Bioanalyzer (Agilent Genomics). Libraries with compatible index primers were pooled at equal amount and the deep-sequencing was performed using Illumina HiSEq 2500 sequencing platform at New York University Abu Dhabi Sequencing Center.

Statistical Analysis

A. T-tests for pairwise comparisons

Two-sample Welch t-test was performed to compute the significance in pair-wise comparisons on the qPCR data in Figure 1C and Figure S2D. In all cases, significance was defined as $p \leq 0.05$. Statistical analysis was carried out using Prism GraphPad. Matched-pair t-test was performed to compute the significance in pair-wise comparisons on gene expression data for mitochondrial and oxidative phosphorylation genes in Figure 2L-M. In all cases, significance was defined as $p \leq 0.05$. Statistical analysis was carried out using R and associated packages.

B. RNA-seq analysis

Briefly, raw read alignment was performed using tophat2 v2 1.0; the parameters used to specify the genome file were "–no-novel-junctions" and "–G". Mus musculus GRCm38.p4 was used as the reference genome and for GFF annotation. Following the tophat2 alignment, read counts mapped to each gene were generated using HTseq count. Variance stabilizing transformation (VST) was performed to normalize the data using NASQAR[4]. Differential expression (DE) analysis was performed using the DESeq2 R package on all 46,602 genes[5], based on the negative binomial distribution using the following steps: estimation of size factors, estimation of dispersion, negative binomial GLM (general linear model) fitting and Wald statistics. All all DE analysis, significance was defined as Benjamini-Hochberg adjusted p-value < 0.05.

For mitochondria and OXPHOS analyses, genes with p-val adjusted < 0.05 were selected as significant. For OBD, Wnt and Bmp analyses, genes with p-val adjusted < 0.05 and | $\log_2$(fold change) | > 1 were selected as significant. For some analyses, genes were subset based on GO terms: mitochondria (GO: 0005739, n=1845), OXPHOS (GO:0006119, n=108), OBD (GO:0001649, n=203), positive regulation of OBD (GO:0045669, 70 genes), negative regulation of OBD (GO:0045668, n=64), Wnt (GO:0016055, n=444) and BMP signaling pathway (GO:0030509, n=171).

C. Z-score normalization

Z-score normalization was performed in R. First, mean and standard deviation of expression values were computed for every gene. Expression values for each condition and for each gene were normalized using the z-score method to center the data: $z = \frac{x - mean(x)}{s.d.(x)}$, where x is a vector of mean gene expression in all conditions (WTM, WT4, WT14, KOM, KO4, KO14) for a single gene.

D. Violin plots

Violin plots in Figure 2L-M were produced using z-score normalized data of significantly DE mitochondrial genes (n=1136) and oxidative phosphorylation genes (n=68) using R and the ggplot package. Significance of pairwise comparisons between two conditions was determined using two-tailed matched-pairs t-test.

E. Hierarchical clustering

Hierarchical clustering was performed on z-score normalized gene expression data. Agglomerate coefficients were calculated for 4 different linkage methods for each dataset to determine the one that captures the maximum structure of the data (Table S5). For each dataset, the euclidean distance matrix was calculated, Ward.D 2 linkage clustering was computed, and dendrograms were constructed. The number of clusters was determined using Silhouette plots; for most analyses where the optimal value was too low (k < 3), the next best optimal value was used in order to obtain a more granular clustering of the data. Heatmaps were then generated based on hierarchical Ward.D2 clustering and using the sub-optimal number of gene clusters. Statistical analysis was carried out using R and associated packages.

Table S5. Agglomerate coefficients of different linkage methods for each dataset

	OBD	Wnt	Bmp
**average**	0.6449310	0.6915657	0.6065164
**single**	0.4541151	0.5132266	0.4737116
**complete**	0.7259239	0.7584354	0.6810116
**ward**	0.9190068	0.9451137	0.8858413

F. Gene Ontology (GO) enrichment analysis

Differentially expressed genes were selected for enrichment analysis by comparing two different conditions. Gene ontology (GO) analysis was performed using the Web-based DAVID bioinformatics resources [6]. For the GO terms to be considered over-represented or enriched in each gene list, the following criteria was applied: 1- the test p-value is less than 0.05 for the

enrichment, 2- fold of enrichment (observed number of genes in the term/expected number of genes in the term) is ≥ 2 (Table S1D) or ≥ 1.5 (Table S2B-C).

II. Supplementary references

[1] A. V. Crewe, J. Wall, J. Langmore, *Science* **1970**, 168, 1338.

[2] J. F. Hainfeld, R. D. Powell, *Journal of Histochemistry & Cytochemistry* **2000**, 48, 471.

[3] X. Xie, B. Almuzzaini, N. Drou, S. Kremb, A. Yousif, A., A. K. Östlund-Farrants,.,... P. Percipalle, *FASEB J* **2018**, *32*, 1296.

[4] A. Yousif, N. Drou, J. Rowe, M. Khalfan, K. C. Gunsalus, *bioRxiv* **2019**, 709980.

[5] M. I. Love, W. Huber, S. Anders, S, *Genome Biology* **2014**, 15, 550.

[6] D. W. Huang, B. T. Sherman, Q. Tan, J. Kir, D. Liu, D. Bryant, ... R. A. Lempicki, *Nucleic Acids Res.* **2007**, *35*(suppl_2), W169-W175.

III. Supplementary Figures

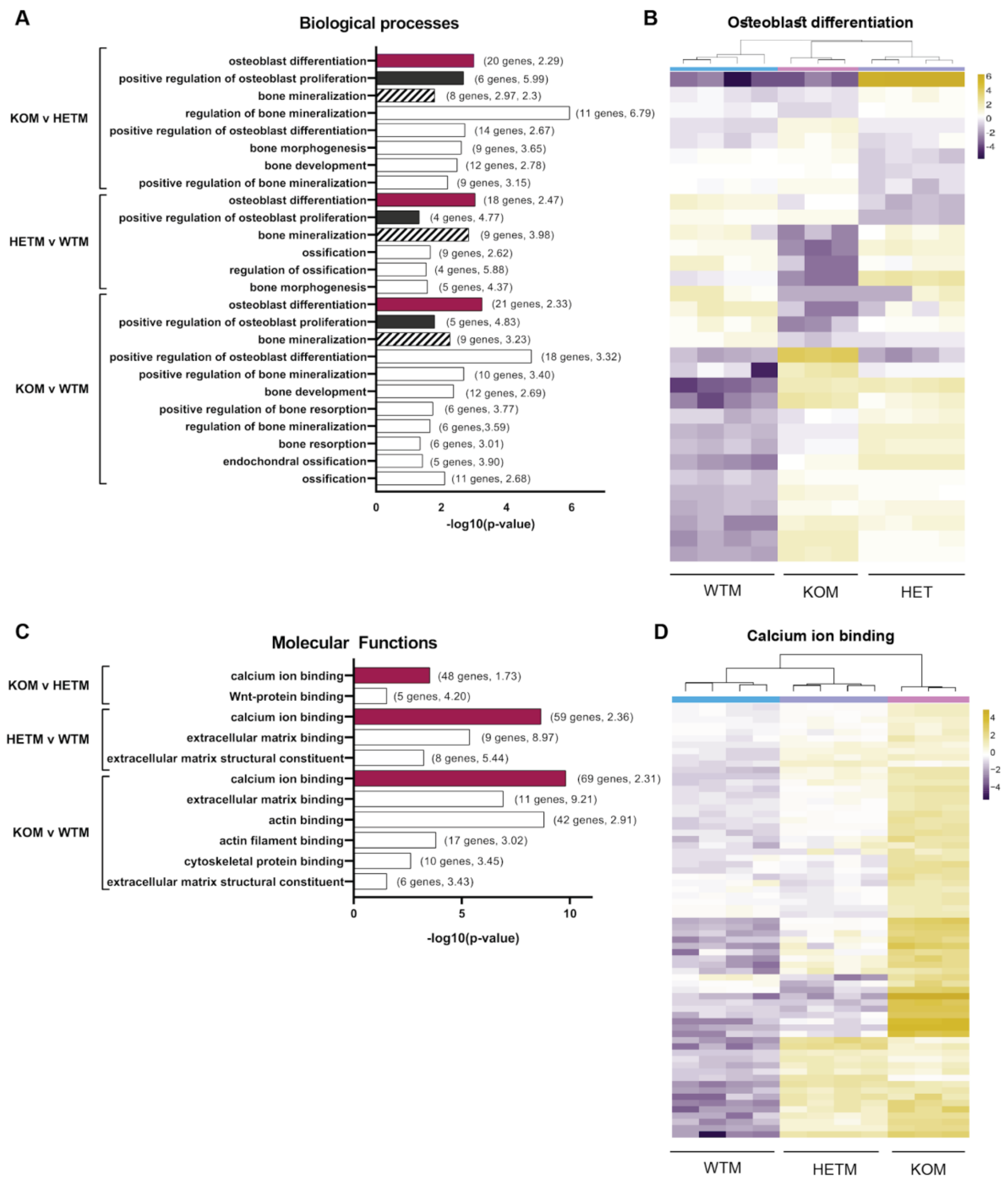

Figure S1. Genes related to osteogenesis and calcium homeostasis are dysregulated in WT, HET and KO MEF cells. (A,C) In each two-way comparison (WTM/HETM, HETM/KOM, WTM/KOM), genes that were differentially expressed (FDR-adjusted p-value < 0.05, |fold change|>2) were subjected to GO enrichment analysis. The statistically significant GO terms (p-value < 0.05, fold of enrichment ≥ 2) are presented for biological processes (A) and molecular function (B). The GO terms shared by all 3 pairwise comparisons are highlighted. **(B,D)** Differentially expressed genes across the three pairwise comparisons for specific GO terms were pooled together. The list of unique genes was subjected to heatmap clustering for osteoblast differentiation (B) and calcium-ion binding (D). Clustering is based on the covariance of gene expression. Scale bar: log_2CPM.

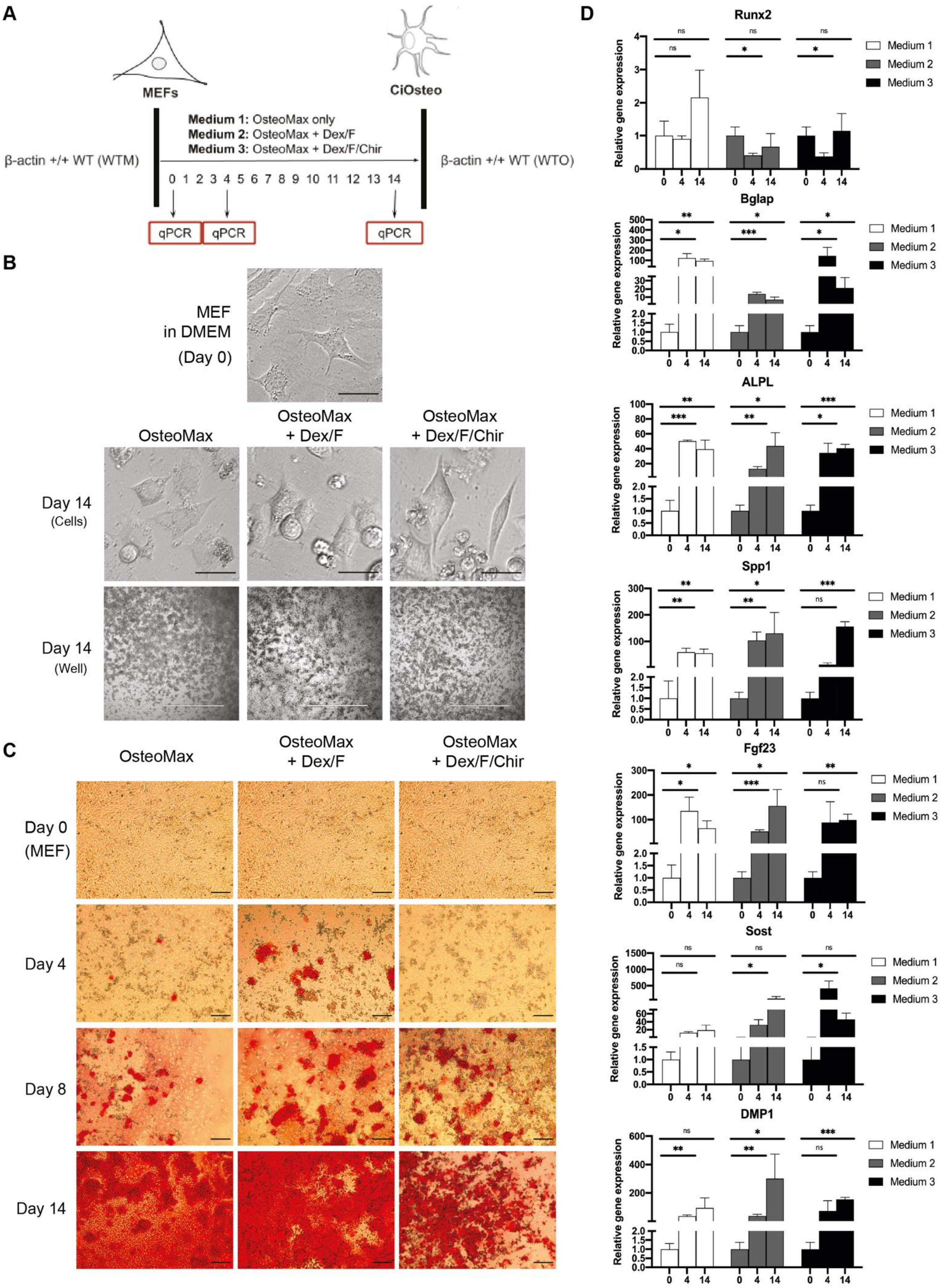
A
MEFs
CiOsteo
Medium 1: OsteoMax only
Medium 2: OsteoMax + Dex/F
Medium 3: OsteoMax + Dex/F/Chir
β-actin +/+ WT (WTM)
β-actin +/+ WT (WTO)
0 1 2 3 4 5 6 7 8 9 10 11 12 13 14
qPCR
qPCR
qPCR
B
MEF
in DMEM
(Day 0)
OsteoMax
OsteoMax
+ Dex/F
OsteoMax
+ Dex/F/Chir
Day 14
(Cells)
Day 14
(Well)
C
OsteoMax
OsteoMax
+ Dex/F
OsteoMax
+ Dex/F/Chir
Day 0
(MEF)
Day 4
Day 8
Day 14
D
Runx2
Bglap
ALPL
Spp1
Fgf23
Sost
DMP1
Relative gene expression
Medium 1
Medium 2
Medium 3
ns
*
**
***

)

Figure S2: Direct reprogramming WT MEF (WTM) to osteoblast-like cells (WTO) with small molecules. (A) Diagram of the experimental setup for direct reprogramming of WTM to WTO using three different mediums composed of OsteoMax medium and cocktails of small molecules: OsteoMAX-XF™ medium only, OsteoMAX-XF™ medium + Dex/F, OsteoMAX-XF™ medium + Dex/F/Chir. Morphology (B), calcium-based mineralization of the ECM by Alizarin Red staining (C) and the expression of osteoblast biomarkers by qPCR (D) was monitored over 14 days. Addition of Chir produced more elongated cells and reduced mineralization at day 14 (Fig S2B-C), and the OsteoMAX-XF™ + Dex/F medium resulted in strongest mineralization at day 14 (Fig S2C). All media induced the RNA expression of bone matrix proteins such as osteocalcin (Bglap) and osteopontin (Spp1), but none induced Runx2 expression (Fig S2D) suggesting possible differences between the small molecules-driven reprogramming and Runx2-dependent osteoblast differentiation in vivo. Notwithstanding, the OsteoMAX-XF™ + Dex/F medium showed effective induction of a functional osteoblastic phenotype with mineralized extracellular matrix at day 14 in WT cells, which was used for all subsequent experiments. **(B)** Cell morphology of WT cells as MEF (in DMEM) and at day 14 (in different reprogramming media). Scale bars: 30µm (row 1 and 2), 100µm (row 3). **(C)** Alizarin Red staining for calcium mineralization in WT cells at day 0 (MEF), 4, 8, and 14. Scale: 100µm. **(D)** Quantification of relative gene expression of key osteogenic biomarkers in MEF. qPCR was performed in MEFs and CiOsteo cells at day 4 and 14, using the three mediums. The *Nono* housekeeping gene was used for normalization, and the expression level of WTM was set to 1 for each gene, and each medium. Data is a summary of 3 biological replicates of reprogrammed cells. Data presented as mean and S.D. P-values are calculated using Welch t-test, *P<0.05, **P<0.01, ***P<0.001, (ns) P>0.5.

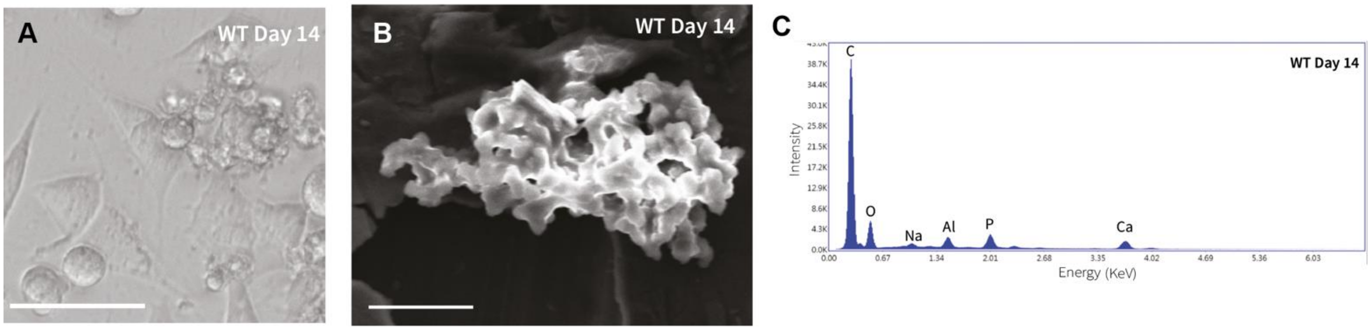

Figure S3. WT cells at day 14 exhibit irregular structures that contain all elements of hydroxyapatite. (A) Microscopy images of alive WT cells at day 14. Scale bar: 50µm **(B)** Scanning Electron Microscopy (SEM) image of the inorganic extracellular components in WT cells at day 14. Scale bar: 10µm **(C)** Energy-dispersive X-ray (EDX) spectra identifies all elements of hydroxyapatite (Ca, C, P and O) on the sample surface in both WT and KO cells. Na, Mg, Al and Si are trace elements from the SEM plate on which the sample was deposited.

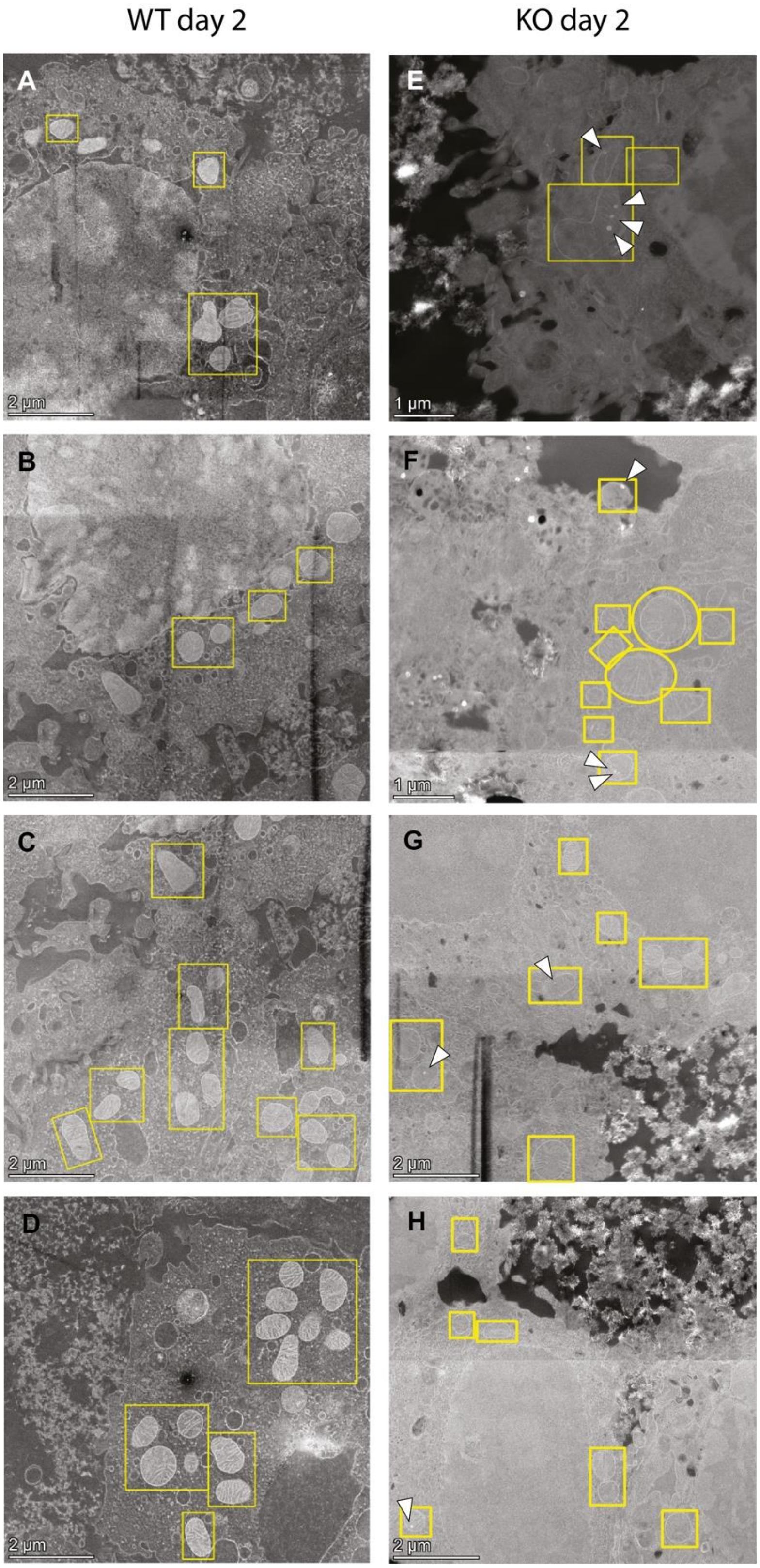

Figure S4. TEM images of mitochondria in WT and KO cells at day 2. (A-D) WT cells at day 2 (WT2) show no intramitochondrial granules. **(E-H)** KO cells at day 2 (KO2) show some intramitochondrial granules (white arrowheads). All mitochondria that were counted for the analysis are highlighted in yellow.

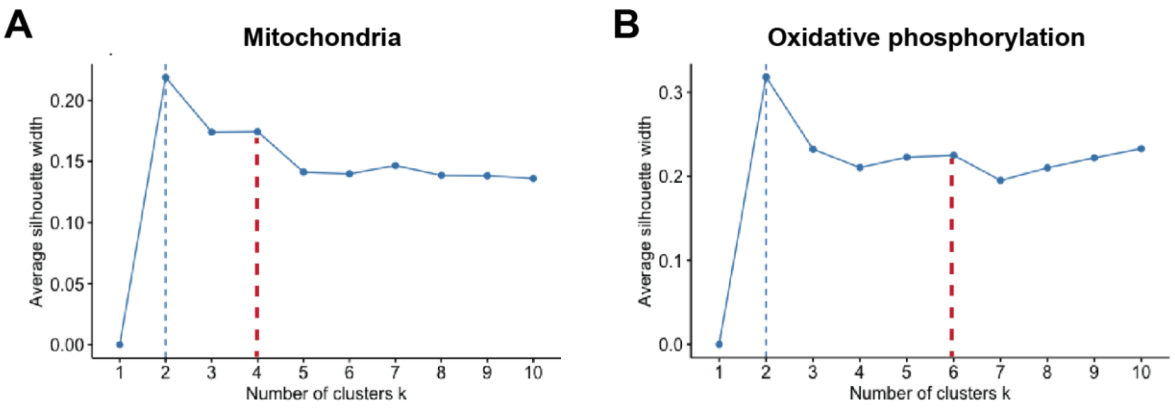

Figure S5. Silhouette plots to determine the optimal number of clusters (k) used in analysis of mitochondria and oxidative phosphorylation (OXPHOS) genes. (A) Optimal k for mitochondria-related genes. K=4 was selected as a sub-optimal cluster to compromise between the determined k=2 optimal clusters and enhancing the granularity of clustering analysis. **(B)** Optimal k for OXPHOS-related genes. K=6 was selected as a sub-optimal cluster to compromise between the determined k=2 optimal clusters and enhancing the granularity of clustering analysis.

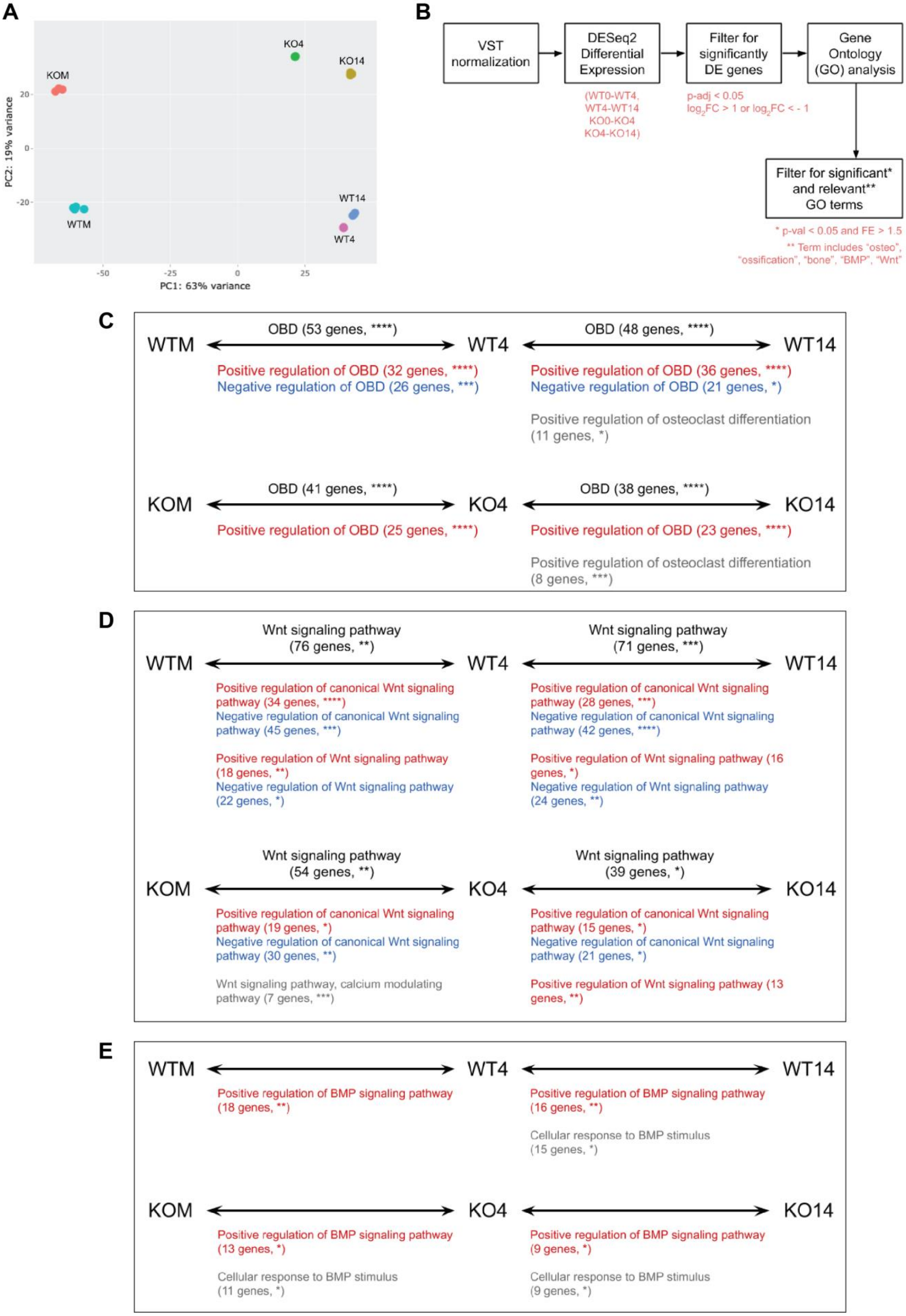
A
KO4
KO14
KOM
WTM
WT14
WT4
PC2: 19% variance
PC1: 63% variance
B
VST normalization
DESeq2 Differential Expression
Filter for significantly DE genes
Gene Ontology (GO) analysis
(WT0-WT4, WT4-WT14 KO0-KO4 KO4-KO14)
p-adj < 0.05
log2FC > 1 or log2FC < - 1
Filter for significant* and relevant** GO terms
* p-val < 0.05 and FE > 1.5
** Term includes "osteo", "ossification", "bone", "BMP", "Wnt"
C
WTM
OBD (53 genes, ****)
WT4
OBD (48 genes, ****)
WT14
Positive regulation of OBD (32 genes, ****)
Negative regulation of OBD (26 genes, ***)
Positive regulation of OBD (36 genes, ****)
Negative regulation of OBD (21 genes, *)
Positive regulation of osteoclast differentiation (11 genes, *)
KOM
OBD (41 genes, ****)
KO4
OBD (38 genes, ****)
KO14
Positive regulation of OBD (25 genes, ****)
Positive regulation of OBD (23 genes, ****)
Positive regulation of osteoclast differentiation (8 genes, ***)
D
WTM
Wnt signaling pathway (76 genes, **)
WT4
Wnt signaling pathway (71 genes, ***)
WT14
Positive regulation of canonical Wnt signaling pathway (34 genes, ****)
Negative regulation of canonical Wnt signaling pathway (45 genes, ***)
Positive regulation of Wnt signaling pathway (18 genes, **)
Negative regulation of Wnt signaling pathway (22 genes, *)
Positive regulation of canonical Wnt signaling pathway (28 genes, ***)
Negative regulation of canonical Wnt signaling pathway (42 genes, ****)
Positive regulation of Wnt signaling pathway (16 genes, *)
Negative regulation of Wnt signaling pathway (24 genes, **)
KOM
Wnt signaling pathway (54 genes, **)
KO4
Wnt signaling pathway (39 genes, *)
KO14
Positive regulation of canonical Wnt signaling pathway (19 genes, *)
Negative regulation of canonical Wnt signaling pathway (30 genes, **)
Wnt signaling pathway, calcium modulating pathway (7 genes, ***)
Positive regulation of canonical Wnt signaling pathway (15 genes, *)
Negative regulation of canonical Wnt signaling pathway (21 genes, *)
Positive regulation of Wnt signaling pathway (13 genes, **)
E
WTM
WT4
WT14
Positive regulation of BMP signaling pathway (18 genes, **)
Positive regulation of BMP signaling pathway (16 genes, **)
Cellular response to BMP stimulus (15 genes, *)
KOM
KO4
KO14
Positive regulation of BMP signaling pathway (13 genes, *)
Cellular response to BMP stimulus (11 genes, *)
Positive regulation of BMP signaling pathway (9 genes, *)
Cellular response to BMP stimulus (9 genes, *)

Figure S6. Exploratory RNA-seq DE analysis on osteogenesis-related genes. (A) Principal Component Analysis (PCA) shows clear separation of MEFs and osteoblast-like cells (PC1, 63% variance), as well as of WT and KO (PC2, 19% variance), resulting in 6 distinct clusters for the 6 conditions. **(B)** Analytical pipeline for RNA-seq data normalization, differential expression and gene ontology (GO) enrichment analysis. Summary of all resulting data is provided in Table S2B-C). **(C-E)** Summary of GO analysis focusing on osteoblast-differentiation (OBD)-related genes (C), Wnt signaling-related genes (D) and BMP signaling-related genes (E), including the parent GO term (black), positive regulation GO terms (red), negative regulation GO terms (blue) and other relevant GO terms (grey). Numbers in parentheses reflect number of genes, and significance of GO term: *P<0.05, **P<0.01, ***P<0.001, ****P<0.0001, (ns) P>0.05.

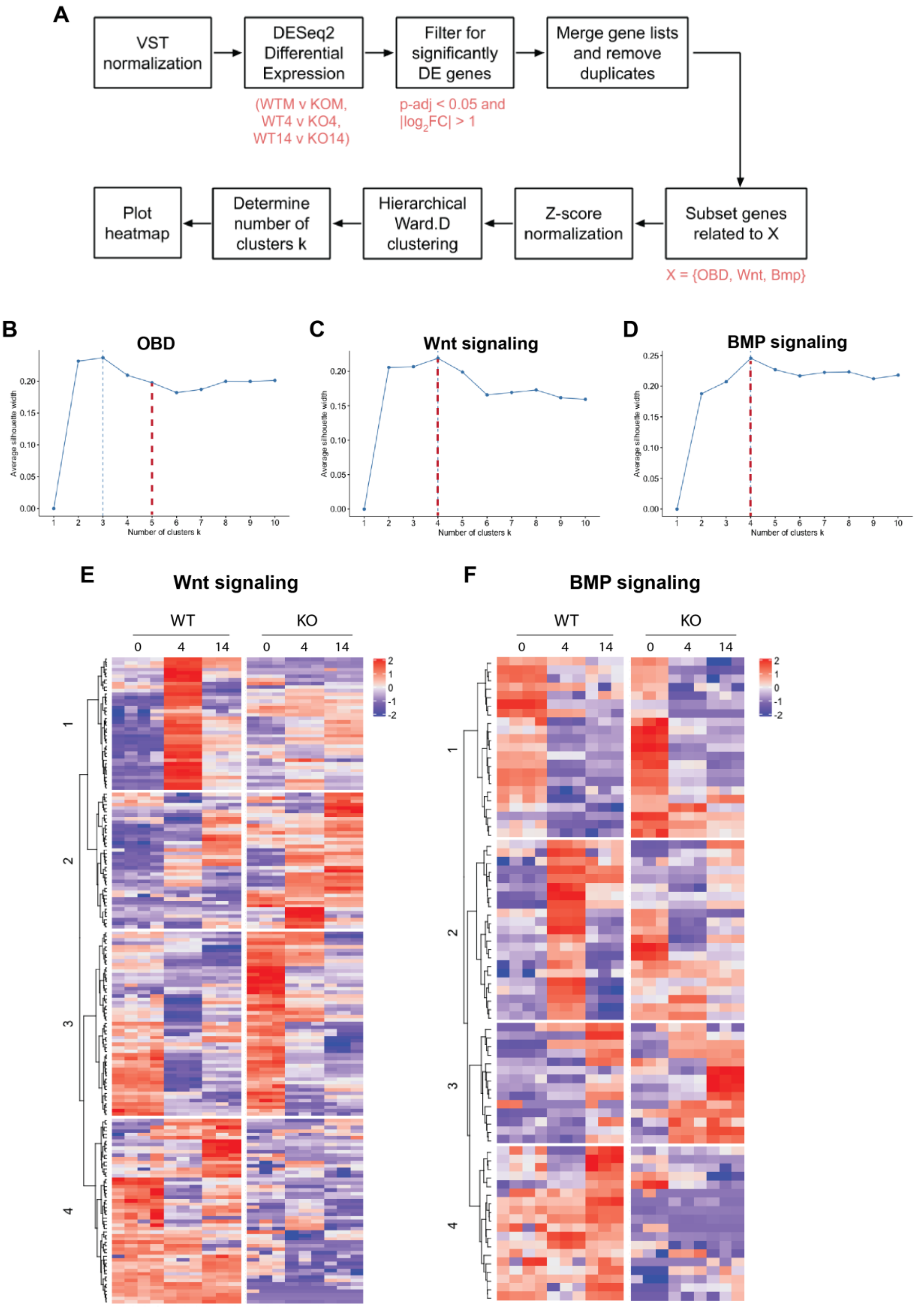

Figure S7. OBD, Wnt and Bmp signaling pathways in reprogrammed WT and KO cells. (A) Analytical pipeline for RNA-seq normalization, DESeq2 DE, Z-score normalization and hierarchical

clustering. **(B-D)** Silhouette plots for determining optimal and sub-optimal number of clusters (k) for OBD (B), Wnt (C) and BMP-related genes (D). The sub-optimal cluster number was selected for each dataset (k=5, k=4 and k=4, for OBD, Wnt and BMP, respectively). **(E-F)** Heatmaps of genes that are significantly DE (p-val < 0.05, and |fold change| > 2) in at least one pair-wise comparison (WTM v KOM, WT4 v KO4, WT14 v KO14) that belong to the Wnt or BMP GO terms. Heatmap shows z-score normalized expression data and reveals distinctive temporal patterns of expression in WT and KO cells at days 0, 4 and 14. Hierarchical clustering was done using Ward.D linkage; k=4 for both Wnt and BMP signaling heatmaps was determined per above.

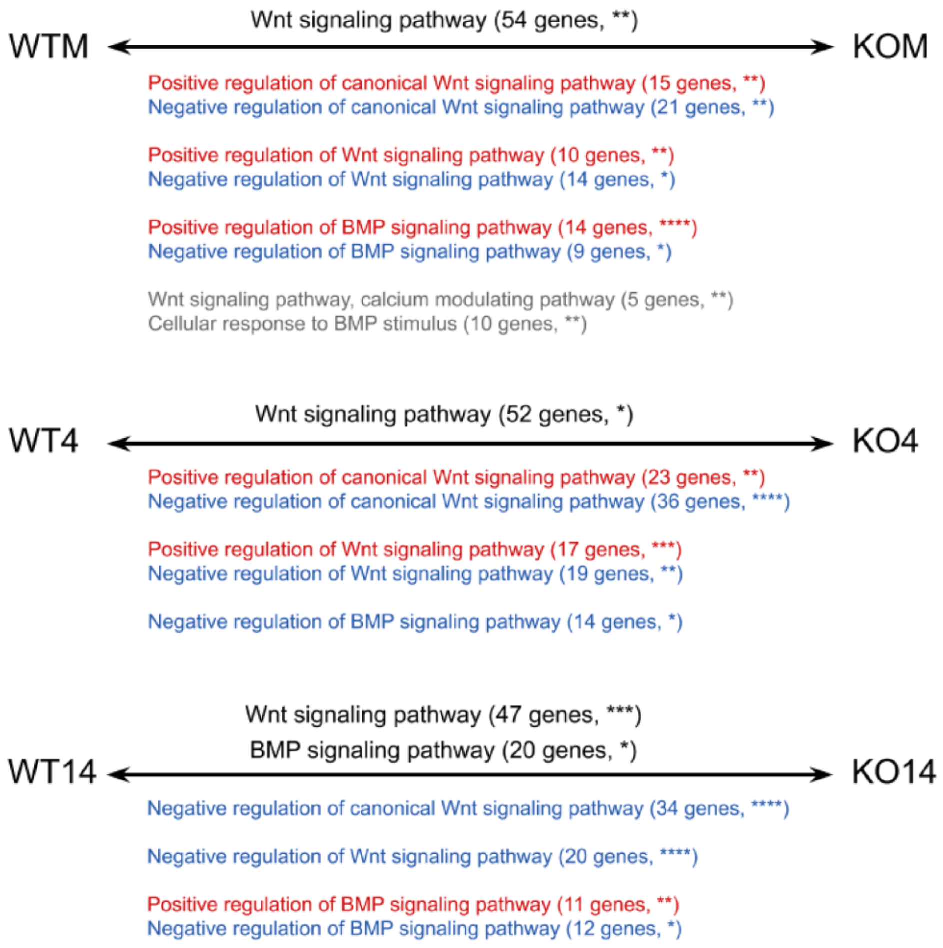

Figure S8. Analysis of differential expression of Wnt and BMP signaling pathways between WT and KO cells. Summary of GO analysis on DE genes (p-adjusted < 0.05, |fold change| > 2) between WT and KO at different timepoints: WTM v KOM, WT4 v KO4, WT14 v KO14, including parent GO term (black), positive regulation GO terms (red), negative regulation GO terms (blue) and other relevant GO terms (grey). Numbers in parentheses reflect number of genes, and significance of GO term: *P<0.05, **P<0.01, ***P<0.001, ****P<0.0001, (ns) P>0.05.

IV. Supplementary Tables

Table S1. Mitochondria and OXPHOS genes used in analyses

A. 1136 mitochondria-related genes that are DE (p-adj < 0.05) between WT4 and KO4 cells.

gene.ids	gene.name	WT4 vs KO4		Cluster
		log2FC	p-adj	
ENSMUSG00000000171	Sdhd	-0.1983493	0.00195243	2
ENSMUSG00000000295	Hddc2	0.36952854	0.00114912	4
ENSMUSG00000000301	Pemt	-0.4572993	0.00428545	2
ENSMUSG00000000326	Comt	0.66418292	3.26E-11	3
ENSMUSG00000000340	Dbt	-0.7050178	3.06E-25	2
ENSMUSG00000000384	Tbrg4	-0.6050688	5.43E-07	1
ENSMUSG00000000399	Ndufa9	0.41963685	3.16E-14	4
ENSMUSG00000000441	Raf1	0.45012042	5.80E-08	4
ENSMUSG00000000594	Gm2a	1.977227	1.45E-98	4
ENSMUSG00000000628	Hk2	-2.1236559	1.68E-288	1
ENSMUSG00000000738	Spg7	1.12986037	8.70E-21	4
ENSMUSG00000000811	Txnrd3	-0.6824555	5.84E-17	1
ENSMUSG00000001127	Araf	0.93400488	1.07E-54	4
ENSMUSG00000001156	Mxd1	-1.137696	2.55E-62	1
ENSMUSG00000001175	Calm1	1.02729624	2.98E-64	4
ENSMUSG00000001285	Myg1	-0.468244	1.54E-07	1
ENSMUSG00000001380	Hars	-0.2932876	3.37E-08	2
ENSMUSG00000001424	Snd1	-0.2974797	2.10E-16	1
ENSMUSG00000001445	Mrpl10	0.26576551	4.95E-05	3
ENSMUSG00000001755	Coasy	-0.4279981	0.0111228	1

ENSMUSG00000001786	Fbxo7	-0.4109019	6.84E-05	2
ENSMUSG00000001829	Clpb	-0.3653233	7.41E-08	1
ENSMUSG00000001870	Ltbp1	-0.6048225	1.08E-22	2
ENSMUSG00000002010	Idh3g	-0.3122985	1.62E-09	1
ENSMUSG00000002083	Bbc3	-0.9218587	2.30E-05	1
ENSMUSG00000002332	Dhrs1	0.39347668	5.95E-09	4
ENSMUSG00000002346	Slc25a42	1.58924876	3.39E-31	4
ENSMUSG00000002416	Ndufb2	0.75538681	1.44E-13	4
ENSMUSG00000002661	Alkbh7	0.78234443	8.87E-07	3
ENSMUSG00000002741	Ykt6	0.27895119	2.91E-06	4
ENSMUSG00000002814	Top3a	-0.3270183	2.80E-07	2
ENSMUSG00000002825	Qtrt1	-1.605452	1.50E-24	1
ENSMUSG00000002846	Timmdc1	-0.1927766	0.0043757	1
ENSMUSG00000002949	Timm44	-0.2400197	0.00410579	1
ENSMUSG00000002984	Tomm40	-0.9090189	3.93E-05	1
ENSMUSG00000003161	Sri	0.74578123	2.28E-39	4
ENSMUSG00000003226	Ranbp2	-0.7728586	7.07E-12	2
ENSMUSG00000003438	Timm50	-0.5701331	0.00082936	1
ENSMUSG00000003458	Ncstn	0.2233218	1.61E-05	4
ENSMUSG00000003528	Slc25a1	0.76593556	1.30E-49	3
ENSMUSG00000003623	Crot	1.09553091	4.77E-37	4
ENSMUSG00000003762	Adck4	-0.4523151	0.00031491	1
ENSMUSG00000003809	Gcdh	1.16501713	2.66E-68	4
ENSMUSG00000003923	Tfam	-1.0706438	2.13E-34	2
ENSMUSG00000004040	Stat3	-0.1615788	0.02197879	3
ENSMUSG00000004044	Ptrf	0.70766329	3.47E-29	4

ENSMUSG00000004069	Dnaja3	-0.4770343	8.51E-36	1
ENSMUSG00000004127	Trmt10a	-0.7475608	2.06E-23	2
ENSMUSG00000004207	Psap	0.19458147	0.00503352	3
ENSMUSG00000004233	Wars2	-0.2699839	0.0263952	2
ENSMUSG00000004264	Phb2	-0.5087753	1.88E-13	1
ENSMUSG00000004446	Bid	0.27668073	2.37E-07	4
ENSMUSG00000004637	Wwox	0.45126552	2.08E-06	3
ENSMUSG00000004748	Mtfp1	-1.9705039	3.25E-14	1
ENSMUSG00000004771	Rab11a	-0.4515813	2.37E-10	2
ENSMUSG00000004789	Dlst	-0.2400918	1.43E-16	2
ENSMUSG00000004929	Thop1	-0.9557902	8.47E-06	1
ENSMUSG00000004936	Map2k1	-0.4561838	2.24E-49	1
ENSMUSG00000005161	Prdx2	0.92354498	7.88E-21	4
ENSMUSG00000005360	Slc1a3	1.41835219	6.03E-232	3
ENSMUSG00000005469	Prkaca	1.09138121	6.15E-132	4
ENSMUSG00000005510	Ndufs3	-0.4340482	1.52E-09	2
ENSMUSG00000005667	Mthfd2	-1.260524	3.63E-109	1
ENSMUSG00000005674	Tomm40l	0.76441367	1.11E-07	3
ENSMUSG00000005683	Cs	0.28679068	7.48E-09	4
ENSMUSG00000005718	Tfap4	-0.5745555	4.63E-08	1
ENSMUSG00000005981	Trap1	0.54594886	9.12E-37	4
ENSMUSG00000006057	Atp5g1	0.50233053	0.00031751	4
ENSMUSG00000006221	Hspb7	3.38208228	9.15E-35	3
ENSMUSG00000006378	Gcat	1.52811759	3.39E-14	3
ENSMUSG00000006494	Pdk1	0.78613733	0.00015261	4
ENSMUSG00000006519	Cyba	-0.966258	0.00441878	2

ENSMUSG00000006717	Acot13	0.61803162	5.22E-25	3
ENSMUSG00000006724	Cyp27b1	-1.7059961	3.65E-05	1
ENSMUSG00000006782	Cnp	0.52806961	0.00051637	4
ENSMUSG00000006818	Sod2	-0.4440933	8.50E-26	1
ENSMUSG00000007041	Clic1	0.34593334	4.79E-07	4
ENSMUSG00000007655	Cav1	2.05615561	6.87E-141	4
ENSMUSG00000008398	Elk3	0.40890912	1.32E-09	4
ENSMUSG00000008435	Rdh13	-0.933634	2.10E-78	1
ENSMUSG00000008540	Mgst1	1.57910579	2.96E-142	3
ENSMUSG00000008683	Rps15a	-0.4633096	0.00021783	1
ENSMUSG00000008892	Vdac3	-0.3114396	0.00930132	2
ENSMUSG00000009013	Dynll1	0.33945189	5.05E-07	4
ENSMUSG00000009112	Bcl2l13	-0.658638	1.36E-37	2
ENSMUSG00000009406	Elk1	0.58672177	6.78E-19	4
ENSMUSG00000009614	Sardh	1.54773329	1.33E-62	3
ENSMUSG00000009647	Mcu	-0.2603281	3.42E-05	3
ENSMUSG00000009863	Sdhb	0.46184137	8.46E-08	3
ENSMUSG00000010054	Tusc2	-0.2378679	0.00242293	2
ENSMUSG00000010609	Psen2	0.79650237	1.06E-20	3
ENSMUSG00000010651	Acaa1b	1.58893587	5.43E-05	3
ENSMUSG00000010663	Fads1	-0.2687096	2.04E-08	1
ENSMUSG00000011254	Thg1l	-0.605168	5.32E-16	2
ENSMUSG00000013539	Tango2	1.30406745	7.60E-50	4
ENSMUSG00000013593	Ndufs2	0.58420549	6.02E-30	4
ENSMUSG00000013662	Atad1	-0.6586724	4.41E-16	1
ENSMUSG00000013701	Timm23	-0.8462334	2.22E-16	2

ENSMUSG00000013736	Trnt1	-0.9235302	4.06E-20	2
ENSMUSG00000013997	Nit1	0.26612206	0.0149282	4
ENSMUSG00000014313	Cox6c	0.85927017	1.34E-23	3
ENSMUSG00000014554	Dguok	0.6293764	3.18E-17	3
ENSMUSG00000014606	Slc25a11	0.39659568	2.64E-09	4
ENSMUSG00000014633	Cmc2	1.02513972	4.19E-21	3
ENSMUSG00000015016	Acsf3	1.13020451	5.96E-14	4
ENSMUSG00000015112	Slc25a13	1.17010639	1.22E-67	4
ENSMUSG00000015133	Lrrk1	0.81544295	4.38E-16	4
ENSMUSG00000015247	Nipsnap3b	0.2825061	4.04E-07	4
ENSMUSG00000015340	Cybb	-4.7006743	0.02403871	1
ENSMUSG00000015478	Rnf5	0.28656395	0.02490384	3
ENSMUSG00000015672	Mrpl32	-0.3702275	9.54E-06	2
ENSMUSG00000015750	Aph1a	0.51479777	0.00182349	3
ENSMUSG00000015790	Surf1	0.50405635	1.42E-13	3
ENSMUSG00000015806	Qdpr	0.89562041	1.08E-41	4
ENSMUSG00000015837	Sqstm1	-1.6335082	7.57E-109	1
ENSMUSG00000015961	Adss	-0.2370694	2.11E-05	2
ENSMUSG00000016128	Stard13	-0.5282695	2.18E-09	2
ENSMUSG00000016252	Atp5e	0.67501513	9.92E-09	3
ENSMUSG00000016257	Slmo2	-0.2736215	0.00013005	1
ENSMUSG00000016427	Ndufa1	1.03490755	2.55E-17	3
ENSMUSG00000016495	Plgrkt	1.30672282	2.08E-43	4
ENSMUSG00000016758	Bik	3.63195877	2.67E-10	4
ENSMUSG00000017119	Nbr1	-0.7553217	2.43E-41	1
ENSMUSG00000017146	Brca1	-0.6700755	1.20E-14	2

ENSMUSG00000017286	Glod4	0.49916957	5.20E-09	4
ENSMUSG00000018042	Cyb5r3	0.71732813	1.55E-12	4
ENSMUSG00000018167	Stard3	-0.5127854	9.14E-14	1
ENSMUSG00000018196	Glrx2	0.51017086	3.35E-07	4
ENSMUSG00000018322	Tomm34	-0.3617185	2.50E-08	2
ENSMUSG00000018405	Mrm1	-0.3016946	0.00716844	1
ENSMUSG00000018446	C1qbp	-1.0744042	2.57E-42	1
ENSMUSG00000018574	Acadvl	0.45292622	1.63E-22	4
ENSMUSG00000018599	Mief2	0.81909839	7.28E-06	3
ENSMUSG00000018740	Slc25a35	2.63008817	3.93E-12	4
ENSMUSG00000018796	Acsl1	1.22461241	1.96E-60	4
ENSMUSG00000018848	Rars	-0.6179214	3.07E-11	1
ENSMUSG00000018861	Fdxr	1.05779012	7.62E-05	3
ENSMUSG00000018882	Mrpl45	-0.2209468	0.00039785	2
ENSMUSG00000018995	Nars2	-0.9987997	7.36E-31	1
ENSMUSG00000019054	Fis1	0.73956364	5.11E-10	4
ENSMUSG00000019066	Rab3d	2.40893512	4.78E-152	4
ENSMUSG00000019082	Slc25a22	-0.7903031	0.0002029	1
ENSMUSG00000019143	Hars2	0.30519075	8.66E-05	4
ENSMUSG00000019210	Atp6v1e1	-0.2921742	4.22E-08	1
ENSMUSG00000019505	Ubb	-0.8411135	7.71E-27	1
ENSMUSG00000019689	1110001J03Rik	0.65404457	0.0008853	3
ENSMUSG00000019710	Mrpl24	-0.305297	0.00887959	3
ENSMUSG00000019763	Rmnd1	0.67000945	8.14E-41	4
ENSMUSG00000019774	Mtrf1l	-0.8534432	6.81E-16	1
ENSMUSG00000019831	Wasf1	-0.2303579	0.00479566	2

ENSMUSG00000019843	Fyn	-0.5384898	1.01E-25	1
ENSMUSG00000019851	Perp	3.05829704	1.79E-167	4
ENSMUSG00000019853	Hebp2	0.37470337	8.99E-05	4
ENSMUSG00000019863	Qrsl1	0.48345259	1.87E-08	4
ENSMUSG00000019864	Rtn4ip1	0.5484217	5.59E-07	3
ENSMUSG00000019916	P4ha1	-0.1878388	0.01777168	2
ENSMUSG00000019969	Psen1	-0.5764204	2.18E-19	2
ENSMUSG00000019970	Sgk1	-1.1143731	0	1
ENSMUSG00000020022	Ndufa12	-0.3629585	5.71E-05	3
ENSMUSG00000020038	Cry1	-1.1380634	1.84E-89	1
ENSMUSG00000020063	Sirt1	-1.4117697	5.05E-67	2
ENSMUSG00000020075	Ddx21	-1.5099482	1.79E-189	1
ENSMUSG00000020079	Supv3l1	-0.5422684	7.55E-20	2
ENSMUSG00000020085	Aifm2	-0.9070637	3.04E-69	1
ENSMUSG00000020108	Ddit4	-0.5038642	5.95E-30	1
ENSMUSG00000020130	Tbc1d15	-1.3181448	1.20E-79	1
ENSMUSG00000020250	Txnrd1	-0.858475	4.69E-198	1
ENSMUSG00000020253	Ppm1m	0.57094956	1.67E-13	3
ENSMUSG00000020256	Aldh1l2	-0.7364757	5.83E-08	1
ENSMUSG00000020268	Lyrm7	0.76104534	0.00972685	4
ENSMUSG00000020284	1810043G02Rik	0.92740499	1.98E-06	3
ENSMUSG00000020321	Mdh1	0.75759516	8.67E-22	4
ENSMUSG00000020333	Acsl6	-1.499116	4.29E-25	2
ENSMUSG00000020334	Slc22a4	1.02643281	1.36E-09	3
ENSMUSG00000020359	Phykpl	-0.3975433	0.02056776	2
ENSMUSG00000020366	Mapk9	0.84868109	1.40E-22	4

ENSMUSG00000020372	Gnb2l1	-0.5723422	4.38E-08	1
ENSMUSG00000020402	Vdac1	0.32608001	8.50E-10	4
ENSMUSG00000020456	Ogdh	0.48355947	2.41E-33	4
ENSMUSG00000020477	Mrps24	1.05958078	5.00E-18	3
ENSMUSG00000020514	Mrpl22	-0.3338096	9.41E-07	2
ENSMUSG00000020527	Myo19	-0.7702848	1.17E-11	2
ENSMUSG00000020532	Acaca	-0.2405592	0.01221636	2
ENSMUSG00000020534	Shmt1	-1.2423428	1.34E-126	1
ENSMUSG00000020537	Drg2	-0.6360446	2.12E-22	2
ENSMUSG00000020549	Elac2	-0.3089502	6.24E-09	2
ENSMUSG00000020593	Lpin1	2.41565415	8.48E-146	3
ENSMUSG00000020605	Hs1bp3	0.4966435	3.20E-11	4
ENSMUSG00000020620	Abca8b	1.00868793	1.67E-08	3
ENSMUSG00000020621	Rdh14	0.29821802	0.02591736	3
ENSMUSG00000020630	Rnaseh1	-0.4879387	0.00084541	1
ENSMUSG00000020638	Cmpk2	-8.8020177	2.81E-12	2
ENSMUSG00000020641	Rsad2	-11.1257	2.56E-26	1
ENSMUSG00000020657	Dnajc27	0.36796276	3.13E-06	3
ENSMUSG00000020664	Dld	-0.3576807	5.05E-07	2
ENSMUSG00000020697	Lig3	-0.4521462	5.51E-06	2
ENSMUSG00000020715	Ern1	-1.4817952	0	1
ENSMUSG00000020736	Nt5c	0.58074721	2.79E-06	3
ENSMUSG00000020775	Mrpl38	-0.2704786	0.00439958	1
ENSMUSG00000020777	Acox1	0.36124889	5.68E-26	3
ENSMUSG00000020843	Timm22	0.24671441	0.00721257	4
ENSMUSG00000020849	Ywhae	-0.2246124	0.00137784	2

ENSMUSG00000020857	Nme2	-0.3307009	0.00239469	1
ENSMUSG00000020869	Lrrc59	-0.4488146	8.91E-26	1
ENSMUSG00000020917	Acly	0.84818333	1.18E-74	3
ENSMUSG00000021023	1110008L16Rik	-0.298754	0.01511194	2
ENSMUSG00000021033	Gstz1	0.97888279	7.99E-29	4
ENSMUSG00000021036	Sptlc2	-0.4384034	5.34E-11	1
ENSMUSG00000021040	Slirp	0.74484336	1.99E-07	4
ENSMUSG00000021044	Adck1	1.89728594	4.95E-104	3
ENSMUSG00000021054	Sgpp1	0.24445673	0.00062883	4
ENSMUSG00000021079	Timm9	-0.5519143	3.85E-22	1
ENSMUSG00000021125	Arg2	0.4887855	5.06E-13	3
ENSMUSG00000021185	9030617O03Rik	5.04215367	6.18E-47	4
ENSMUSG00000021189	Atxn3	-0.5573773	7.16E-08	1
ENSMUSG00000021217	Tshz3	-0.6645825	6.21E-09	1
ENSMUSG00000021226	Acot2	-2.4151501	3.89E-165	1
ENSMUSG00000021235	Coq6	0.77716783	2.90E-10	4
ENSMUSG00000021238	Aldh6a1	1.81302429	1.61E-59	4
ENSMUSG00000021241	Isca2	0.50504953	3.12E-10	3
ENSMUSG00000021287	Xrcc3	0.307106	0.01820006	4
ENSMUSG00000021290	2010107E04Rik	0.73616145	3.71E-08	4
ENSMUSG00000021302	Ggps1	-0.4011364	0.00103605	2
ENSMUSG00000021339	Mrs2	0.37898117	0.00043448	4
ENSMUSG00000021361	Tmem14c	0.64740793	7.52E-20	3
ENSMUSG00000021371	Mcur1	-0.694783	4.48E-18	2
ENSMUSG00000021408	Ripk1	-0.3304534	0.02179527	2
ENSMUSG00000021417	Eci2	0.60467733	1.85E-26	4

ENSMUSG00000021420	Fars2	0.36728507	8.90E-05	3
ENSMUSG00000021432	Slc35b3	-0.5370141	2.98E-11	2
ENSMUSG00000021470	Ercc6l2	-0.9269518	1.06E-23	2
ENSMUSG00000021474	Sfxn1	-0.1899539	0.01660752	2
ENSMUSG00000021496	Pcbd2	1.22796966	1.82E-20	3
ENSMUSG00000021519	Mterf3	-0.7496545	5.09E-14	2
ENSMUSG00000021520	Uqcrb	0.3221319	0.00234061	4
ENSMUSG00000021532	Fastkd3	-1.1185594	1.86E-29	2
ENSMUSG00000021546	Hnrnpk	-0.4292819	1.34E-16	2
ENSMUSG00000021577	Sdha	0.20066657	0.00065839	4
ENSMUSG00000021591	Glrx	-0.8623132	2.22E-41	1
ENSMUSG00000021595	Nsun2	-1.3436835	2.94E-165	2
ENSMUSG00000021607	Mrpl36	-0.4391408	5.03E-07	1
ENSMUSG00000021611	Tert	-8.3145851	5.35E-11	2
ENSMUSG00000021646	Mccc2	1.0094001	6.01E-28	4
ENSMUSG00000021650	Ptcd2	-0.2601994	0.00027206	2
ENSMUSG00000021669	Col4a3bp	-0.9028557	1.02E-20	2
ENSMUSG00000021692	Dimt1	-1.4373814	1.23E-76	1
ENSMUSG00000021707	Dhfr	-1.889766	1.69E-125	1
ENSMUSG00000021731	Mrps30	-1.2142072	4.09E-43	2
ENSMUSG00000021748	Pdhb	-0.4563959	6.19E-19	1
ENSMUSG00000021764	Ndufs4	0.35602557	0.00087232	4
ENSMUSG00000021792	Fam213a	-3.7298526	3.40E-120	1
ENSMUSG00000021809	Nudt13	0.57290316	0.00160491	4
ENSMUSG00000021868	Ppif	-0.8059533	1.84E-30	1
ENSMUSG00000021892	Sh3bp5	1.11500232	2.06E-40	4

ENSMUSG00000021906	Oxnad1	-1.1251173	7.19E-42	2
ENSMUSG00000021913	Ogdhl	0.73119804	1.46E-07	3
ENSMUSG00000021936	Mapk8	-0.6695912	2.89E-14	2
ENSMUSG00000021939	Ctsb	-0.4157566	4.22E-22	1
ENSMUSG00000021973	Micu2	0.56955273	2.31E-08	4
ENSMUSG00000021993	Mipep	0.51920208	2.37E-06	2
ENSMUSG00000022003	Slc25a30	-0.6885922	1.03E-19	1
ENSMUSG00000022013	Dnajc15	0.25785153	0.04725355	4
ENSMUSG00000022022	Mtrf1	-4.9707051	1.05E-31	2
ENSMUSG00000022037	Clu	-3.5900259	3.43E-50	3
ENSMUSG00000022048	Dpysl2	1.1443579	5.11E-60	4
ENSMUSG00000022051	Bnip3l	-0.32087	2.77E-07	2
ENSMUSG00000022110	Sucla2	0.27892309	0.00479767	4
ENSMUSG00000022126	Irg1	-7.2535082	1.59E-08	1
ENSMUSG00000022186	Oxct1	0.50656236	5.99E-16	4
ENSMUSG00000022200	Golph3	-0.6662561	2.01E-17	2
ENSMUSG00000022204	Ngdn	-0.8061057	5.22E-19	2
ENSMUSG00000022210	Dhrs4	-0.3342065	0.0040372	2
ENSMUSG00000022221	Ripk3	-0.7532068	7.52E-14	2
ENSMUSG00000022244	Amacr	0.44265883	0.00020077	3
ENSMUSG00000022246	Rai14	-0.9901942	7.96E-62	1
ENSMUSG00000022253	Nadk2	0.86978432	1.75E-58	3
ENSMUSG00000022323	Hrsp12	1.67454458	1.88E-78	4
ENSMUSG00000022337	Emc2	1.18003289	1.06E-42	4
ENSMUSG00000022354	Ndufb9	0.6491557	1.36E-14	3
ENSMUSG00000022386	Trmu	0.30110021	0.00166915	3

ENSMUSG00000022401	Xpnpep3	-0.2822907	0.00047438	2
ENSMUSG00000022404	Slc25a17	0.13353876	0.04687278	4
ENSMUSG00000022407	Adsl	-0.5241246	8.76E-08	1
ENSMUSG00000022412	Mief1	-0.2709653	5.64E-09	1
ENSMUSG00000022427	Tomm22	0.23000931	0.0006775	4
ENSMUSG00000022437	Samm50	0.38481923	2.65E-10	3
ENSMUSG00000022450	Ndufa6	0.45844609	0.01149114	3
ENSMUSG00000022452	Smdt1	1.00301768	7.84E-10	3
ENSMUSG00000022477	Aco2	0.6481377	1.32E-28	4
ENSMUSG00000022610	Mapk12	-0.7484403	3.23E-30	1
ENSMUSG00000022668	Gtpbp8	-0.5359319	2.54E-12	1
ENSMUSG00000022679	Mpv17l	1.44266007	1.17E-41	3
ENSMUSG00000022704	Qtrtd1	-1.7950811	2.83E-245	1
ENSMUSG00000022751	Nit2	0.73239294	2.53E-14	4
ENSMUSG00000022752	Tomm70a	-1.097256	8.10E-40	1
ENSMUSG00000022792	Yars2	-0.6190243	2.06E-21	1
ENSMUSG00000022797	Tfrc	-1.379734	3.87E-49	2
ENSMUSG00000022812	Gsk3b	-0.1800777	0.03568323	1
ENSMUSG00000022820	Ndufb4	0.78740272	9.49E-19	4
ENSMUSG00000022890	Atp5j	0.25982066	0.00095427	3
ENSMUSG00000022906	Parp9	-1.24878	6.04E-24	2
ENSMUSG00000022956	Atp5o	0.34574865	2.32E-07	3
ENSMUSG00000022982	Sod1	0.48179958	1.00E-09	3
ENSMUSG00000023020	Cox14	0.79703497	0.00693568	3
ENSMUSG00000023030	Slc11a2	-0.3742294	1.22E-17	1
ENSMUSG00000023034	Nr4a1	-4.2292103	0	1

ENSMUSG00000023087	Ccrn4l	-1.3882112	3.53E-113	2
ENSMUSG00000023089	Ndufa5	1.28493787	9.28E-35	4
ENSMUSG00000023094	Msrb2	0.80957297	0.0179781	4
ENSMUSG00000023805	Synj2	1.09231229	1.62E-67	4
ENSMUSG00000023827	Agpat4	0.57312634	1.31E-09	3
ENSMUSG00000023832	Acat2	1.09053387	1.17E-121	3
ENSMUSG00000023861	Mpc1	0.88988771	3.63E-24	4
ENSMUSG00000023921	Mut	1.64262661	1.07E-141	4
ENSMUSG00000023938	Aars2	0.70369757	0.00283306	3
ENSMUSG00000023939	Mrpl14	0.79162187	0.01026992	3
ENSMUSG00000024012	Mtch1	0.74705905	1.15E-16	4
ENSMUSG00000024030	Abcg1	1.26168767	7.33E-12	3
ENSMUSG00000024038	Ndufv3	1.46266682	9.27E-40	4
ENSMUSG00000024045	Akap8	-0.2097735	4.45E-07	2
ENSMUSG00000024082	Ndufaf7	1.12860985	3.83E-57	4
ENSMUSG00000024087	Cyp1b1	4.29962053	0	4
ENSMUSG00000024099	Ndufv2	0.90074217	5.55E-19	4
ENSMUSG00000024132	Eci1	0.24440619	0.00414366	3
ENSMUSG00000024158	Hagh	0.54608646	7.23E-18	3
ENSMUSG00000024177	Nme4	0.89051281	5.00E-14	4
ENSMUSG00000024191	Bnip1	0.91889966	1.58E-12	4
ENSMUSG00000024208	Uqcc2	0.82992846	8.88E-07	3
ENSMUSG00000024234	Mtpap	-0.6328166	9.59E-16	2
ENSMUSG00000024248	Cox7a2l	0.49606229	1.92E-19	3
ENSMUSG00000024259	Slc25a46	-0.531535	1.88E-14	2
ENSMUSG00000024349	Tmem173	-3.1109332	4.37E-127	3

ENSMUSG00000024359	Hspa9	-1.0700813	2.62E-40	1
ENSMUSG00000024378	Stard4	0.58527678	3.90E-27	3
ENSMUSG00000024414	Mrpl27	0.26762091	0.00485986	3
ENSMUSG00000024431	Nr3c1	-0.24885	0.02753035	2
ENSMUSG00000024436	Mrps18b	-0.6247605	1.61E-21	1
ENSMUSG00000024462	Gabbr1	0.27063125	6.18E-05	3
ENSMUSG00000024507	Hsd17b4	0.39681084	5.28E-11	4
ENSMUSG00000024521	Pmaip1	0.29947679	0.00010397	4
ENSMUSG00000024527	Afg3l2	-0.3580722	1.75E-24	1
ENSMUSG00000024530	Slmo1	-1.7813988	5.61E-20	1
ENSMUSG00000024533	Spire1	-1.4740292	5.71E-99	1
ENSMUSG00000024556	Me2	-0.2650763	0.00632416	2
ENSMUSG00000024580	Grpel2	-1.0293352	1.35E-49	1
ENSMUSG00000024587	Nars	-1.6311718	2.43E-114	1
ENSMUSG00000024588	Fech	0.59634961	4.92E-19	4
ENSMUSG00000024592	C330018D20Rik	-1.1079479	2.04E-16	2
ENSMUSG00000024608	Rps14	-0.3265115	0.04181656	2
ENSMUSG00000024617	Camk2a	5.67698641	0.00018656	4
ENSMUSG00000024645	Timm21	-0.2976573	0.03449897	1
ENSMUSG00000024646	Cyb5a	0.92715058	5.15E-24	3
ENSMUSG00000024659	Anxa1	2.24669962	2.88E-163	4
ENSMUSG00000024661	Fth1	1.558491	1.37E-87	3
ENSMUSG00000024683	Mrpl16	0.16547121	0.02281213	4
ENSMUSG00000024712	Rfk	0.80738317	2.82E-40	4
ENSMUSG00000024782	Ak3	1.39617215	4.18E-63	4
ENSMUSG00000024827	Gldc	2.19045363	5.65E-142	4

ENSMUSG00000024887	Asah2	0.76670294	1.32E-10	4
ENSMUSG00000024892	Pcx	1.90319244	1.18E-22	3
ENSMUSG00000024900	Cpt1a	1.15808955	1.80E-89	4
ENSMUSG00000024902	Mrpl11	0.59533569	2.22E-06	4
ENSMUSG00000024942	Capn1	1.49034675	1.24E-50	4
ENSMUSG00000024944	Arl2	0.76141713	0.00046794	4
ENSMUSG00000024953	Prdx5	-0.9691564	3.72E-06	1
ENSMUSG00000024959	Bad	0.62125519	0.02684046	3
ENSMUSG00000024963	Dnajc4	1.11138509	1.43E-08	3
ENSMUSG00000024978	Gpam	0.74556924	3.26E-13	4
ENSMUSG00000024981	Acsl5	0.76908466	2.94E-20	4
ENSMUSG00000024999	Noc3l	-1.3219893	1.65E-61	1
ENSMUSG00000025007	Aldh18a1	-1.0255773	9.46E-57	1
ENSMUSG00000025036	Sfxn2	0.25054937	0.0040373	4
ENSMUSG00000025037	Maoa	0.34412497	0.00014646	4
ENSMUSG00000025040	Fundc1	0.82817815	2.36E-14	4
ENSMUSG00000025059	Gk	0.36846757	5.05E-07	3
ENSMUSG00000025086	Trub1	-0.5109574	1.10E-15	1
ENSMUSG00000025140	Pycr1	-1.0961737	7.57E-18	1
ENSMUSG00000025150	Cbr2	6.7344776	2.54E-280	4
ENSMUSG00000025176	Hoga1	1.66266561	7.66E-29	4
ENSMUSG00000025178	Pi4k2a	-0.2039408	6.64E-06	1
ENSMUSG00000025204	Ndufb8	0.91606108	1.28E-16	4
ENSMUSG00000025209	Peo1	-0.889677	2.78E-151	1
ENSMUSG00000025212	Sfxn3	0.32406405	3.67E-10	4
ENSMUSG00000025220	Mgea5	-0.9040583	4.42E-32	2

ENSMUSG00000025224	Gbf1	0.41814253	5.87E-10	4
ENSMUSG00000025269	Apex2	-0.8646415	7.39E-40	2
ENSMUSG00000025277	Abhd6	1.22424877	5.39E-41	4
ENSMUSG00000025287	Acot9	-0.2376717	0.00634556	2
ENSMUSG00000025289	Prdx4	0.78712002	1.81E-15	4
ENSMUSG00000025393	Atp5b	0.25209633	6.97E-07	4
ENSMUSG00000025403	Shmt2	-1.3436235	4.75E-09	1
ENSMUSG00000025428	Atp5a1	0.2964131	2.25E-11	4
ENSMUSG00000025436	Xrcc6bp1	0.68023378	0.00010437	3
ENSMUSG00000025453	Nnt	0.3328759	2.84E-27	4
ENSMUSG00000025465	Echs1	0.19217499	0.017864	3
ENSMUSG00000025525	Apool	0.89789482	4.41E-17	4
ENSMUSG00000025545	Clybl	1.57224732	6.01E-13	4
ENSMUSG00000025582	Nptx1	-3.8306804	0.00022101	2
ENSMUSG00000025651	Uqcrc1	0.65751311	1.28E-05	4
ENSMUSG00000025665	Rps6ka6	2.3076014	1.29E-06	4
ENSMUSG00000025733	Rhot2	0.4677121	8.01E-05	3
ENSMUSG00000025745	Hadha	0.57509431	1.69E-41	4
ENSMUSG00000025781	Atp5c1	0.55526313	3.18E-17	4
ENSMUSG00000025792	Slc25a10	1.08715456	0.00027556	3
ENSMUSG00000025810	Nrp1	-0.4755315	3.02E-10	2
ENSMUSG00000025815	Dhtkd1	1.23004702	3.50E-08	4
ENSMUSG00000025825	Iscu	0.73295114	1.17E-50	3
ENSMUSG00000025889	Snca	1.06992205	0.04802703	4
ENSMUSG00000025911	Adhfe1	-0.9776407	0.00205074	3
ENSMUSG00000025933	Tmem14a	1.43922864	1.56E-06	4

ENSMUSG00000025937	Lactb2	0.30809292	4.40E-12	3
ENSMUSG00000025950	Idh1	2.6233119	0	4
ENSMUSG00000025958	Creb1	-0.5475513	2.41E-07	2
ENSMUSG00000025962	Fastkd2	-0.6316267	1.45E-26	2
ENSMUSG00000025971	9430016H08Rik	-0.4806964	2.85E-06	2
ENSMUSG00000025980	Hspd1	-0.5412217	1.88E-10	1
ENSMUSG00000025981	Coq10b	-0.9683976	1.88E-76	2
ENSMUSG00000025991	Cps1	-3.353266	0.00564918	1
ENSMUSG00000026028	Trak2	-0.6565737	1.21E-17	2
ENSMUSG00000026032	Ndufb3	0.65365487	4.30E-14	3
ENSMUSG00000026036	Nif3l1	-0.706422	4.25E-16	1
ENSMUSG00000026096	Osgepl1	0.63506973	1.07E-06	4
ENSMUSG00000026103	Gls	-0.516767	1.23E-07	2
ENSMUSG00000026142	Rhbdd1	-2.0451863	4.26E-206	1
ENSMUSG00000026154	Sdhaf4	1.15568711	4.88E-19	4
ENSMUSG00000026170	Cyp27a1	1.04483174	0.03705664	4
ENSMUSG00000026172	Bcs1l	0.8015804	2.51E-22	4
ENSMUSG00000026179	Pnkd	1.21319897	6.62E-21	3
ENSMUSG00000026189	Pecr	-1.2711096	3.48E-30	2
ENSMUSG00000026192	Atic	-0.9984297	0	1
ENSMUSG00000026198	Abcb6	0.60345482	0.03049598	4
ENSMUSG00000026248	Mrpl44	-0.2747455	0.00071471	1
ENSMUSG00000026255	Efhd1	2.66691295	0.01146085	3
ENSMUSG00000026260	Ndufa10	0.38681747	4.00E-11	4
ENSMUSG00000026273	Mterf4	-0.2623228	0.00064263	1
ENSMUSG00000026278	Bok	3.79633477	3.50E-123	4

ENSMUSG00000026281	Dtymk	0.19300588	0.00038665	3
ENSMUSG00000026385	Dbi	0.70672932	2.15E-13	3
ENSMUSG00000026425	Srgap2	0.25834434	0.00170715	4
ENSMUSG00000026433	Rab29	1.07626119	3.98E-20	4
ENSMUSG00000026456	Cyb5r1	-0.935173	6.25E-192	1
ENSMUSG00000026473	Glul	-0.1113591	0.03853553	3
ENSMUSG00000026489	Adck3	2.36032649	2.19E-13	4
ENSMUSG00000026492	Tfb2m	-0.8542084	1.06E-42	1
ENSMUSG00000026496	Parp1	0.77306527	1.03E-17	4
ENSMUSG00000026499	Acbd3	-0.4458398	2.13E-08	2
ENSMUSG00000026509	Capn2	1.01096507	1.01E-59	4
ENSMUSG00000026520	Pycr2	-0.5309142	0.01884834	1
ENSMUSG00000026526	Fh1	0.67919161	7.30E-25	4
ENSMUSG00000026554	Dcaf8	0.10932178	0.02655154	3
ENSMUSG00000026568	Mpc2	1.07376886	1.09E-22	4
ENSMUSG00000026618	Iars2	0.14405749	0.0263952	4
ENSMUSG00000026632	Tatdn3	1.37350103	1.66E-16	4
ENSMUSG00000026664	Phyh	1.58511722	1.63E-118	3
ENSMUSG00000026687	Aldh9a1	0.61644012	1.67E-63	3
ENSMUSG00000026701	Prdx6	-0.5968278	4.11E-19	2
ENSMUSG00000026709	Dars2	0.94172162	1.00E-30	4
ENSMUSG00000026784	Pdss1	-0.3311898	0.00063163	2
ENSMUSG00000026797	Stxbp1	0.1766312	0.00255742	3
ENSMUSG00000026819	Slc25a25	-0.6541252	4.47E-16	1
ENSMUSG00000026820	Ptges2	0.37090509	0.01035994	3
ENSMUSG00000026825	Dnm1	0.51638616	7.25E-05	4

ENSMUSG00000026827	Gpd2	0.19595923	0.0055621	4
ENSMUSG00000026853	Crat	1.13773402	6.75E-95	4
ENSMUSG00000026864	Hspa5	-1.3014479	2.63E-79	2
ENSMUSG00000026880	Stom	-1.4586362	0	1
ENSMUSG00000026895	Ndufa8	0.72708073	2.11E-09	4
ENSMUSG00000026932	Nacc2	2.62217397	3.85E-144	4
ENSMUSG00000027010	Slc25a12	1.09633242	2.44E-84	3
ENSMUSG00000027076	Timm10	-1.7220869	1.68E-69	1
ENSMUSG00000027086	Fastkd1	-0.5566289	7.27E-07	2
ENSMUSG00000027104	Atf2	-0.74907	1.58E-22	1
ENSMUSG00000027187	Cat	1.31926744	1.29E-60	4
ENSMUSG00000027199	Gatm	-4.3398695	0.00552593	2
ENSMUSG00000027223	Mapk8ip1	0.56450116	9.09E-08	3
ENSMUSG00000027227	Sord	0.97569731	6.54E-57	4
ENSMUSG00000027244	Atg13	-0.667149	1.13E-28	1
ENSMUSG00000027282	Mtch2	-0.7639522	3.98E-34	2
ENSMUSG00000027305	Ndufaf1	-0.2992722	0.0001627	2
ENSMUSG00000027323	Rad51	-0.9665335	1.47E-95	2
ENSMUSG00000027332	Ivd	0.63981685	2.42E-29	4
ENSMUSG00000027357	Crls1	-0.4476888	0.00137273	1
ENSMUSG00000027367	Stard7	0.2072992	0.01320174	3
ENSMUSG00000027371	Fahd2a	-6.6111445	7.39E-07	2
ENSMUSG00000027381	Bcl2l11	-0.1285517	0.04095491	1
ENSMUSG00000027384	Ndufaf5	0.51310673	0.00036365	4
ENSMUSG00000027406	Idh3b	0.10867119	0.03612487	3
ENSMUSG00000027424	Mgme1	-0.8032826	1.43E-26	1

ENSMUSG00000027495	Fam210b	1.03936126	2.39E-32	3
ENSMUSG00000027599	Armc1	0.26175528	0.0003609	4
ENSMUSG00000027601	Mtfr1	-0.8175657	5.67E-32	1
ENSMUSG00000027618	Nfs1	0.4483784	4.37E-09	3
ENSMUSG00000027637	1110008F13Rik	0.22959955	0.01890609	3
ENSMUSG00000027668	Mfn1	0.31139961	3.01E-08	4
ENSMUSG00000027673	Ndufb5	0.87400577	4.99E-19	4
ENSMUSG00000027679	Dnajc19	0.84077677	5.61E-14	3
ENSMUSG00000027709	Mccc1	0.56442863	9.90E-15	4
ENSMUSG00000027710	Acad9	0.2911593	3.22E-05	4
ENSMUSG00000027722	Spata5	-0.7097539	1.19E-15	1
ENSMUSG00000027765	P2ry1	-4.9663949	1.46E-05	2
ENSMUSG00000027770	Dhx36	-0.2965812	0.02100043	2
ENSMUSG00000027774	Gfm1	0.32156767	4.38E-15	3
ENSMUSG00000027804	Ppid	-0.763488	3.47E-21	1
ENSMUSG00000027809	Etfdh	0.59570235	5.73E-20	4
ENSMUSG00000027880	Slc25a54	3.31864478	0.01565222	4
ENSMUSG00000027937	Jtb	-0.4209708	6.41E-05	1
ENSMUSG00000027942	4933434E20Rik	-0.6655107	1.57E-52	1
ENSMUSG00000027944	Hax1	-0.7697696	4.19E-16	1
ENSMUSG00000027983	Cyp2u1	0.80649669	1.30E-05	3
ENSMUSG00000027984	Hadh	1.39378398	3.76E-57	4
ENSMUSG00000027994	Ccdc109b	0.2763389	4.76E-09	3
ENSMUSG00000028013	Ppa2	-0.5849049	3.89E-06	2
ENSMUSG00000028070	Apoa1bp	0.92590603	4.77E-48	4
ENSMUSG00000028085	Gatb	0.79537319	3.07E-18	3

ENSMUSG00000028093	Acp6	-0.2100512	0.01349059	1
ENSMUSG00000028102	Pex11b	0.8296919	1.84E-14	4
ENSMUSG00000028107	Tars2	0.38503905	5.92E-15	3
ENSMUSG00000028127	Abcd3	0.25755658	0.0003803	4
ENSMUSG00000028138	Adh5	-0.340066	4.22E-06	1
ENSMUSG00000028145	Them4	-0.3314126	0.01694141	1
ENSMUSG00000028149	Rap1gds1	-0.2432091	0.00799404	2
ENSMUSG00000028163	Nfkb1	-0.530397	1.70E-28	1
ENSMUSG00000028165	Cisd2	-0.2813039	1.47E-06	1
ENSMUSG00000028186	Uox	1.55475906	1.49E-08	3
ENSMUSG00000028194	Ddah1	0.26895683	0.03506102	4
ENSMUSG00000028218	Fam92a	1.18198731	2.47E-26	4
ENSMUSG00000028223	Decr1	1.27183986	7.67E-37	4
ENSMUSG00000028228	Cpne3	0.33320388	0.00799766	3
ENSMUSG00000028229	Rmdn1	0.7731947	2.87E-06	4
ENSMUSG00000028261	Ndufaf4	-0.70437	1.13E-15	1
ENSMUSG00000028282	Casp8ap2	-0.465151	1.30E-06	2
ENSMUSG00000028292	Rars2	0.48474503	1.26E-06	4
ENSMUSG00000028330	Ncbp1	-0.4475703	6.59E-15	2
ENSMUSG00000028383	Hsdl2	0.87374369	1.79E-51	4
ENSMUSG00000028398	Tmem261	0.59037489	3.87E-15	3
ENSMUSG00000028405	Aco1	0.73271261	1.96E-48	4
ENSMUSG00000028412	Slc44a1	1.10482733	2.10E-39	3
ENSMUSG00000028416	Bag1	0.27297285	0.00472722	4
ENSMUSG00000028430	Nol6	-0.7461168	3.11E-08	1
ENSMUSG00000028443	Nudt2	1.19569901	5.88E-41	4

ENSMUSG00000028453	Fancg	0.51166504	1.34E-08	4
ENSMUSG00000028455	Stoml2	-0.189253	4.70E-05	2
ENSMUSG00000028470	Hint2	1.33219733	1.86E-14	3
ENSMUSG00000028527	Ak4	-2.382706	1.89E-25	2
ENSMUSG00000028603	Scp2	1.33611316	2.08E-84	4
ENSMUSG00000028607	Cpt2	0.78249867	5.25E-33	4
ENSMUSG00000028622	Mrpl37	0.25426193	1.56E-05	4
ENSMUSG00000028648	Ndufs5	0.46933306	1.96E-06	3
ENSMUSG00000028653	Trit1	-0.3946629	4.29E-06	2
ENSMUSG00000028672	Hmgcl	0.35957232	6.48E-05	3
ENSMUSG00000028687	Mutyh	0.76776669	1.86E-05	3
ENSMUSG00000028710	Atpaf1	0.4415772	2.71E-08	4
ENSMUSG00000028737	Aldh4a1	1.20966333	1.96E-25	4
ENSMUSG00000028743	Akr7a5	1.03659893	0.00293936	3
ENSMUSG00000028756	Pink1	1.50919461	8.50E-36	4
ENSMUSG00000028789	Azin2	1.17932333	2.06E-06	4
ENSMUSG00000028792	Ak2	-0.2365297	0.00602831	1
ENSMUSG00000028861	Mrps15	0.68786215	1.88E-20	4
ENSMUSG00000028889	Yrdc	-1.2825237	3.74E-59	1
ENSMUSG00000028910	Mecr	0.27706009	8.95E-06	3
ENSMUSG00000028914	Casp9	0.7509875	1.60E-16	4
ENSMUSG00000028953	Abcf2	-0.8740208	0	1
ENSMUSG00000028964	Park7	1.33731281	1.44E-97	4
ENSMUSG00000028970	Abcb1b	0.30269226	7.80E-06	3
ENSMUSG00000028973	Abcb8	0.58244409	0.01115996	3
ENSMUSG00000028978	Nos3	3.07847548	0.00019969	3

ENSMUSG00000028980	H6pd	0.50378357	1.64E-07	3
ENSMUSG00000028982	Slc25a33	-0.9160017	2.13E-42	1
ENSMUSG00000028998	Tomm7	0.46073166	5.81E-05	3
ENSMUSG00000029017	Pmpcb	0.24149447	0.00021935	4
ENSMUSG00000029020	Mfn2	0.60322288	1.30E-36	4
ENSMUSG00000029036	Atad3a	-0.5532717	2.07E-11	1
ENSMUSG00000029098	Acox3	1.31174457	1.25E-48	4
ENSMUSG00000029167	Ppargc1a	1.47362234	1.32E-80	3
ENSMUSG00000029198	Grpel1	-0.1298453	0.00980107	1
ENSMUSG00000029199	Lias	0.42225747	1.84E-07	4
ENSMUSG00000029208	Guf1	1.03215387	3.57E-46	4
ENSMUSG00000029310	Nudt9	-0.4731978	1.83E-10	1
ENSMUSG00000029366	Dck	0.34508386	1.63E-05	4
ENSMUSG00000029376	Mthfd2l	0.32806571	0.0084873	3
ENSMUSG00000029432	Gbas	0.940802	3.58E-64	4
ENSMUSG00000029433	Diablo	0.37022238	1.59E-10	4
ENSMUSG00000029455	Aldh2	1.31584999	2.13E-09	4
ENSMUSG00000029456	Acad10	1.5284443	1.80E-16	4
ENSMUSG00000029486	Mrpl1	-0.427353	1.94E-08	1
ENSMUSG00000029499	Pxmp2	1.63194195	1.37E-10	4
ENSMUSG00000029500	Pgam5	-0.4776371	1.62E-14	1
ENSMUSG00000029507	Pus1	-0.8316885	1.40E-22	1
ENSMUSG00000029524	Sirt4	0.68230027	1.82E-15	3
ENSMUSG00000029535	Triap1	0.64214668	1.91E-26	4
ENSMUSG00000029536	Gatc	0.29133206	6.92E-05	4
ENSMUSG00000029545	Acads	1.48623624	1.19E-07	4

ENSMUSG00000029557	Ftsj2	0.81244905	3.88E-07	3
ENSMUSG00000029575	Mmab	2.10142467	5.95E-183	4
ENSMUSG00000029591	Ung	-0.9335967	1.09E-38	1
ENSMUSG00000029592	Usp30	0.15650497	0.02188428	4
ENSMUSG00000029596	Sdsl	5.5840927	3.03E-19	3
ENSMUSG00000029624	Ptcd1	-0.3819728	0.00135361	1
ENSMUSG00000029632	Ndufa4	0.77706354	8.20E-22	4
ENSMUSG00000029762	Akr1b8	0.72545287	6.09E-25	3
ENSMUSG00000029769	Ccdc136	1.4449731	4.46E-112	3
ENSMUSG00000029776	Hibadh	0.55938432	1.27E-18	4
ENSMUSG00000029777	Gars	-1.0316146	9.09E-29	1
ENSMUSG00000029802	Abcg2	1.82502494	1.80E-52	4
ENSMUSG00000029815	Malsu1	0.45499346	0.00557275	3
ENSMUSG00000029863	Casp2	-0.6170153	2.94E-21	2
ENSMUSG00000029864	Gstk1	-4.8137488	0.01781269	2
ENSMUSG00000029911	Ssbp1	0.31140374	0.00239855	3
ENSMUSG00000029918	Mrps33	0.78683239	4.20E-14	3
ENSMUSG00000029993	Nfu1	1.03188098	4.08E-40	4
ENSMUSG00000030007	Cct7	-0.4614129	2.26E-14	2
ENSMUSG00000030086	Chchd6	1.17627322	5.65E-23	4
ENSMUSG00000030088	Aldh1l1	4.73321147	0.00015701	4
ENSMUSG00000030161	Gabarapl1	-1.1853669	2.99E-285	1
ENSMUSG00000030246	Ldhb	-1.8284367	7.20E-141	2
ENSMUSG00000030265	Kras	-0.2760906	0.00597364	2
ENSMUSG00000030268	Bcat1	-1.1313565	8.69E-92	2
ENSMUSG00000030309	Caprin2	0.89846487	4.70E-10	4

ENSMUSG00000030337	Vamp1	1.55850192	2.59E-17	4
ENSMUSG00000030357	Fkbp4	-0.5163943	1.41E-13	2
ENSMUSG00000030376	Slc8a2	-2.3431004	0.0089541	2
ENSMUSG00000030409	Dmpk	3.04157252	7.39E-110	3
ENSMUSG00000030421	Uri1	-0.8309796	1.94E-43	1
ENSMUSG00000030451	Herc2	-0.6028514	1.82E-22	2
ENSMUSG00000030541	Idh2	0.95529992	2.86E-35	4
ENSMUSG00000030559	Rab38	-4.7088903	0.00147301	2
ENSMUSG00000030562	Nox4	5.27840495	6.68E-40	4
ENSMUSG00000030612	Mrpl46	-0.4300557	0.00178885	1
ENSMUSG00000030613	Ccdc90b	0.83092521	8.96E-07	4
ENSMUSG00000030615	Tmem126a	-0.8479744	2.06E-31	1
ENSMUSG00000030621	Me3	2.75966825	8.07E-16	4
ENSMUSG00000030652	Coq7	0.3677192	0.00095427	3
ENSMUSG00000030653	Pde2a	3.335562	2.87E-65	3
ENSMUSG00000030725	Lipt2	-0.4252821	0.0361974	1
ENSMUSG00000030744	Rps3	-0.5445486	4.73E-15	1
ENSMUSG00000030747	Dgat2	-1.0472218	2.29E-54	1
ENSMUSG00000030802	Bckdk	0.41449499	1.07E-08	3
ENSMUSG00000030869	Ndufab1	-0.3304297	1.70E-05	1
ENSMUSG00000030871	Ears2	-1.1561828	1.28E-40	1
ENSMUSG00000030879	Mrpl17	-0.5316296	1.76E-10	1
ENSMUSG00000030894	Tpp1	-0.1188222	0.01358564	3
ENSMUSG00000030934	Oat	-0.2008204	0.00223415	3
ENSMUSG00000030986	Dhx32	0.33765702	0.00010541	4
ENSMUSG00000031059	Ndufb11	1.05378285	1.51E-20	3

ENSMUSG00000031158	Timm17b	0.52839205	0.00182609	3
ENSMUSG00000031198	Fundc2	0.60012959	2.97E-07	4
ENSMUSG00000031200	Mtcp1	1.02605988	0.00013375	4
ENSMUSG00000031278	Acsl4	-0.6122021	8.40E-15	1
ENSMUSG00000031299	Pdha1	0.33243734	1.29E-10	4
ENSMUSG00000031333	Abcb7	0.30015078	3.21E-05	4
ENSMUSG00000031352	Hccs	-0.7638478	1.19E-41	1
ENSMUSG00000031360	Ctps2	0.99766336	6.80E-35	3
ENSMUSG00000031373	Car5b	3.08220499	1.21E-293	4
ENSMUSG00000031378	Abcd1	0.52306065	0.00221805	4
ENSMUSG00000031467	Agpat5	-0.3625836	5.47E-10	1
ENSMUSG00000031485	Prosc	-0.3012751	1.85E-16	1
ENSMUSG00000031516	Dctn6	0.2343154	0.04659466	4
ENSMUSG00000031533	Mrps31	-0.4184116	3.27E-08	1
ENSMUSG00000031574	Star	-0.4529382	0.00386067	2
ENSMUSG00000031584	Gsr	0.41971887	1.39E-20	4
ENSMUSG00000031633	Slc25a4	1.01233242	1.11E-21	4
ENSMUSG00000031641	Cbr4	1.20818062	1.41E-17	4
ENSMUSG00000031672	Got2	0.37343376	8.21E-43	3
ENSMUSG00000031700	Gpt2	-0.7901271	4.67E-19	1
ENSMUSG00000031710	Ucp1	-5.7120613	0.00050464	1
ENSMUSG00000031730	Dhodh	0.75931432	5.11E-08	3
ENSMUSG00000031740	Mmp2	-2.5744537	0	1
ENSMUSG00000031776	Arl2bp	0.96129898	2.57E-88	4
ENSMUSG00000031781	Ciapin1	-0.1776241	3.87E-06	3
ENSMUSG00000031782	Coq9	0.83586602	6.67E-49	3

ENSMUSG00000031808	Slc27a1	1.12518705	5.45E-05	3
ENSMUSG00000031818	Cox4i1	0.63757664	1.47E-05	3
ENSMUSG00000031819	Emc8	0.26490687	4.60E-05	3
ENSMUSG00000031901	Dus2	0.79289145	5.20E-27	3
ENSMUSG00000031903	Pla2g15	0.85375304	5.58E-29	3
ENSMUSG00000031924	Cyb5b	0.5548069	2.03E-20	3
ENSMUSG00000031958	Ldhd	2.30582021	5.53E-06	3
ENSMUSG00000031960	Aars	-0.2548883	0.0001925	1
ENSMUSG00000031967	Afg3l1	0.20557799	0.00215699	4
ENSMUSG00000031969	Acad8	0.28862522	4.56E-05	4
ENSMUSG00000031974	Abcb10	1.53072312	5.00E-190	4
ENSMUSG00000031985	Gnpat	1.22534336	1.69E-92	4
ENSMUSG00000032040	Dcps	-0.4429055	2.32E-12	2
ENSMUSG00000032044	Rpusd4	-0.2914563	0.00052387	2
ENSMUSG00000032046	Abhd12	0.47624777	3.94E-27	3
ENSMUSG00000032047	Acat1	0.86255888	8.97E-19	4
ENSMUSG00000032051	Fdx1	-0.804915	8.72E-13	1
ENSMUSG00000032060	Cryab	4.1331507	0	4
ENSMUSG00000032067	Pts	0.81247356	8.50E-08	2
ENSMUSG00000032097	Ddx6	-0.2827372	0.00405068	2
ENSMUSG00000032118	Fez1	-4.4266115	5.55E-52	2
ENSMUSG00000032178	Ilf3	-0.1894069	0.00047487	2
ENSMUSG00000032194	Kank2	0.47860537	0.00018485	4
ENSMUSG00000032263	Bckdhb	1.40632603	1.00E-64	4
ENSMUSG00000032293	Ireb2	-0.4909126	4.36E-06	2
ENSMUSG00000032294	Pkm	0.48399366	4.82E-05	3

ENSMUSG00000032314	Etfa	0.47921318	2.22E-07	4
ENSMUSG00000032330	Cox7a2	0.47031677	7.73E-07	4
ENSMUSG00000032348	Gsta4	3.75263021	6.99E-257	4
ENSMUSG00000032370	Lactb	1.37952848	1.37E-41	4
ENSMUSG00000032418	Me1	1.66788933	1.32E-119	4
ENSMUSG00000032459	Mrps22	-0.4754121	1.05E-12	2
ENSMUSG00000032527	Pccb	1.29764598	7.66E-23	4
ENSMUSG00000032607	Amt	1.35362461	6.55E-17	4
ENSMUSG00000032667	Pon2	0.62172023	2.22E-25	4
ENSMUSG00000032705	Exd2	0.43959797	0.00050397	4
ENSMUSG00000032754	Slc8b1	0.77940407	3.44E-11	3
ENSMUSG00000032786	Alas1	0.46491903	0.00025019	3
ENSMUSG00000032826	Ank2	-3.4275804	3.75E-205	1
ENSMUSG00000032841	Prr5l	-2.3264365	1.51E-100	1
ENSMUSG00000032860	P2ry2	-2.363209	6.30E-73	1
ENSMUSG00000032883	Acsl3	0.46875811	2.90E-18	3
ENSMUSG00000032902	Slc16a1	-0.6064736	2.29E-34	1
ENSMUSG00000032959	Pebp1	0.22183446	0.00353954	3
ENSMUSG00000033126	Ybey	0.40632134	0.00099906	4
ENSMUSG00000033157	Abhd10	-0.5623193	1.30E-09	1
ENSMUSG00000033216	Eefsec	0.2404063	0.03678986	3
ENSMUSG00000033429	Mcee	0.70801102	0.01426061	4
ENSMUSG00000033436	Armcx2	1.07086084	2.84E-69	4
ENSMUSG00000033491	Prss35	2.86969053	2.54E-12	3
ENSMUSG00000033538	Casp4	0.43775613	9.69E-15	3
ENSMUSG00000033624	Pdpr	0.81241498	9.62E-08	4

ENSMUSG00000033685	Ucp2	0.704339	1.51E-06	3
ENSMUSG00000033720	Sfxn5	0.96018835	2.94E-28	3
ENSMUSG00000033735	Spr	0.96720139	3.25E-13	3
ENSMUSG00000033751	Gadd45gip1	0.60463087	1.13E-06	3
ENSMUSG00000033845	Mrpl15	-0.5758282	4.06E-27	2
ENSMUSG00000033918	Parl	-0.2262404	0.00486591	1
ENSMUSG00000033933	Vhl	-0.5016409	3.55E-17	2
ENSMUSG00000034156	Bzrap1	4.25646125	3.74E-34	4
ENSMUSG00000034173	Zbed5	-0.457205	8.46E-06	1
ENSMUSG00000034203	Chchd4	-0.9972687	1.24E-48	1
ENSMUSG00000034248	Slc25a37	-1.3065109	1.97E-130	1
ENSMUSG00000034285	Nipsnap1	2.86532599	3.94E-57	4
ENSMUSG00000034424	Gcsh	0.25808547	0.00439829	4
ENSMUSG00000034442	Trmt5	-0.3320854	0.00378787	2
ENSMUSG00000034613	Ppm1h	-1.4916088	3.38E-68	3
ENSMUSG00000034708	Grn	0.47311644	1.09E-10	3
ENSMUSG00000034729	Mrps10	-0.259763	0.00028106	1
ENSMUSG00000034853	Acot11	1.44301864	0.00524895	4
ENSMUSG00000034875	Nudt19	-0.4551408	0.0001672	2
ENSMUSG00000035069	Oma1	1.43441636	2.10E-18	4
ENSMUSG00000035131	Brinp3	-3.1565609	7.31E-07	1
ENSMUSG00000035139	Secisbp2	-0.3898701	2.10E-10	1
ENSMUSG00000035142	Nubpl	1.44047295	1.95E-19	4
ENSMUSG00000035232	Pdk3	0.54716223	2.53E-11	4
ENSMUSG00000035517	Tdrd7	0.75866577	2.93E-25	4
ENSMUSG00000035559	Mpv17l2	0.91166798	2.61E-33	3

ENSMUSG00000035666	Gtf3c4	-0.4800084	8.01E-08	2
ENSMUSG00000035674	Ndufa3	0.56683752	6.57E-09	3
ENSMUSG00000035772	Mrps2	-0.4957171	2.85E-21	1
ENSMUSG00000035824	Tk2	0.29251115	0.00127645	3
ENSMUSG00000035885	Cox8a	0.64891676	0.00016049	3
ENSMUSG00000035891	Cerk	1.06742875	7.96E-67	3
ENSMUSG00000035900	Gramd4	-0.8307862	1.06E-11	1
ENSMUSG00000035936	Aldh5a1	1.8566203	1.69E-23	4
ENSMUSG00000035960	Apex1	-0.7686883	2.16E-08	1
ENSMUSG00000036138	Acaa1a	0.95872139	7.19E-07	3
ENSMUSG00000036199	Ndufa13	1.05567662	0.00040603	3
ENSMUSG00000036257	Pnpla8	-0.2907833	2.33E-07	1
ENSMUSG00000036273	Lrrk2	-1.3228003	1.86E-42	2
ENSMUSG00000036278	Macrod1	0.63911716	1.79E-12	3
ENSMUSG00000036285	Noa1	-0.3484158	3.82E-09	1
ENSMUSG00000036353	P2ry12	2.63821508	0.01950432	3
ENSMUSG00000036438	Calm2	0.44666436	1.67E-06	4
ENSMUSG00000036555	Iqce	0.74952057	1.42E-31	4
ENSMUSG00000036564	Ndrg4	2.35995596	5.20E-79	4
ENSMUSG00000036580	Spg20	0.58854181	7.53E-41	4
ENSMUSG00000036639	Nudt1	1.24206198	3.46E-29	4
ENSMUSG00000036698	Ago2	-0.3339651	0.00089367	2
ENSMUSG00000036835	Psenen	-0.4608824	8.01E-11	1
ENSMUSG00000036850	Mrpl41	0.78965467	4.48E-16	3
ENSMUSG00000036880	Acaa2	0.20258058	0.00779152	4
ENSMUSG00000036932	Aifm1	0.62813438	2.45E-36	4

ENSMUSG00000036943	Rab8b	-0.5257014	3.37E-10	2
ENSMUSG00000036955	Kif1bp	-0.1259549	0.04382599	2
ENSMUSG00000036975	Tmem177	0.46306918	7.59E-08	4
ENSMUSG00000036983	Tfb1m	0.64484572	5.07E-11	4
ENSMUSG00000037012	Hk1	0.80862062	5.16E-25	4
ENSMUSG00000037022	Mmaa	1.19187059	1.03E-11	4
ENSMUSG00000037152	Ndufc1	0.78450944	2.13E-16	3
ENSMUSG00000037196	Pacrg	4.5585425	0.00323174	4
ENSMUSG00000037242	Clic4	-0.8684217	4.24E-46	1
ENSMUSG00000037353	Letmd1	1.45820084	1.39E-80	4
ENSMUSG00000037499	Nenf	1.50720864	3.49E-31	3
ENSMUSG00000037514	Pank2	0.19025214	0.03403267	3
ENSMUSG00000037531	Mrpl47	-0.3052885	1.53E-05	1
ENSMUSG00000037601	Nme1	-0.5657166	9.31E-09	2
ENSMUSG00000037636	Slc25a43	2.86857516	4.63E-15	4
ENSMUSG00000037710	Cisd1	-0.2142706	0.03107385	1
ENSMUSG00000037747	Phyhipl	-6.7032427	5.24E-07	2
ENSMUSG00000037787	Apopt1	0.62203952	2.46E-13	4
ENSMUSG00000037805	Rpl10a	-0.5019713	1.85E-08	1
ENSMUSG00000037820	Tgm2	2.48569899	1.15E-142	4
ENSMUSG00000037826	Ppm1k	0.8679854	1.79E-16	3
ENSMUSG00000037860	Aim2	4.77810464	5.32E-17	3
ENSMUSG00000037916	Ndufv1	0.37511328	0.0217821	3
ENSMUSG00000037938	Chchd5	0.4829639	0.00137483	3
ENSMUSG00000037960	1110007C09Rik	1.48662168	1.61E-08	4
ENSMUSG00000037972	Snn	0.40831775	3.84E-19	4

ENSMUSG00000038028	Tigar	-0.3225951	0.0033766	2
ENSMUSG00000038046	Rnmtl1	-0.8414181	3.93E-24	1
ENSMUSG00000038068	Rnf144b	-1.5911248	0.00026095	2
ENSMUSG00000038121	Fam210a	-0.4357278	3.42E-13	2
ENSMUSG00000038195	Rilp	1.84330217	1.92E-06	3
ENSMUSG00000038225	Primpol	0.39550681	0.00024513	3
ENSMUSG00000038286	Bphl	2.86484138	4.32E-34	4
ENSMUSG00000038342	Mlxip	-0.2376118	0.02991907	2
ENSMUSG00000038393	Txnip	1.21129852	2.30E-32	4
ENSMUSG00000038422	Hdhd3	0.94208649	5.35E-12	3
ENSMUSG00000038518	Jarid2	-0.7056703	5.41E-15	1
ENSMUSG00000038567	Cyp24a1	4.40569671	9.09E-39	3
ENSMUSG00000038582	Pptc7	-0.2258649	0.03921359	1
ENSMUSG00000038612	Mcl1	-0.7197058	2.92E-15	1
ENSMUSG00000038633	Degs1	-1.2659496	8.97E-154	1
ENSMUSG00000038690	Atp5j2	0.30457865	0.00038765	3
ENSMUSG00000038733	Wdr26	0.25382029	0.01758899	4
ENSMUSG00000038780	Smurf1	-1.3778502	3.17E-91	1
ENSMUSG00000038845	Phb	-0.6478195	9.77E-19	2
ENSMUSG00000038872	Zfhx3	0.80314914	3.40E-10	4
ENSMUSG00000038880	Mrps34	0.56892022	1.02E-08	3
ENSMUSG00000038888	Ctu1	-1.2181797	1.43E-29	1
ENSMUSG00000038936	Sccpdh	1.21000819	2.45E-54	4
ENSMUSG00000038967	Pdk2	1.435031	7.62E-19	4
ENSMUSG00000039018	Mtg1	0.79284126	2.31E-08	4
ENSMUSG00000039048	Foxred1	-0.2024439	0.04033297	1

ENSMUSG00000039063	Echdc3	3.69703941	6.75E-75	4
ENSMUSG00000039065	Fam173b	0.42425346	1.89E-06	3
ENSMUSG00000039069	Mtg2	0.76168046	6.08E-17	4
ENSMUSG00000039163	Cmc1	0.71492162	4.47E-18	3
ENSMUSG00000039176	Polg	-0.4596259	3.01E-06	1
ENSMUSG00000039275	Foxk2	-0.2076854	1.42E-05	1
ENSMUSG00000039457	Ppl	-0.8804632	0.00072691	2
ENSMUSG00000039477	Tnrc18	1.063436	0.00988188	4
ENSMUSG00000039478	Micu3	-0.4283255	3.54E-05	2
ENSMUSG00000039530	Tusc3	0.87957212	9.92E-22	4
ENSMUSG00000039646	Vasn	-0.7721112	0.00418211	1
ENSMUSG00000039648	Ccbl1	0.90243712	0.0001248	3
ENSMUSG00000039670	Oxld1	0.74593064	4.35E-05	3
ENSMUSG00000039680	Mrps6	0.57659755	4.38E-11	3
ENSMUSG00000039682	Lap3	-0.1442387	8.25E-07	2
ENSMUSG00000039768	Dnajc11	-0.3528495	3.23E-07	1
ENSMUSG00000039826	Trub2	-0.2466815	0.00495893	2
ENSMUSG00000039958	Mettl20	1.23275133	1.05E-06	3
ENSMUSG00000040018	Cox15	0.40317333	1.59E-16	4
ENSMUSG00000040048	Ndufb10	0.64143654	2.54E-11	3
ENSMUSG00000040136	Abcc8	-3.4545992	2.45E-05	2
ENSMUSG00000040177	2310057M21Rik	-1.3736	5.69E-67	1
ENSMUSG00000040213	Ccbl2	-0.8055448	1.50E-06	2
ENSMUSG00000040265	Dnm3	2.56528565	0.00095531	4
ENSMUSG00000040269	Mrps28	0.2822656	0.00051698	3
ENSMUSG00000040322	Slc25a24	0.53914025	2.26E-19	4

ENSMUSG00000040354	Mars	-0.5044405	8.38E-27	1
ENSMUSG00000040370	Lyrm5	0.74732174	1.47E-15	4
ENSMUSG00000040410	Fbxl4	0.30760535	0.01071009	3
ENSMUSG00000040435	Ppp1r15a	-2.3471009	0	1
ENSMUSG00000040459	Arglu1	0.29592995	6.85E-05	4
ENSMUSG00000040464	Gtpbp10	-1.4164989	1.59E-48	1
ENSMUSG00000040483	Xaf1	-3.9171127	5.67E-74	3
ENSMUSG00000040506	Ambra1	-0.5603716	5.47E-16	1
ENSMUSG00000040532	Abhd11	-0.3808721	0.00015512	1
ENSMUSG00000040618	Pck2	-1.0440949	1.51E-16	1
ENSMUSG00000040675	Mthfd1l	-1.5315066	1.06E-176	1
ENSMUSG00000040740	Slc25a34	-2.6255209	0.00190256	3
ENSMUSG00000040820	Hlcs	0.56483803	0.00016749	4
ENSMUSG00000040822	1700123O20Rik	-0.8278293	1.01E-45	2
ENSMUSG00000040888	Gfer	-0.4303721	2.15E-07	1
ENSMUSG00000041028	Ghitm	-0.9939513	4.14E-60	1
ENSMUSG00000041064	Pif1	2.21047947	2.87E-40	4
ENSMUSG00000041135	Ripk2	-0.3046633	0.00242763	2
ENSMUSG00000041328	Pcf11	-1.09356	2.08E-69	1
ENSMUSG00000041426	Hibch	0.66349845	1.33E-09	4
ENSMUSG00000041431	Ccnb1	0.21876391	0.03377568	2
ENSMUSG00000041440	Gk5	-0.3562384	0.00995182	2
ENSMUSG00000041632	Mrps27	-0.3220639	0.012111	1
ENSMUSG00000041697	Cox6a1	0.76206075	0.00524493	3
ENSMUSG00000041736	Tspo	1.3819694	0.00258238	3
ENSMUSG00000041797	Abca9	-2.3306877	0.00092223	2

ENSMUSG00000041886	Macc1	7.91495959	1.08E-09	4
ENSMUSG00000041912	Tdrkh	2.92619729	5.71E-80	4
ENSMUSG00000041921	Metap1d	0.83876781	9.43E-50	4
ENSMUSG00000042010	Acacb	1.69273087	1.55E-34	3
ENSMUSG00000042082	Arsb	0.73407894	2.15E-25	4
ENSMUSG00000042148	Cox10	-0.3667434	0.00655391	1
ENSMUSG00000042198	Chchd7	0.71529344	1.42E-11	3
ENSMUSG00000042228	Lyn	-0.4019676	0.01700975	3
ENSMUSG00000042298	Ttc19	-0.197687	0.00224982	2
ENSMUSG00000042349	Ikbke	1.32766765	7.73E-50	3
ENSMUSG00000042410	Agps	-0.4021353	0.00025194	2
ENSMUSG00000042462	Dctpp1	-0.8731704	4.74E-09	1
ENSMUSG00000042505	Sdhaf3	0.89243084	4.55E-05	4
ENSMUSG00000042622	Maff	-1.8275506	6.23E-220	1
ENSMUSG00000042626	Shc1	-0.176948	9.21E-05	1
ENSMUSG00000042647	Acad12	1.86666682	1.81E-17	4
ENSMUSG00000042670	Immp1l	0.40369345	0.0099624	4
ENSMUSG00000042709	Atpaf2	0.24217903	0.03094444	4
ENSMUSG00000042770	Hebp1	0.34925198	0.00199662	3
ENSMUSG00000043140	Tmem186	-0.28447	0.00026615	1
ENSMUSG00000043162	Pyurf	1.33604266	1.44E-27	3
ENSMUSG00000043284	Tmem11	-1.294892	1.88E-74	1
ENSMUSG00000043411	Usp48	0.30728567	0.00172325	4
ENSMUSG00000043510	Hscb	1.49111619	1.03E-51	3
ENSMUSG00000043613	Mmp3	-4.8756371	0	1
ENSMUSG00000043702	Pde12	-0.4936079	9.91E-10	2

ENSMUSG00000043733	Ptpn11	-0.3802104	1.51E-20	2
ENSMUSG00000044005	Gls2	-0.3800964	0.0126036	2
ENSMUSG00000044018	Mrpl50	-0.8257289	5.33E-16	1
ENSMUSG00000044221	Grsf1	-0.4155672	1.06E-06	2
ENSMUSG00000044288	Cnr1	-6.8369169	5.75E-07	1
ENSMUSG00000044763	Trmt10c	-1.3145489	2.25E-52	1
ENSMUSG00000044783	Hjurp	-0.1389084	0.01498139	2
ENSMUSG00000044792	Isca1	-0.4825032	5.76E-09	1
ENSMUSG00000045160	Bola3	0.4990768	8.20E-06	3
ENSMUSG00000045316	Fahd1	0.32724704	0.00137337	3
ENSMUSG00000045409	Trim39	-0.3800299	1.21E-06	2
ENSMUSG00000045438	Cox19	0.63293059	2.68E-07	4
ENSMUSG00000045636	Mtus1	1.7065927	2.21E-74	3
ENSMUSG00000045854	Lyrm2	1.20058183	4.96E-14	4
ENSMUSG00000045973	Slc25a51	-0.1433302	0.00149548	1
ENSMUSG00000046329	Slc25a23	1.52321067	2.61E-62	3
ENSMUSG00000046442	Ppm1e	1.21672876	3.22E-19	4
ENSMUSG00000046573	Lyrm4	0.74416957	0.00038051	3
ENSMUSG00000046598	Bdh1	-9.599216	8.89E-15	2
ENSMUSG00000046603	Tcaim	0.3276334	0.02344123	4
ENSMUSG00000046607	Hrk	-7.4575251	4.19E-08	1
ENSMUSG00000046671	Mtfr1l	0.56298123	1.04E-59	3
ENSMUSG00000046808	Atp10d	-0.6579927	1.15E-08	1
ENSMUSG00000046909	Tefm	-0.4151504	0.00064989	1
ENSMUSG00000046947	Adck2	1.57232237	3.35E-34	4
ENSMUSG00000046994	Mars2	-1.0213788	1.19E-59	2

ENSMUSG00000047084	Ngrn	-0.6228424	9.92E-13	1
ENSMUSG00000047123	Ticam1	-0.2178337	0.04849758	2
ENSMUSG00000047205	Dusp18	1.2422971	1.85E-151	3
ENSMUSG00000047363	Cstad	3.9204289	0.01246246	4
ENSMUSG00000047635	2810006K23Rik	0.47503986	1.09E-10	4
ENSMUSG00000047649	Cd3eap	-1.6999831	9.04E-68	1
ENSMUSG00000047804	Akap10	-0.7041795	2.37E-16	1
ENSMUSG00000048007	Timm8a1	-1.3173709	1.49E-37	1
ENSMUSG00000048351	Coa7	-0.9926924	9.31E-102	1
ENSMUSG00000048371	Pdp2	-0.3687919	0.00017743	1
ENSMUSG00000048429	1810026J23Rik	-0.5768003	1.78E-15	2
ENSMUSG00000048482	Bdnf	-0.7958502	4.60E-12	2
ENSMUSG00000048731	Ggnbp1	1.55634693	0.00026346	4
ENSMUSG00000048755	Mcat	0.18473085	0.0361181	3
ENSMUSG00000048756	Foxo3	-1.331125	1.41E-113	1
ENSMUSG00000048826	Dact2	1.45757516	3.14E-25	4
ENSMUSG00000049038	Mterf2	0.61305022	4.02E-05	4
ENSMUSG00000049047	Armcx3	-0.3115669	1.01E-05	1
ENSMUSG00000049090	Zadh2	-1.3023007	2.41E-141	1
ENSMUSG00000049106	Dcaf5	0.36966285	4.87E-09	4
ENSMUSG00000049119	Fam110b	2.88374266	3.49E-32	4
ENSMUSG00000049225	Pdp1	0.73808918	3.33E-13	3
ENSMUSG00000049287	Iba57	-0.9665601	1.35E-12	1
ENSMUSG00000049422	Chchd10	-2.9718264	2.06E-144	1
ENSMUSG00000049760	2410015M20Rik	0.22289464	0.03377568	3
ENSMUSG00000049792	Bag5	-0.4239949	4.21E-15	2

ENSMUSG00000049858	Suox	2.49051936	3.90E-123	4
ENSMUSG00000050043	Tmx2	-0.365283	1.50E-05	1
ENSMUSG00000050144	Slc25a44	-0.5590632	6.23E-20	1
ENSMUSG00000050174	Nudt6	0.84594445	0.00014809	3
ENSMUSG00000050244	Heatr1	-1.6062003	1.24E-105	2
ENSMUSG00000050312	Nsun3	0.38587834	3.89E-05	3
ENSMUSG00000050323	Ndufaf6	0.58347872	0.00010557	4
ENSMUSG00000050335	Lgals3	-1.2399828	6.60E-15	1
ENSMUSG00000050394	Armcx6	0.7682912	0.00148535	2
ENSMUSG00000050608	Minos1	0.66747977	1.65E-12	3
ENSMUSG00000050705	2310061I04Rik	-0.1433432	0.04570665	1
ENSMUSG00000050732	Vamp8	0.85091738	2.52E-19	4
ENSMUSG00000050856	Atp5k	0.87131858	2.11E-20	3
ENSMUSG00000050953	Gja1	-2.4195915	0	1
ENSMUSG00000050965	Prkca	0.53907599	4.68E-07	2
ENSMUSG00000051043	Gprc5c	4.98579005	0.00025448	4
ENSMUSG00000051236	Msrb3	2.01134104	8.87E-169	4
ENSMUSG00000051319	1500011K16Rik	1.11336968	1.03E-05	3
ENSMUSG00000051343	Rab11fip5	2.06881652	3.28E-86	4
ENSMUSG00000051346	Spryd4	-0.2935438	0.00074643	1
ENSMUSG00000051451	Crebzf	-0.2241473	0.0057639	1
ENSMUSG00000051671	Coa6	0.43154325	3.13E-07	3
ENSMUSG00000052131	Akr1b7	5.21400303	1.06E-06	3
ENSMUSG00000052214	Opa3	-0.6403952	7.97E-24	1
ENSMUSG00000052459	Atp6v1a	0.30799177	3.69E-08	3
ENSMUSG00000052852	Reep1	-2.2458153	9.59E-31	2

ENSMUSG00000053253	Ndfip2	-1.0958222	5.49E-106	2
ENSMUSG00000053329	D10Jhu81e	0.86134233	0.00036796	3
ENSMUSG00000053436	Mapk14	0.47176839	5.39E-38	4
ENSMUSG00000053644	Aldh7a1	-1.9570894	1.92E-127	2
ENSMUSG00000053647	Gper1	2.91915007	1.77E-20	3
ENSMUSG00000053746	Ptrh1	-0.2547214	0.0020099	3
ENSMUSG00000053768	Chchd3	0.51152849	9.80E-20	3
ENSMUSG00000053898	Ech1	0.47795312	2.11E-17	3
ENSMUSG00000054021	Sirt5	0.85851634	1.98E-08	4
ENSMUSG00000054027	Nt5dc3	-1.5670316	1.30E-109	1
ENSMUSG00000054099	Slc25a40	0.57469016	1.51E-16	3
ENSMUSG00000054312	Mrps21	0.99194925	7.27E-27	3
ENSMUSG00000054428	Atpif1	0.63984921	3.49E-05	4
ENSMUSG00000054676	1600014C10Rik	0.61856716	1.29E-05	3
ENSMUSG00000054894	Atp5s	1.37861872	2.90E-11	4
ENSMUSG00000054942	Fam73a	1.81381453	1.99E-35	4
ENSMUSG00000055137	Sugct	3.65045804	3.58E-05	4
ENSMUSG00000055184	Fam72a	-0.6253955	0.0010441	1
ENSMUSG00000055737	Ghr	-1.0361437	3.46E-31	1
ENSMUSG00000055782	Abcd2	0.87773787	0.00031421	3
ENSMUSG00000055926	Gm14137	0.49283851	0.01983657	4
ENSMUSG00000056201	Cfl1	0.59393475	1.08E-33	4
ENSMUSG00000056228	Cars2	1.04842294	8.23E-28	4
ENSMUSG00000056234	Ncoa4	-0.5576453	2.17E-12	2
ENSMUSG00000056394	Lig1	-0.2096404	0.00039098	1
ENSMUSG00000056427	Slit3	-7.866122	3.48E-239	1

ENSMUSG00000056671	Prelid2	1.29414783	3.28E-07	4
ENSMUSG00000056899	Immp2l	2.54753118	1.52E-10	4
ENSMUSG00000056999	Ide	0.28574624	0.00333511	4
ENSMUSG00000057134	Ado	-0.4485048	8.65E-18	1
ENSMUSG00000057193	Slc44a2	0.23457469	6.16E-09	3
ENSMUSG00000057229	Atp5sl	-0.7252949	1.47E-14	1
ENSMUSG00000057329	Bcl2	1.9010004	6.69E-47	4
ENSMUSG00000057363	Uxs1	-0.2170284	0.00259977	2
ENSMUSG00000057388	Mrpl18	0.42248571	1.24E-07	4
ENSMUSG00000057411	Fam173a	1.15727252	9.59E-07	3
ENSMUSG00000057497	Fam136a	-0.6262112	1.96E-15	1
ENSMUSG00000057666	Gapdh	0.39046082	0.00772398	4
ENSMUSG00000057789	Bak1	0.38668341	0.00600713	4
ENSMUSG00000058076	Sdhc	0.53883807	1.48E-15	4
ENSMUSG00000058267	Mrps14	0.58954244	5.65E-12	4
ENSMUSG00000058351	Smim4	0.93451039	5.53E-08	3
ENSMUSG00000058355	Abce1	-0.3433147	0.00045125	2
ENSMUSG00000058586	Serhl	2.01689417	3.58E-84	4
ENSMUSG00000058756	Thra	1.05212602	5.19E-21	4
ENSMUSG00000058979	Cecr5	0.29489989	0.00620136	4
ENSMUSG00000058997	Vwa8	0.59986299	1.52E-12	4
ENSMUSG00000059183	Mtfmt	0.58744805	6.26E-13	4
ENSMUSG00000059447	Hadhb	0.73863431	2.00E-12	4
ENSMUSG00000059552	Trp53	-1.0688817	1.52E-53	2
ENSMUSG00000059734	Ndufs8	0.76460081	3.09E-05	4
ENSMUSG00000059743	Fdps	0.97825397	8.53E-62	3

ENSMUSG00000060636	Rpl35a	-0.77008	7.80E-17	1
ENSMUSG00000060679	Mrps9	-0.418687	3.16E-07	3
ENSMUSG00000060681	Slc9a6	0.75579651	8.07E-20	4
ENSMUSG00000060803	Gstp1	0.47696953	2.11E-11	3
ENSMUSG00000060923	Acyp2	1.65524883	4.77E-14	4
ENSMUSG00000061461	Smim20	0.9527268	1.17E-24	3
ENSMUSG00000061474	Mrps36	0.46153864	1.10E-07	3
ENSMUSG00000061758	Akr1b10	1.96842889	3.34E-89	4
ENSMUSG00000061838	Suclg2	0.90839048	1.98E-66	4
ENSMUSG00000061904	Slc25a3	-0.3117694	1.72E-06	1
ENSMUSG00000062006	Rpl34	-0.4049748	6.40E-05	1
ENSMUSG00000062209	Erbb4	-4.1806141	0.04142468	2
ENSMUSG00000062480	Acat3	1.15753357	1.02E-05	3
ENSMUSG00000062488	Ifit3b	-3.2634252	0.00014195	3
ENSMUSG00000062691	Cebpzos	2.36659912	1.86E-61	4
ENSMUSG00000062729	Ppox	0.68567537	4.66E-09	3
ENSMUSG00000062908	Acadm	0.82928248	1.80E-39	4
ENSMUSG00000062981	Mrpl42	0.32139697	0.00292524	4
ENSMUSG00000063077	Kif1b	0.41378484	9.64E-05	4
ENSMUSG00000063179	Pstk	0.49493564	1.78E-06	3
ENSMUSG00000063229	Ldha	0.10605413	0.04267315	4
ENSMUSG00000063235	Ptpmt1	-0.722281	2.85E-13	2
ENSMUSG00000063450	Syne2	0.31305844	0.00136418	4
ENSMUSG00000063698	Sfxn4	1.86994924	8.23E-21	4
ENSMUSG00000063856	Gpx1	1.09218908	4.33E-07	4
ENSMUSG00000063882	Uqcrh	0.56716122	3.36E-07	3

ENSMUSG00000063884	Ptcd3	-0.267652	0.00049531	2
ENSMUSG00000064037	Gpn1	-0.4397399	3.12E-11	2
ENSMUSG00000064068	Mtx1	-0.5282782	0.00013513	1
ENSMUSG00000064090	Vrk2	0.41426881	0.00053348	4
ENSMUSG00000064215	Ifi27	-0.9449624	3.16E-25	1
ENSMUSG00000064337	mt-Rnr1	1.06774523	1.04E-17	4
ENSMUSG00000064339	mt-Rnr2	0.93852882	1.65E-12	4
ENSMUSG00000064341	mt-Nd1	1.22270672	1.52E-29	4
ENSMUSG00000064345	mt-Nd2	1.05730227	2.51E-24	4
ENSMUSG00000064348	mt-Tn	0.62830031	0.01159112	4
ENSMUSG00000064349	mt-Tc	0.68979018	0.00098552	4
ENSMUSG00000064351	mt-Co1	0.61819596	3.32E-10	4
ENSMUSG00000064354	mt-Co2	0.63038792	0.00091268	1
ENSMUSG00000064356	mt-Atp8	0.58032769	0.00017441	3
ENSMUSG00000064357	mt-Atp6	0.53328112	2.28E-08	3
ENSMUSG00000064360	mt-Nd3	1.95547396	0.011756	4
ENSMUSG00000064363	mt-Nd4	0.90037369	1.29E-12	4
ENSMUSG00000064367	mt-Nd5	1.30737342	6.98E-37	4
ENSMUSG00000064368	mt-Nd6	1.25475933	3.07E-33	4
ENSMUSG00000064370	mt-Cytb	0.64529536	3.34E-10	4
ENSMUSG00000065990	Aurkaip1	0.36515195	0.00099058	3
ENSMUSG00000066595	Mfsd7b	-0.8207796	9.18E-20	2
ENSMUSG00000066798	Zbtb6	-0.2931093	2.44E-05	2
ENSMUSG00000066800	Rnasel	1.82844633	2.11E-05	4
ENSMUSG00000067369	Trmt2b	1.45684101	1.23E-41	4
ENSMUSG00000068206	Pick1	0.8320294	8.41E-12	4

ENSMUSG00000068523	Gng5	-0.3414082	4.13E-11	1
ENSMUSG00000068739	Sars	-1.4240823	3.38E-151	1
ENSMUSG00000068823	Csde1	-0.3462742	2.16E-05	2
ENSMUSG00000068921	Dap3	-0.298632	2.58E-08	3
ENSMUSG00000068922	Msto1	0.2060877	0.03704186	4
ENSMUSG00000069844	Sco1	-0.8006601	1.86E-26	1
ENSMUSG00000069874	Irgm2	-2.3071014	1.82E-64	3
ENSMUSG00000070283	Ndufaf3	1.24014688	2.43E-17	3
ENSMUSG00000070284	Gmppb	0.44483176	0.00250934	4
ENSMUSG00000070394	Tmem256	0.74344678	4.73E-24	3
ENSMUSG00000070699	Sars2	0.53893668	0.02566186	3
ENSMUSG00000070730	Rmdn3	-0.4706687	3.74E-11	2
ENSMUSG00000071253	Slc25a16	0.31124227	2.92E-05	3
ENSMUSG00000071528	Usmg5	0.32600352	0.02157159	3
ENSMUSG00000071711	Mpst	1.02648105	0.00228523	3
ENSMUSG00000071724	Smpd5	1.07119002	0.00280755	4
ENSMUSG00000072582	Ptrh2	-0.649539	2.07E-22	1
ENSMUSG00000072946	Ptgr2	0.64703684	2.30E-20	4
ENSMUSG00000073481	2-Mar	1.04784512	7.93E-24	4
ENSMUSG00000073609	D2hgdh	1.14162095	2.10E-13	4
ENSMUSG00000073676	Hspe1	-0.4392443	7.44E-08	1
ENSMUSG00000074064	Mlycd	1.09775745	2.62E-35	3
ENSMUSG00000074211	Sdhaf1	-0.5538056	0.01901355	2
ENSMUSG00000074896	Ifit3	-0.6547744	0.00408033	3
ENSMUSG00000075467	Dnlz	0.37947453	2.02E-11	3
ENSMUSG00000075604	Cyp11b1	5.50490781	0.02552465	3

ENSMUSG00000075704	Txnrd2	0.99732832	1.03E-24	4
ENSMUSG00000076431	Sox4	-1.4173008	3.59E-183	1
ENSMUSG00000076435	Acsf2	1.22386218	1.37E-84	4
ENSMUSG00000076441	Ass1	-0.7220629	1.27E-11	2
ENSMUSG00000078139	AK157302	-0.4680803	0.04549983	1
ENSMUSG00000078566	Bnip3	0.34747866	0.00047304	3
ENSMUSG00000078572	1810043H04Rik	0.84878928	5.75E-05	3
ENSMUSG00000078656	Vps25	0.86478109	0.04879528	3
ENSMUSG00000078713	Tomm5	-0.4574741	1.61E-08	1
ENSMUSG00000078716	Tmem8b	2.74193994	1.74E-19	4
ENSMUSG00000079017	Ifi27l2a	-1.4383064	0.02064513	1
ENSMUSG00000079036	Alkbh1	-0.7157205	1.66E-17	2
ENSMUSG00000079037	Prnp	0.3677325	2.65E-12	3
ENSMUSG00000079043	Fastkd5	-0.5084955	0.00022311	2
ENSMUSG00000079055	Slc8a3	2.52732247	7.98E-05	3
ENSMUSG00000079508	Apoo	0.74122312	9.24E-05	4
ENSMUSG00000079555	Haus3	-0.6140762	1.46E-09	2
ENSMUSG00000086784	Isoc2a	-0.7855549	1.76E-05	3
ENSMUSG00000087687	Pet100	1.38880883	9.47E-20	4
ENSMUSG00000089682	Bcl2l2	-0.5983938	7.88E-15	2
ENSMUSG00000090110	Cmc4	0.74476608	0.00343437	4
ENSMUSG00000090247	Bloc1s1	1.22641523	1.43E-15	3
ENSMUSG00000090877	Hspa1b	0.77830364	3.34E-31	3
ENSMUSG00000090935	Synj2bp	-0.5196732	8.50E-06	2

B. 68 OXPHOS -related genes that are DE (p-adj < 0.05) between WT4 and KO4 cells.

gene.ids	gene.name	WT4 vs KO4

		log2FC	p-adj
ENSMUSG00000000171	Sdhd	-0.1983493	0.00195243
ENSMUSG00000004446	Bid	0.27668073	2.37E-07
ENSMUSG00000005373	Mlxipl	-5.3026336	5.80E-09
ENSMUSG00000006457	Actn3	3.71337188	7.94E-13
ENSMUSG00000013593	Ndufs2	0.58420549	6.02E-30
ENSMUSG00000014554	Dguok	0.6293764	3.18E-17
ENSMUSG00000015790	Surf1	0.50405635	1.42E-13
ENSMUSG00000020022	Ndufa12	-0.3629585	5.71E-05
ENSMUSG00000020664	Dld	-0.3576807	5.05E-07
ENSMUSG00000021520	Uqcrb	0.3221319	0.00234061
ENSMUSG00000021577	Sdha	0.20066657	0.00065839
ENSMUSG00000021868	Ppif	-0.8059533	1.84E-30
ENSMUSG00000022013	Dnajc15	0.25785153	0.04725355
ENSMUSG00000022346	Myc	-2.0979364	3.56E-114
ENSMUSG00000022354	Ndufb9	0.6491557	1.36E-14
ENSMUSG00000022890	Atp5j	0.25982066	0.00095427
ENSMUSG00000022956	Atp5o	0.34574865	2.32E-07
ENSMUSG00000024038	Ndufv3	1.46266682	9.27E-40
ENSMUSG00000024099	Ndufv2	0.90074217	5.55E-19
ENSMUSG00000024151	Msh2	0.59415333	8.12E-23
ENSMUSG00000024208	Uqcc2	0.82992846	8.88E-07
ENSMUSG00000024248	Cox7a2l	0.49606229	1.92E-19
ENSMUSG00000025204	Ndufb8	0.91606108	1.28E-16
ENSMUSG00000025393	Atp5b	0.25209633	6.97E-07
ENSMUSG00000025403	Shmt2	-1.3436235	4.75E-09
ENSMUSG00000025428	Atp5a1	0.2964131	2.25E-11
ENSMUSG00000025651	Uqcrc1	0.65751311	1.28E-05
ENSMUSG00000025781	Atp5c1	0.55526313	3.18E-17
ENSMUSG00000025825	Iscu	0.73295114	1.17E-50

ENSMUSG00000025889	Snca	1.06992205	0.04802703
ENSMUSG00000026260	Ndufa10	0.38681747	4.00E-11
ENSMUSG00000026895	Ndufa8	0.72708073	2.11E-09
ENSMUSG00000027282	Mtch2	-0.7639522	3.98E-34
ENSMUSG00000027305	Ndufaf1	-0.2992722	0.0001627
ENSMUSG00000028455	Stoml2	-0.189253	4.70E-05
ENSMUSG00000028527	Ak4	-2.382706	1.89E-25
ENSMUSG00000028756	Pink1	1.50919461	8.50E-36
ENSMUSG00000028964	Park7	1.33731281	1.44E-97
ENSMUSG00000028982	Slc25a33	-0.9160017	2.13E-42
ENSMUSG00000029432	Gbas	0.940802	3.58E-64
ENSMUSG00000030652	Coq7	0.3677192	0.00095427
ENSMUSG00000031378	Abcd1	0.52306065	0.00221805
ENSMUSG00000031393	Mecp2	-0.6349368	2.87E-16
ENSMUSG00000031782	Coq9	0.83586602	6.67E-49
ENSMUSG00000031818	Cox4i1	0.63757664	1.47E-05
ENSMUSG00000032330	Cox7a2	0.47031677	7.73E-07
ENSMUSG00000033751	Gadd45gip1	0.60463087	1.13E-06
ENSMUSG00000037916	Ndufv1	0.37511328	0.0217821
ENSMUSG00000038690	Atp5j2	0.30457865	0.00038765
ENSMUSG00000039065	Fam173b	0.42425346	1.89E-06
ENSMUSG00000041431	Ccnb1	0.21876391	0.03377568
ENSMUSG00000041697	Cox6a1	0.76206075	0.00524493
ENSMUSG00000043702	Pde12	-0.4936079	9.91E-10
ENSMUSG00000046329	Slc25a23	1.52321067	2.61E-62
ENSMUSG00000046909	Tefm	-0.4151504	0.00064989
ENSMUSG00000048482	Bdnf	-0.7958502	4.60E-12
ENSMUSG00000049422	Chchd10	-2.9718264	2.06E-144
ENSMUSG00000058076	Sdhc	0.53883807	1.48E-15
ENSMUSG00000059734	Ndufs8	0.76460081	3.09E-05

ENSMUSG00000062070	Pgk1	0.55334599	4.66E-09
ENSMUSG00000063882	Uqcrh	0.56716122	3.36E-07
ENSMUSG00000064345	mt-Nd2	1.05730227	2.51E-24
ENSMUSG00000064351	mt-Co1	0.61819596	3.32E-10
ENSMUSG00000064354	mt-Co2	0.63038792	0.00091268
ENSMUSG00000064357	mt-Atp6	0.53328112	2.28E-08
ENSMUSG00000064363	mt-Nd4	0.90037369	1.29E-12
ENSMUSG00000064367	mt-Nd5	1.30737342	6.98E-37
ENSMUSG00000064370	mt-Cytb	0.64529536	3.34E-10

C. Paired t-test summary results

**1. Mitochondria-related genes (n=1136)**						
**Pair-wise comparisson (A vs. B)**	**P-value**	**P-value summary**	**Mean of differences (B-A)**	**SD of differences (B-A)**	**SEM of differences (B-A)**	**95% confidence interval of differences**
WTM vs. WT4	0.0605	ns	0.06795	1.219	0.03616	-0.003007 to 0.1389
WT4 vs. WT14	< 0.0001	****	0.1539	0.9763	0.02897	0.09703 to 0.2107
WTM vs WT14	< 0.0001	****	0.2218	1.059	0.03141	0.1602 to 0.2834
KOM vs. KO4	< 0.0001	****	0.1701	0.8783	0.02606	0.1190 to 0.2212
KO4 vs. KO14	< 0.0001	****	0.1636	0.7082	0.02101	0.1223 to 0.2048
KOM vs KO14	< 0.0001	****	0.3336	1.004	0.02979	0.2752 to 0.3921
WTM vs. KOM	0.6028	ns	-0.009347	0.6053	0.01796	-0.04458 to 0.02589
WT4 vs. KO4	0.0006	***	0.09279	0.9115	0.02704	0.03972 to 0.1458
WT14 vs. KO14	<0.0001	****	0.1025	0.6299	0.01869	0.06581 to 0.1392
**2. OXPHOS-related genes (n=68)**						
**Pair-wise comparisson (A vs. B)**	**P-value**	**P-value summary**	**Mean of differences (B-A)**	**SD of differences (B-A)**	**SEM of differences (B-A)**	**95% confidence interval of differences**
WTM vs. WT4	0.6463	ns	-0.07643	1.367	0.1658	-0.4073 to 0.2545
WT4 vs. WT14	< 0.0001	****	0.4011	0.7645	0.09271	0.2161 to 0.5862
WTM vs WT14	0.0339	*	0.3247	1.236	0.1499	0.02547 to 0.6239
KOM vs. KO4	0.0498	*	0.2622	1.082	0.1312	0.0002907 to 0.5242

KO4 vs. KO14	0.0041	**	0.2235	0.6195	0.07513	0.07355 to 0.3735
KOM vs KO14	0.0017	**	0.4857	1.227	0.1488	0.1877 to 0.7827
WTM vs. KOM	0.604	ns	-0.03416	0.5405	0.06555	-0.1650 to 0.09667
WT4 vs. KO4	0.0026	**	0.3045	0.8028	0.09735	0.1102 to 0.4988
WT14 vs. KO14	0.0146	*	0.1269	0.4172	0.05059	0.02588 to 0.2278

*Note that these results were performed on VST-normalized data, and therefore not on z-score normalized data.

D. Gene Ontology (GO) - KEGG Pathway analysis

**Cluster 1 (n=268)**				
**KEGG Pathway**	**Gene count**	**PValue**	**Bonferroni**	**Benjamini**
One carbon pool by folate	7	3.56E-07	7.32E-05	7.32E-05
Aminoacyl-tRNA biosynthesis	9	9.56E-06	0.0019673	9.84E-04
Biosynthesis of antibiotics	13	1.62E-04	0.03287415	0.01108037
Biosynthesis of amino acids	8	2.08E-04	0.04191317	0.01064713
Metabolic pathways	37	3.10E-04	0.06186667	0.01269142
Ribosome	10	5.29E-04	0.10323299	0.01799597
Carbon metabolism	8	0.00260806	0.41606281	0.07397282
PI3K-Akt signaling pathway	14	0.0041714	0.57730563	0.10204762
FoxO signaling pathway	8	0.00578969	0.69764177	0.12445152
Prolactin signaling pathway	6	0.00629814	0.72788206	0.122038
Selenocompound metabolism	3	0.02994278	0.99809342	0.43408602
TNF signaling pathway	6	0.03108646	0.99850474	0.41848674
Neurotrophin signaling pathway	6	0.04687873	0.99994936	0.53271852
Sphingolipid signaling pathway	6	0.04966426	0.99997229	0.52741842

**Cluster 2 (n=194)**				
**KEGG Pathway**	**Gene count**	**PValue**	**Bonferroni**	**Benjamini**
Metabolic pathways	32	2.23E-04	0.0436656	0.0436656
Biosynthesis of antibiotics	11	4.39E-04	0.08399395	0.04291795
Neurotrophin signaling pathway	8	0.00100562	0.18227218	0.06487516
Pyruvate metabolism	5	0.00156099	0.26834205	0.07513781
Peroxisome	6	0.00438069	0.58441305	0.16105714

Central carbon metabolism in cancer	5	0.00941928	0.84934824	0.27054971
Hepatitis B	7	0.01167774	0.90456338	0.28510048
Alanine, aspartate and glutamate metabolism	4	0.01203351	0.91119389	0.26115157
Arginine biosynthesis	3	0.02478675	0.9933943	0.42750892
Arginine and proline metabolism	4	0.02552213	0.99431937	0.40373453
MicroRNAs in cancer	9	0.02683938	0.99566584	0.39021995
Valine, leucine and isoleucine degradation	4	0.03438194	0.99908573	0.44184305
D-Glutamine and D-glutamate metabolism	2	0.0385066	0.99961162	0.45344294
Huntington's disease	7	0.04379956	0.99987124	0.47261507
Pancreatic cancer	4	0.05221541	0.99997803	0.51082765
Thyroid cancer	3	0.05413626	0.99998535	0.50127948
Insulin resistance	5	0.05467847	0.99998694	0.48393953

**Cluster 3 (n=279)**				
**KEGG Pathway**	**Gene count**	**PValue**	**Bonferroni**	**Benjamini**
Metabolic pathways	82	2.07E-26	3.41E-24	3.41E-24
Oxidative phosphorylation	27	6.09E-19	1.00E-16	5.02E-17
Alzheimer's disease	27	3.05E-16	5.50E-14	1.83E-14
Parkinson's disease	24	6.67E-15	1.10E-12	2.75E-13
Huntington's disease	26	4.91E-14	8.10E-12	1.62E-12
Non-alcoholic fatty liver disease (NAFLD)	19	1.13E-09	1.86E-07	3.10E-08
Peroxisome	13	5.74E-08	9.47E-06	1.35E-06
Fatty acid degradation	8	4.03E-05	0.00663452	8.32E-04
Fatty acid metabolism	8	5.26E-05	0.00864636	9.64E-04
Biosynthesis of antibiotics	15	6.89E-05	0.01131068	0.00113687
Pyruvate metabolism	7	9.55E-05	0.0156325	0.00143134
Carbon metabolism	10	4.15E-04	0.06615989	0.00568793
Citrate cycle (TCA cycle)	5	0.00326818	0.41732943	0.0406974
Biosynthesis of amino acids	7	0.00354293	0.44323999	0.04096727
Valine, leucine and isoleucine degradation	6	0.00419144	0.49994661	0.0451516
PPAR signaling pathway	7	0.0045703	0.53037824	0.04614082
Glycerolipid metabolism	6	0.00526954	0.58179032	0.04998806

Arginine biosynthesis	4	0.00560253	0.60426731	0.05019721
Aminoacyl-tRNA biosynthesis	6	0.00907984	0.77798625	0.07615537
ABC transporters	5	0.01202106	0.86405338	0.09495865
Tryptophan metabolism	5	0.01294457	0.88349241	0.09730575
Arginine and proline metabolism	5	0.01492464	0.91635113	0.1066515
Propanoate metabolism	4	0.01509693	0.91873077	0.10338606
Sulfur relay system	3	0.01534447	0.9220325	0.100855
Cardiac muscle contraction	6	0.01694194	0.94035657	0.10664823
Fatty acid biosynthesis	3	0.02949015	0.9928387	0.1730111

Cluster 4 (n=395)				
**KEGG Pathway**	**Gene count**	**PValue**	**Bonferroni**	**Benjamini**
Metabolic pathways	104	7.26E-30	1.50E-27	1.50E-27
Parkinson's disease	38	2.22E-26	4.60E-24	2.30E-24
Oxidative phosphorylation	32	1.12E-20	2.31E-18	7.70E-19
Alzheimer's disease	35	2.09E-20	4.34E-18	1.08E-18
Carbon metabolism	29	9.63E-20	1.99E-17	3.99E-18
Valine, leucine and isoleucine degradation	21	2.76E-18	5.72E-16	9.54E-17
Biosynthesis of antibiotics	32	5.50E-15	1.15E-12	1.64E-13
Huntington's disease	29	2.38E-13	4.93E-11	6.16E-12
Fatty acid degradation	16	1.04E-12	2.14E-10	2.38E-11
Glyoxylate and dicarboxylate metabolism	13	3.25E-12	6.73E-10	6.73E-11
Citrate cycle (TCA cycle)	13	1.32E-11	2.73E-09	2.48E-10
Non-alcoholic fatty liver disease (NAFLD)	23	1.26E-10	2.61E-08	2.17E-09
Peroxisome	17	3.88E-10	8.03E-08	6.18E-09
Propanoate metabolism	11	8.27E-10	1.71E-07	1.22E-08
Fatty acid metabolism	13	5.94E-09	1.23E-06	8.20E-08
Pyruvate metabolism	9	6.72E-06	0.00138959	8.69E-05
Tryptophan metabolism	9	2.86E-05	0.00590029	3.48E-04
Butanoate metabolism	6	6.41E-04	0.12429834	0.00734676
2-Oxocarboxylic acid metabolism	5	0.00138703	0.24972504	0.01500811
Aminoacyl-tRNA biosynthesis	8	0.00177243	0.30734215	0.01819342

PPAR signaling pathway	8	0.0052883	0.66632358	0.05092351
ABC transporters	6	0.00722989	0.77732088	0.06599529
Long-term potentiation	7	0.00801637	0.81101284	0.06987668
GnRH signaling pathway	8	0.00884568	0.84105598	0.07377072
beta-Alanine metabolism	5	0.01095028	0.89763499	0.08713604
Lysine degradation	6	0.01205965	0.91885544	0.09207821
Apoptosis	6	0.02138126	0.9885975	0.15270032
Fatty acid elongation	4	0.03091032	0.99849568	0.20715048
Arginine and proline metabolism	5	0.04086668	0.9998226	0.25757362
Neurotrophin signaling pathway	8	0.04412951	0.99991238	0.26759135
Amyotrophic lateral sclerosis (ALS)	5	0.04628503	0.99994509	0.27126544

Table S2. OBD Analyses

A. DE genes in each pair-wise comparison

Pairwise comparison (A vs. B)	Significantly DE genes*	Upregulated in B	Downregulated in B
KOM vs. WTM	2124	1208	916
KO4 vs. WT4	4617	2678	1939
KO14 vs. WT14	3401	1431	1970
WTM vs. WT4	7995	4647	3348
WT4 vs. WT14	5784	2035	3749
WTM vs. WT14	6501	2981	3520
KOM vs. KO4	5606	3211	2395
KO4 vs KO14	3301	1712	1589
KOM vs. KO14	6236	3400	2836

*Significantly DE genes includes genes with p-adj < 0.05 and log2FC > 1 or log2FC < -1 from differential expression analysis using DESeq2

B. GO summary (WT)

WTM vs. WT4			
**Term**	**Count**	**PValue**	**Fold Enrichment**
osteoblast differentiation	53	7.60E-06	1.75697909

positive regulation of canonical Wnt signaling pathway	34	6.51E-05	1.90096132
negative regulation of canonical Wnt signaling pathway	45	2.36E-04	1.65265236
positive regulation of osteoblast differentiation	32	6.09E-04	1.76282918
endochondral ossification	17	0.00173442	2.12274014
negative regulation of osteoblast differentiation	26	0.00282951	1.73921986
intramembranous ossification	6	0.00631014	3.74601202
positive regulation of BMP signaling pathway	18	0.0065126	1.87300601
positive regulation of Wnt signaling pathway	18	0.00909743	1.82238422
positive regulation of bone mineralization	18	0.00909743	1.82238422
bone trabecula formation	7	0.01398443	2.9135649
bone resorption	13	0.01813321	1.94792625
Wnt signaling pathway, planar cell polarity pathway	9	0.02552832	2.24760721
regulation of Wnt signaling pathway	10	0.02928646	2.08111779
regulation of bone mineralization	11	0.03201367	1.96219677

**WT4 vs. WT14**			
**Term**	**Count**	**PValue**	**Fold Enrichment**
positive regulation of osteoblast differentiation	36	2.19E-07	2.327455271
osteoblast differentiation	48	5.79E-06	1.867456737
negative regulation of canonical Wnt signaling pathway	42	5.74E-05	1.810242989
ossification	38	3.51E-04	1.740203825
positive regulation of bone mineralization	19	4.78E-04	2.257561719
positive regulation of canonical Wnt signaling pathway	28	9.58E-04	1.837261541
negative regulation of Wnt signaling pathway	24	0.00164511	1.884130457
positive regulation of BMP signaling pathway	16	0.00870008	1.953913067
endochondral ossification	14	0.00973819	2.05160872
negative regulation of ossification	11	0.01052959	2.302826115
positive regulation of Wnt signaling pathway	16	0.01161132	1.901104606
canonical Wnt signaling pathway	31	0.01174502	1.531297038
cellular response to BMP stimulus	15	0.0165345	1.884130457

negative regulation of osteoblast differentiation	21	0.01882571	1.64861415
BMP signaling pathway involved in heart development	5	0.02688102	3.663587001
bone trabecula formation	6	0.03335116	2.9308696
positive regulation of osteoclast differentiation	11	0.04005521	1.934373936
bone morphogenesis	13	0.04262751	1.785998663
regulation of canonical Wnt signaling pathway	10	0.04365866	1.998320182

C. GO summary (KO)

KOM vs. KO4			
**Term**	**Count**	**PValue**	**Fold Enrichment**
osteoblast differentiation	41	8.34E-06	2.00818049
endochondral ossification	17	1.37E-05	3.13635343
bone mineralization	18	3.64E-05	2.84643841
positive regulation of osteoblast differentiation	25	5.32E-04	2.03483138
Wnt signaling pathway, calcium modulating pathway	7	6.93E-04	4.84289868
negative regulation of ossification	11	0.00183541	2.89915023
positive regulation of bone mineralization	15	0.00359786	2.24381406
ossification	29	0.00515372	1.67195312
negative regulation of canonical Wnt signaling pathway	30	0.00661664	1.6278651
intramembranous ossification	5	0.0116847	4.61228446
bone trabecula formation	6	0.01266169	3.68982757
positive regulation of BMP signaling pathway	13	0.02040186	1.9986566
positive regulation of cell proliferation in bone marrow	5	0.02338456	3.95338668
positive regulation of ossification	9	0.0423823	2.16576836
positive regulation of canonical Wnt signaling pathway	19	0.04791899	1.56955352

KO4 vs. KO14			
**Term**	**Count**	**PValue**	**Fold Enrichment**
osteoblast differentiation	38	2.41E-08	2.64722358
positive regulation of osteoblast differentiation	23	1.99E-05	2.66259059
positive regulation of osteoclast differentiation	11	5.69E-04	3.46368306
positive regulation of Wnt signaling pathway	13	0.001476	2.76584029

regulation of osteoclast differentiation	8	0.00320929	3.70447387
bone morphogenesis	11	0.00487061	2.70600239
positive regulation of bone mineralization	12	0.00491355	2.55308334
positive regulation of bone resorption	8	0.00907348	3.14880279
bone mineralization	11	0.00971819	2.47405933
ossification	21	0.01666437	1.72200152
cellular response to BMP stimulus	10	0.02740224	2.24914485
endochondral ossification	9	0.029817	2.36160209
negative regulation of canonical Wnt signaling pathway	21	0.03067359	1.62070732
negative regulation of bone mineralization	6	0.03295687	3.14880279
positive regulation of canonical Wnt signaling pathway	15	0.03976366	1.76238962
positive regulation of cell proliferation in bone marrow	4	0.04819914	4.49828969
ossification involved in bone maturation	4	0.04819914	4.49828969

Table S3. OBD, Wnt and BMP clusters.
The 3 pair-wise DE significance comparisons refer to:

1. WTM vs. KOM
2. WT4 vs KO4
3. WT14 vs KO14

Genes that are involved in positive regulation of OBD are marked with '1', and genes that are involved with negative regulation of OBD are marked with '-1'.

A. OBD: clusters, significance and GO analysis

Cluster assignment			Pairwise DE significance			Gene Ontology	
Cluster	Gene name	Gene ID	WTM v KOM	WT4 v KO4	WT14 v KO14	Pos reg of OBD	Neg reg of OBD
1	Acvr1	ENSMUSG00000026836	****	.	.	1	0
1	Jag1	ENSMUSG00000027276	****	****	****	1	0
1	Lgr4	ENSMUSG00000050199	****	.	.	0	0
1	Fgf2	ENSMUSG00000037225	****	****	.	1	0
1	Tmem64	ENSMUSG00000043252	****	****	.	0	-1
1	Tmem119	ENSMUSG00000054675	****	****	.	1	0
1	Smoc1	ENSMUSG00000021136	****	****	***	0	0
1	Rspo2	ENSMUSG00000051920	****	****	.	0	0
1	Pdlim7	ENSMUSG00000021493	****	.	.	1	0
1	Ilk	ENSMUSG00000030890	**	.	.	1	0
1	Trp63	ENSMUSG00000022510	**	****	.	1	0

1	Ptger4	ENSMUSG00000039942	****	****	****	1	0
1	Msx2	ENSMUSG00000021469	****	****	****	1	0
1	Lox	ENSMUSG00000024529	****	****	****	0	0
1	Actn3	ENSMUSG00000006457	****	****	*	0	0
1	Id3	ENSMUSG00000007872	****	****	.	0	-1
2	Id4	ENSMUSG00000021379	.	.	*	0	0
2	Nog	ENSMUSG00000048616	***	.	.	0	-1
2	Ccrn4l	ENSMUSG00000023087	.	****	.	0	-1
2	Fbn2	ENSMUSG00000024598	.	****	***	1	0
2	Igf1	ENSMUSG00000020053	****	**	*	1	0
2	Wnt10b	ENSMUSG00000022996	.	****	****	1	0
2	Wnt4	ENSMUSG00000036856	.	****	****	1	0
2	Id2	ENSMUSG00000020644	****	****	****	0	-1
2	Dlk1	ENSMUSG00000040856	****	*	.	0	-1
2	Mef2c	ENSMUSG00000005583	.	****	****	1	0
2	Sox11	ENSMUSG00000063632	****	****	****	1	0
2	Hoxa2	ENSMUSG00000014704	.	*	***	0	-1
2	Smo	ENSMUSG00000001761	.	.	****	0	0
2	Hey1	ENSMUSG00000040289	**	*	.	1	0
2	Fgf23	ENSMUSG00000000182	.	.	*	0	-1
2	Sfrp2	ENSMUSG00000027996	.	****	****	1	0
2	Mir23a	ENSMUSG00000065611	.	*	.	0	-1
2	Hdac7	ENSMUSG00000022475	.	****	.	0	-1
2	Prkaca	ENSMUSG00000005469	.	****	.	0	0
2	Zhx3	ENSMUSG00000035877	.	****	.	1	0
3	Lrrc17	ENSMUSG00000039883	.	*	****	0	0
3	Pth1r	ENSMUSG00000032492	*	*	****	0	0
3	Chrd	ENSMUSG00000006958	**	***	***	0	-1
3	Bmp4	ENSMUSG00000021835	.	.	****	1	0
3	Wnt11	ENSMUSG00000015957	*	****	****	0	0
3	Zfp932	ENSMUSG00000066613	.	.	****	0	-1
3	Lrp3	ENSMUSG00000001802	*	**	****	1	0
3	Sfrp1	ENSMUSG00000031548	****	****	****	0	-1
3	Gli1	ENSMUSG00000025407	.	****	***	0	0
3	Dhh	ENSMUSG00000023000	.	.	***	0	0
3	Sox8	ENSMUSG00000024176	*	**	****	0	0
3	Il6st	ENSMUSG00000021756	****	.	.	1	0
3	Ddr2	ENSMUSG00000026674	****	.	.	1	0
3	Scube2	ENSMUSG00000007279	*	.	.	1	0
3	Bmp6	ENSMUSG00000039004	.	****	***	1	0
3	Wnt7b	ENSMUSG00000022382	****	****	**	1	0

3	Bmp2	ENSMUSG00000027358	****	****	.	1	0
3	Axin2	ENSMUSG00000000142	****	.	****	0	-1
3	Sema4d	ENSMUSG00000021451	****	.	.	0	-1
3	Cthrc1	ENSMUSG00000054196	****	****	****	1	0
4	Bglap3	ENSMUSG00000074489	.	*	.	0	0
4	Adar	ENSMUSG00000027951	.	****	.	0	0
4	Shox2	ENSMUSG00000027833	****	.	****	0	0
4	Ifi204	ENSMUSG00000073489	.	.	****	1	0
4	Il6	ENSMUSG00000025746	****	****	.	1	0
4	Jund	ENSMUSG00000071076	.	****	.	1	0
4	Cebpd	ENSMUSG00000071637	****	****	.	1	0
4	Vegfa	ENSMUSG00000023951	****	****	.	1	0
4	Tgfbr3	ENSMUSG00000029287	****	.	.	0	0
4	Spp1	ENSMUSG00000029304	****	.	.	0	0
4	Fam20c	ENSMUSG00000025854	****	.	****	1	0
4	Rassf2	ENSMUSG00000027339	****	****	****	0	0
4	Id1	ENSMUSG00000042745	.	****	.	0	-1
5	Smad4	ENSMUSG00000024515	.	****	.	0	0
5	Sh3pxd2b	ENSMUSG00000040711	.	****	.	0	0
5	2410016O06Rik	ENSMUSG00000046791	.	****	.	0	-1
5	Bmpr1b	ENSMUSG00000052430	.	****	****	1	0
5	Ahr	ENSMUSG00000019256	.	****	****	0	-1
5	Rest	ENSMUSG00000029249	.	****	.	0	0
5	Mir133a-2	ENSMUSG00000065460	.	**	.	0	-1
5	Tnn	ENSMUSG00000026725	.	****	****	0	-1
5	Ranbp3l	ENSMUSG00000048424	.	****	.	0	-1
5	Gja1	ENSMUSG00000050953	.	****	.	0	0
5	Areg	ENSMUSG00000029378	***	****	.	0	-1
5	Rorb	ENSMUSG00000036192	.	****	****	0	-1
5	Epha2	ENSMUSG00000006445	.	****	.	0	0
5	Sox2	ENSMUSG00000074637	.	****	.	0	-1
5	Twist2	ENSMUSG00000007805	.	****	****	0	-1
5	Cebpa	ENSMUSG00000034957	.	****	.	1	0
5	Igfbp5	ENSMUSG00000026185	.	****	****	0	-1
5	Nppc	ENSMUSG00000026241	.	****	****	1	0
5	Cited1	ENSMUSG00000051159	.	****	.	0	-1
5	Wwtr1	ENSMUSG00000027803	.	****	.	0	0
5	Acvr2a	ENSMUSG00000052155	.	.	****	1	0
5	Mef2d	ENSMUSG00000001419	.	.	****	0	0
5	Fgfr1	ENSMUSG00000031565	****	.	****	0	-1
5	Acvr2b	ENSMUSG00000061393	.	**	.	1	0

5	Gdpd2	ENSMUSG00000019359	.	****	.	1	0
5	Fzd1	ENSMUSG00000044674	.	****	.	0	0
5	Grem1	ENSMUSG00000074934	****	****	.	0	-1

B. Wnt pathway: clusters and significance

Cluster assignment			Pairwise DE significance		
cluster	gene.name	gene.id	WTM v KOM	WT4 v KO4	WT14 v KO14
1	Apoe	ENSMUSG00000002985	.	****	.
1	Ruvbl2	ENSMUSG00000003868	.	**	.
1	Tle1	ENSMUSG00000008305	.	****	.
1	Ndel1	ENSMUSG00000018736	.	****	.
1	Egfr	ENSMUSG00000020122	****	.	.
1	Nle1	ENSMUSG00000020692	.	****	.
1	Sfrp4	ENSMUSG00000021319	****	.	****
1	Ror2	ENSMUSG00000021464	****	****	****
1	Nkd2	ENSMUSG00000021567	****	****	****
1	Fgf10	ENSMUSG00000021732	.	****	.
1	Dab2	ENSMUSG00000022150	****	****	.
1	Myc	ENSMUSG00000022346	.	****	.
1	Csnk1e	ENSMUSG00000022433	.	****	.
1	Ddit3	ENSMUSG00000025408	****	****	.
1	Mark1	ENSMUSG00000026620	.	****	****
1	Wwtr1	ENSMUSG00000027803	.	****	.
1	Ppap2b	ENSMUSG00000028517	****	.	.
1	Cdk14	ENSMUSG00000028926	.	****	.
1	Fgfr2	ENSMUSG00000030849	****	****	.
1	Ilk	ENSMUSG00000030890	**	.	.
1	Zranb1	ENSMUSG00000030967	.	****	.
1	Pkd2	ENSMUSG00000034462	.	****	.
1	Prickle1	ENSMUSG00000036158	****	****	****
1	Lrrk2	ENSMUSG00000036273	.	****	****
1	Siah2	ENSMUSG00000036432	.	****	.
1	Lrp1	ENSMUSG00000040249	****	.	.
1	Mdfic	ENSMUSG00000041390	****	.	.
1	Notum	ENSMUSG00000042988	.	.	****
1	Fzd1	ENSMUSG00000044674	.	****	.
1	Fzd5	ENSMUSG00000045005	****	.	****

1	Frat2	ENSMUSG00000047604	.	****	****
1	Foxo3	ENSMUSG00000048756	.	****	.
1	Arntl	ENSMUSG00000055116	.	****	.
1	Hmga2	ENSMUSG00000056758	****	****	****
1	Csnk1g3	ENSMUSG00000073563	.	****	.
1	Sox2	ENSMUSG00000074637	.	****	.
1	Grem1	ENSMUSG00000074934	****	****	.
1	Sox4	ENSMUSG00000076431	****	****	.
2	Sox9	ENSMUSG00000000567	.	****	****
2	Ccne1	ENSMUSG00000002068	****	.	.
2	Tiam1	ENSMUSG00000002489	.	.	****
2	Klf4	ENSMUSG00000003032	****	.	****
2	Grk5	ENSMUSG00000003228	.	****	.
2	Ndrg2	ENSMUSG00000004558	****	****	****
2	Sulf2	ENSMUSG00000006800	****	.	.
2	Gprc5b	ENSMUSG00000008734	.	.	****
2	Nkx2-5	ENSMUSG00000015579	.	.	*
2	Gata3	ENSMUSG00000015619	.	.	****
2	Sulf1	ENSMUSG00000016918	****	****	****
2	Rspo3	ENSMUSG00000019880	.	****	.
2	Sdc1	ENSMUSG00000020592	****	.	.
2	Slc9a3r1	ENSMUSG00000020733	.	****	.
2	Scel	ENSMUSG00000022123	.	.	****
2	Sema5a	ENSMUSG00000022231	****	.	****
2	Cby1	ENSMUSG00000022428	.	****	.
2	Cela1	ENSMUSG00000023031	.	****	****
2	Jade1	ENSMUSG00000025764	****	.	****
2	Tnn	ENSMUSG00000026725	.	****	****
2	Tbl1xr1	ENSMUSG00000027630	.	.	****
2	Tlr2	ENSMUSG00000027995	.	****	.
2	Vgll4	ENSMUSG00000030315	.	****	.
2	Fam53b	ENSMUSG00000030956	.	****	.
2	Porcn	ENSMUSG00000031169	.	****	.
2	Sall1	ENSMUSG00000031665	****	****	****
2	Tbx18	ENSMUSG00000032419	.	****	.
2	Tle6	ENSMUSG00000034758	****	****	.
2	Tle2	ENSMUSG00000034771	.	****	.
2	Mitf	ENSMUSG00000035158	.	.	****
2	Fzd8	ENSMUSG00000036904	****	.	.
2	Daam2	ENSMUSG00000040260	****	****	****
2	Fbxw4	ENSMUSG00000040913	****	.	.

2	Dact2	ENSMUSG00000048826	****	****	.
2	Fzd4	ENSMUSG00000049791	**	.	.
2	Aes	ENSMUSG00000054452	.	****	.
2	Frat1	ENSMUSG00000067199	.	*	.
2	Rnf213	ENSMUSG00000070327	.	.	****
2	Foxl1	ENSMUSG00000097084	.	****	*
3	Col1a1	ENSMUSG00000001506	****	****	.
3	Itga3	ENSMUSG00000001507	.	****	****
3	Cav1	ENSMUSG00000007655	.	****	.
3	Fzd3	ENSMUSG00000007989	.	****	****
3	Rac3	ENSMUSG00000018012	****	****	.
3	Lgr5	ENSMUSG00000020140	****	.	*
3	Grb10	ENSMUSG00000020176	****	.	.
3	Hnf1b	ENSMUSG00000020679	.	.	**
3	Ccdc88c	ENSMUSG00000021182	**	.	***
3	Depdc1b	ENSMUSG00000021697	.	****	.
3	Wnt5a	ENSMUSG00000021994	****	.	****
3	Ctnnd2	ENSMUSG00000022240	.	.	**
3	Wnt10b	ENSMUSG00000022996	.	****	****
3	Ptk7	ENSMUSG00000023972	.	.	****
3	Cdh2	ENSMUSG00000024304	.	.	****
3	Tle4	ENSMUSG00000024642	****	.	.
3	Wnt10a	ENSMUSG00000026167	.	.	****
3	Tnik	ENSMUSG00000027692	****	**	*
3	Lef1	ENSMUSG00000027985	.	****	****
3	Sfrp2	ENSMUSG00000027996	.	****	****
3	Reck	ENSMUSG00000028476	.	****	.
3	Mllt3	ENSMUSG00000028496	.	****	****
3	Wnt16	ENSMUSG00000029671	***	.	.
3	Tgfb1i1	ENSMUSG00000030782	****	****	.
3	Adgra2	ENSMUSG00000031486	.	.	****
3	Dixdc1	ENSMUSG00000032064	****	****	.
3	Kank1	ENSMUSG00000032702	.	****	.
3	Pkd1	ENSMUSG00000032855	.	****	.
3	Aspm	ENSMUSG00000033952	.	****	.
3	Rnf43	ENSMUSG00000034177	****	****	**
3	Daam1	ENSMUSG00000034574	.	.	****
3	Ttc21b	ENSMUSG00000034848	.	****	.
3	Ror1	ENSMUSG00000035305	**	.	.
3	Wnt4	ENSMUSG00000036856	.	****	****
3	Fgf2	ENSMUSG00000037225	****	****	.

3	Cpe	ENSMUSG00000037852	.	.	****
3	Ankrd6	ENSMUSG00000040183	.	**	****
3	Fzd7	ENSMUSG00000041075	.	****	.
3	Mgat3	ENSMUSG00000042428	.	****	****
3	Lgr6	ENSMUSG00000042793	.	****	**
3	Tmem64	ENSMUSG00000043252	****	****	.
3	Lgr4	ENSMUSG00000050199	****	.	.
3	Fzd2	ENSMUSG00000050288	.	****	.
3	Lypd6	ENSMUSG00000050447	****	****	****
3	Rspo2	ENSMUSG00000051920	****	****	.
3	Epm2a	ENSMUSG00000055493	.	.	***
3	Thra	ENSMUSG00000058756	.	****	.
3	Bcl9l	ENSMUSG00000063382	**	***	.
3	Ccnd1	ENSMUSG00000070348	.	****	****
3	Peg12	ENSMUSG00000070526	.	.	****
3	Trabd2b	ENSMUSG00000070867	****	****	*
3	Apcdd1	ENSMUSG00000071847	****	.	.
3	Mcc	ENSMUSG00000071856	.	****	.
4	Wnt9a	ENSMUSG00000000126	.	.	****
4	Axin2	ENSMUSG00000000142	****	.	****
4	Wnt2	ENSMUSG00000010797	.	****	.
4	Bicc1	ENSMUSG00000014329	.	****	.
4	Wnt11	ENSMUSG00000015957	*	****	****
4	Celsr1	ENSMUSG00000016028	.	****	****
4	Apc2	ENSMUSG00000020135	.	.	****
4	Wif1	ENSMUSG00000020218	****	.	****
4	Atp6v1c2	ENSMUSG00000020566	.	.	***
4	Gsc	ENSMUSG00000021095	.	.	*
4	Barx1	ENSMUSG00000021381	.	***	****
4	Tert	ENSMUSG00000021611	****	****	**
4	Lats2	ENSMUSG00000021959	.	****	.
4	Amer2	ENSMUSG00000021986	.	.	**
4	Wnt7b	ENSMUSG00000022382	****	****	**
4	Gli1	ENSMUSG00000025407	.	****	***
4	Frzb	ENSMUSG00000027004	.	****	***
4	Mapk8ip1	ENSMUSG00000027223	****	.	.
4	Mdk	ENSMUSG00000027239	****	***	****
4	Bmp2	ENSMUSG00000027358	****	****	.
4	Pitx2	ENSMUSG00000028023	****	.	.
4	Rspo1	ENSMUSG00000028871	*	****	****
4	Ptpru	ENSMUSG00000028909	.	*	.

4	Draxin	ENSMUSG00000029005	.	.	****
4	Wnt5b	ENSMUSG00000030170	****	****	****
4	Dkk3	ENSMUSG00000030772	.	****	****
4	Dkkl1	ENSMUSG00000030792	.	.	****
4	Gpc4	ENSMUSG00000031119	.	****	****
4	Sfrp1	ENSMUSG00000031548	****	****	****
4	Nkd1	ENSMUSG00000031661	****	.	***
4	Mdfi	ENSMUSG00000032717	***	****	****
4	Wnt6	ENSMUSG00000033227	**	****	****
4	Vax2	ENSMUSG00000034777	****	.	****
4	Pygo1	ENSMUSG00000034910	****	****	****
4	Grhl3	ENSMUSG00000037188	.	**	***
4	Wnk2	ENSMUSG00000037989	****	****	*
4	Egr1	ENSMUSG00000038418	.	****	****
4	Six3	ENSMUSG00000038805	****	****	****
4	Rbms3	ENSMUSG00000039607	.	****	.
4	Hic1	ENSMUSG00000043099	.	*	***
4	Cxxc4	ENSMUSG00000044365	.	****	.
4	Shisa2	ENSMUSG00000044461	**	****	****
4	Dact1	ENSMUSG00000044548	.	****	.
4	Tmem88	ENSMUSG00000045377	****	****	****
4	Nog	ENSMUSG00000048616	***	.	.
4	Fzd9	ENSMUSG00000049551	.	**	.
4	Cthrc1	ENSMUSG00000054196	****	****	****
4	Fgfr3	ENSMUSG00000054252	****	.	****
4	Cdh3	ENSMUSG00000061048	****	**	****
4	Sox7	ENSMUSG00000063060	.	.	****
4	Celsr2	ENSMUSG00000068740	****	.	****
4	Nrarp	ENSMUSG00000078202	.	.	****
4	Dact3	ENSMUSG00000078794	***	.	****

C. BMP pathway: clusters and significance

**Cluster assignment**			**Pairwise DE significance**		
cluster	gene.name	gene.id	WT_KO	WT4_KO4	WT14_KO14
1	Itga3	ENSMUSG00000001507	.	****	****
1	Bmp8b	ENSMUSG00000002384	.	*	.
1	Cav1	ENSMUSG00000007655	.	****	.
1	Nanog	ENSMUSG00000012396	.	***	.
1	Tgfb3	ENSMUSG00000021253	****	****	.
1	Msx2	ENSMUSG00000021469	****	****	****

1	Wnt5a	ENSMUSG00000021994	****	.	****
1	Lrp2	ENSMUSG00000027070	****	****	.
1	Fbn1	ENSMUSG00000027204	.	.	****
1	Bmp2	ENSMUSG00000027358	****	****	.
1	Smad9	ENSMUSG00000027796	.	****	****
1	Lef1	ENSMUSG00000027985	.	****	****
1	Chrdl1	ENSMUSG00000031283	.	****	****
1	Zcchc18	ENSMUSG00000031428	***	.	*
1	Smpd3	ENSMUSG00000031906	****	****	.
1	Scx	ENSMUSG00000034161	.	****	.
1	Tgfb2	ENSMUSG00000039239	.	.	****
1	Nog	ENSMUSG00000048616	***	.	.
1	Sorl1	ENSMUSG00000049313	.	****	****
1	9330182L06Rik	ENSMUSG00000056004	****	****	****
1	Sox11	ENSMUSG00000063632	****	****	****
2	Gata6	ENSMUSG00000005836	****	****	.
2	Sfrp4	ENSMUSG00000021319	****	.	****
2	Hivep1	ENSMUSG00000021366	.	.	****
2	Fst	ENSMUSG00000021765	****	****	****
2	Hes1	ENSMUSG00000022528	****	****	****
2	Fstl1	ENSMUSG00000022816	.	****	.
2	Smad4	ENSMUSG00000024515	.	****	.
2	Smad7	ENSMUSG00000025880	****	****	.
2	Acvr1	ENSMUSG00000026836	****	.	.
2	Skil	ENSMUSG00000027660	.	****	****
2	Ilk	ENSMUSG00000030890	**	.	.
2	Cdh5	ENSMUSG00000031871	.	****	.
2	Neo1	ENSMUSG00000032340	****	.	.
2	Egr1	ENSMUSG00000038418	.	****	****
2	Smurf1	ENSMUSG00000038780	.	****	.
2	Fzd1	ENSMUSG00000044674	.	****	.
2	Acvr2a	ENSMUSG00000052155	.	.	****
2	Acvr2b	ENSMUSG00000061393	.	**	.
2	Hipk2	ENSMUSG00000061436	****	.	****
2	Kdr	ENSMUSG00000062960	****	****	.
2	Grem1	ENSMUSG00000074934	****	****	.
3	Htra1	ENSMUSG00000006205	.	****	****
3	Hfe	ENSMUSG00000006611	***	.	****
3	Sulf1	ENSMUSG00000016918	****	****	****
3	4632428N05Rik	ENSMUSG00000020101	****	****	.
3	Htra3	ENSMUSG00000029096	****	****	.

3	Tgfbr3	ENSMUSG00000029287	****	.	.
3	Bmper	ENSMUSG00000031963	.	****	****
3	Hoxa13	ENSMUSG00000038203	.	.	****
3	Gdf15	ENSMUSG00000038508	**	.	.
3	Id1	ENSMUSG00000042745	.	****	.
3	Dand5	ENSMUSG00000053226	.	****	.
3	Mir23a	ENSMUSG00000065611	.	*	.
3	Rgma	ENSMUSG00000070509	****	****	****
3	Foxd1	ENSMUSG00000078302	.	.	****
4	Acvrl1	ENSMUSG00000000530	.	.	****
4	Chrd	ENSMUSG00000006958	**	***	***
4	Tmprss6	ENSMUSG00000016942	.	.	*
4	Ror2	ENSMUSG00000021464	****	****	****
4	Bmp4	ENSMUSG00000021835	.	.	****
4	Rnf165	ENSMUSG00000025427	**	.	****
4	Eng	ENSMUSG00000026814	****	****	****
4	Sfrp2	ENSMUSG00000027996	.	****	****
4	Sfrp1	ENSMUSG00000031548	****	****	****
4	Comp	ENSMUSG00000031849	.	.	*
4	Bmp6	ENSMUSG00000039004	.	****	***
4	Nbl1	ENSMUSG00000041120	****	.	****
4	Fam83g	ENSMUSG00000042377	.	*	**
4	Zfp423	ENSMUSG00000045333	*	.	.
4	Msx1	ENSMUSG00000048450	****	****	****
4	Grem2	ENSMUSG00000050069	.	**	**
4	Bmpr1b	ENSMUSG00000052430	.	****	****
4	Kcp	ENSMUSG00000059022	.	.	****
